# Spatial structure: shaping the ecology and evolution of microbial communities

**DOI:** 10.1093/femsre/fuaf067

**Published:** 2026-02-09

**Authors:** Marcel Bäcker, Hilje M Doekes, Daniel R Garza, Jeroen Meijer, Simon van Vliet, Rosalind J Allen, Paulien Hogeweg, Bas E Dutilh, Bram van Dijk

**Affiliations:** Institute of Biodiversity, Ecology, and Evolution, Cluster of Excellence Balance of the Microverse, Friedrich Schiller University Jena, Rosalind-Franklin-Str. 1, 07745 Jena, Germany; Laboratory of Genetics, Wageningen University & Research, Droevendaalsesteeg 1, 6708 PB Wageningen, the Netherlands; Université Paris-Saclay, Institut national de recherche pour l’agriculture, l’alimentation et l’environnement (INRAE), PRocédés biOtechnologiques au Service de l’Environnement, 92761 Antony, France; Institute of Biodiversity, Ecology, and Evolution, Cluster of Excellence Balance of the Microverse, Friedrich Schiller University Jena, Rosalind-Franklin-Str. 1, 07745 Jena, Germany; Biozentrum, University of Basel, Spitalstrasse 41, 4056 Basel, Switzerland; Switzerland & Department of Fundamental Microbiology, University of Lausanne, Biophore, 1015 Lausanne , Switzerland; Theoretical Microbial Ecology Group, Institute of Microbiology, Cluster of Excellence Balance of the Microverse, Friedrich-Schiller University Jena, Buchaer Straße 6, 07749 Jena, Germany; Theoretical Biology and Bioinformatics, Utrecht University. Padualaan 8, 3584 CH Utrecht, the Netherlands; Institute of Biodiversity, Ecology, and Evolution, Cluster of Excellence Balance of the Microverse, Friedrich Schiller University Jena, Rosalind-Franklin-Str. 1, 07745 Jena, Germany; Theoretical Biology and Bioinformatics, Utrecht University. Padualaan 8, 3584 CH Utrecht, the Netherlands; Theoretical Biology and Bioinformatics, Utrecht University. Padualaan 8, 3584 CH Utrecht, the Netherlands

**Keywords:** spatial structure, community modeling, microbiome, microbial ecology, eco-evolutionary dynamics, biofilms

## Abstract

Most microbes grow in spatially structured communities, and this profoundly shapes their ecology and evolution. At the microscale, short interaction ranges and steep nutrient gradients underlie cross-feeding, quorum sensing, and niche construction, generating spatial patterns that influence microbial behavior, community assembly, and stability. Here, we review theoretical and experimental evidence for how spatial organization drives eco-evolutionary processes, including founder effects during colonization, allele surfing during range expansion, emergent patterns that facilitate multilevel selection, and the exploration of rare epistatic genotypes. While the ecological and evolutionary consequences of spatial structure at the microscale are becoming clearer, linking these processes across scales to predict community- and ecosystem-level outcomes remains a major challenge. Addressing spatial interactions explicitly in microbiome research will be key. Recent advances in computational modeling, cultivation approaches, and omics now offer unprecedented opportunities to meet this challenge, providing fresh insights into how spatial structure governs the organization and dynamics of the microbial world across scales.

## Teaser

Spatial structure naturally emerges in microbial communities, shaping growth, interactions, and evolution, and revealing how microscale processes scale up to influence community organization and ecosystem dynamics.

## Introduction

**Figure 1.**
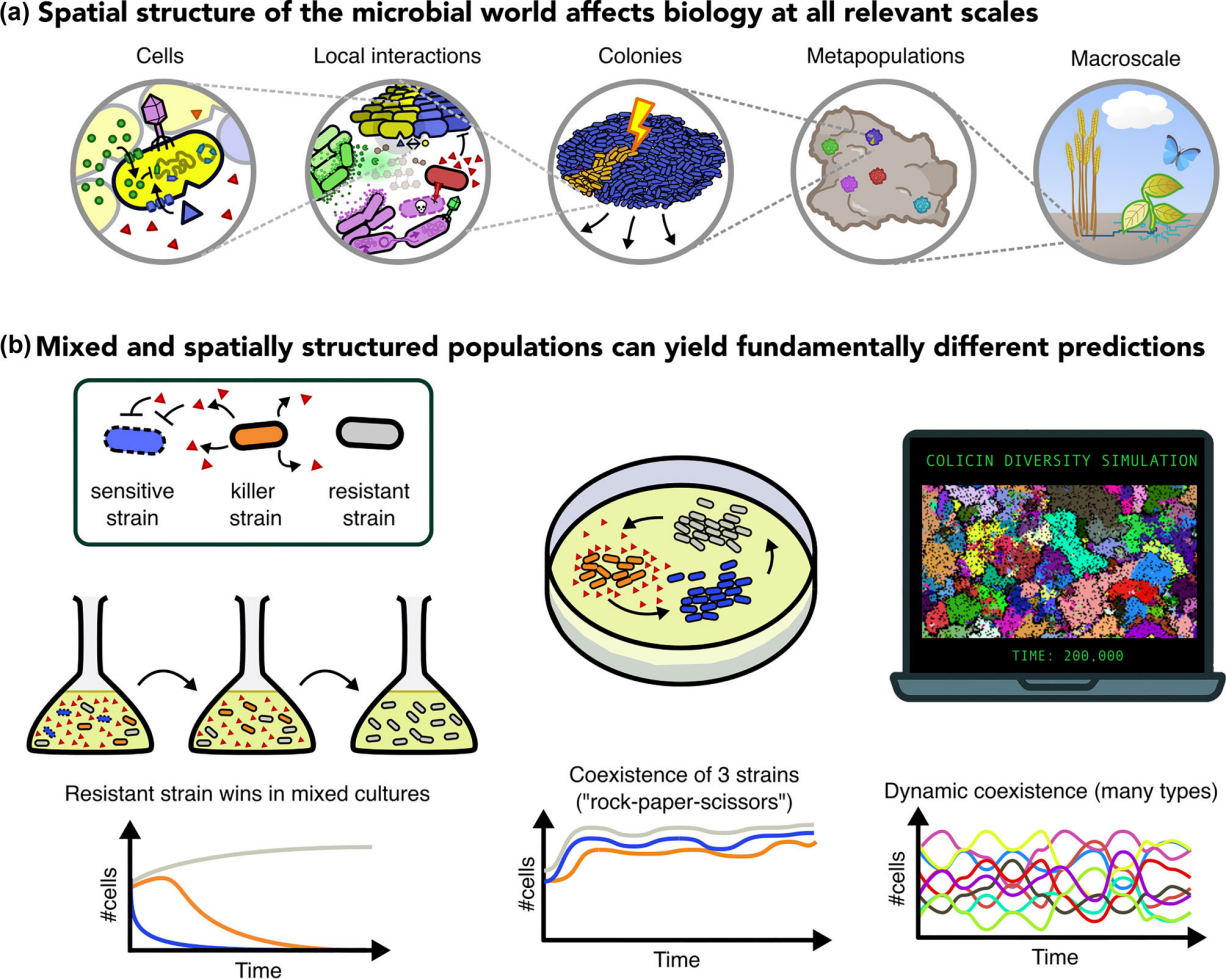
Spatial structure alters eco-evolutionary dynamics. (a) At every scale, spatial structure is known to play an important role in shaping microbial ecology and evolution. As microbes are intricately connected to all larger life forms, these details may eventually shape entire ecosystems. (b) Evolutionary outcomes depend on spatial structuring and local interactions. A well-studied example is found in killer-sensitive-resistant (KRS) dynamics (Kerr et al. 2002), where mixed systems (flasks or mixed plates) show the survival of only resistant strains (shown in grey), whereas spatially structured systems, such as static agar plates or spatially explicit computational models (Pagie and Hogeweg 1999), show that KRS-systems result in “rock-paper-scissors” dynamics.

Microbiological systems show **spatial structure** on many scales. At the microscale, colonies and biofilms form structured populations shaped by nutrient flow and cell-cell signaling, while nutrient gradients drive metabolic specialization. Zooming out, we find that the microbial world is often fragmented into sparsely connected subpopulations spanning habitats such as marine snow particles, gut crypts, and soil particles. These spatial arrangements shape resource availability, interaction outcomes, and the evolutionary trajectories of microbial populations. Localized processes can propagate to larger scales, up to the scale of the ecosystem (Figure 1a). Because microbes lie at the heart of fundamental ecosystem services like nutrient cycling (Datta et al. 2016, Nguyen et al. 2022, Camenzind et al. 2023) and bioremediation (Philippot et al. 2024, Rosa-Masegosa et al. 2024), as well as human health (Azimi et al. 2022, Lotstedt et al. 2023, Philippot et al. 2024), their spatial patterns may play a larger role than previously recognized. Elucidating how spatial structure emerges in microbial ecosystems and drives their eco-evolutionary dynamics is essential to understanding how microbes shape the biosphere.

While there is growing recognition that microbial spatial structure can shape broader ecological and evolutionary outcomes, many laboratory experiments intentionally strip away spatial complexity, creating homogeneous environments where all cells are assumed to behave identically. This practice makes it easier to focus on some specific mechanisms, but well-mixed conditions stand in stark contrast to natural systems, where most microbes grow in dense, structured communities shaped by spatial separation and local gradients (Flemming and Wuertz 2019, Baker et al. 2024, McCallum and Tropini 2024). Models and experiments that account for spatial structure often yield very different outcomes compared to well-mixed systems (Figure 1b), especially for key ecological interactions like **mutualism** and **antagonism** (terms in bold are explained in Table 1) (Pagie and Hogeweg 1999, Czárán et al. 2002, Kerr et al. 2002, Czárán and Hoekstra 2009, Colizzi and Hogeweg 2016a, 2016b , Nadell et al. 2016, Vetsigian 2017, Doekes et al. 2019, France and Forney 2019, Smith et al. 2020b, Meijer et al. 2020, Shibasaki and Mitri 2023, Booth et al. 2024, Fullmer et al. 2024, Takeuchi et al. 2024).

**Table 1.** Glossary of terms

*Allele surfing*	The stochastic increase of allele frequency at an expanding population front due to repeated founder effects
*Antagonism*	An interaction where one microbe negatively affects the growth, survival, or reproduction of another
*Auxotrophy*	The inability to synthesize an essential organic compound, requiring its uptake
*Bottom-up*	Focusing on microbial inputs, such as spatio-temporal growth, competition, or metabolite exchange, and constructing them upwards to large-scale outputs, like community composition
*Cross-feeding*	Feeding of the metabolic byproducts of another cell
*Eco-evolutionary tunneling*	Crossing of a genetic or ecological barrier towards a higher fitness or ecologically more stable state that is inaccessible by step-wise evolution
*Exploratory models*	Models aimed to explore fundamental eco-evolutionary principles usually focused on mechanisms rather than parameters
*Founder effects*	When only a few cells found a new population, stochastic effects are amplified, resulting in phenotypic noise and genetic drift
*Local interactions*	Interactions that only affect the immediate environment of a cell
*Mutational jackpot*	Mutations that generate large descendant lineages by chance rather than selective advantage
*Mutualism*	An interaction where one microbe positively affects the growth, survival, or reproduction of another
*Niche construction*	Modifying the immediate environment, so that the niche conditions change, e.g. a biofilm to protect an anaerobe against oxygen
*Predictive models*	Models focused on practical applications and forecasting real-world ecosystems usually focus on parameters rather than mechanisms
*Public good*	Functions or products of a cell that provide a collective benefit, e.g. chitinase or chitin oligomers produced by chitinases
*Quorum sensing*	The process of sensing and denoising of environmental signals and choreographing population behavior mediated by an accumulating autoinducer
*Range expansion*	Moving on a solid surface simply by reproducing and continuously shoving cells forward
*Spatial structure*	Biotic: the self-assembled structure of microbes in space, e.g. by clonal assortment Abiotic: the physical space confining an organism, e.g. pore space
*Top-down*	Focusing on microbial outputs, such as nutrient fluxes or community composition, and projecting downward to infer the inputs, i.e. local processes and interactions, that may have produced them

Although migration, bottlenecks, and other processes disturb the spatial distribution of microbes, virtually all microbiomes (natural or otherwise) self-organize into spatially structured systems (Table 2). Structure naturally and repeatedly emerges **bottom-up**. Theoretical models have been instrumental in clarifying the role of emergent spatial patterns in shaping microbial ecology and evolution, consistently showing that simple rules at the microscale do not necessarily result in simple outcomes at the macroscale (Reynolds 1987, Schmickl et al. 2016). Furthermore, having full knowledge of individual cells, their genotypes, and subpopulation sizes may still be insufficient to predict community-level behavior without an understanding of the spatial context and self-organization. With recent technological advances, experiments are now confirming these predictions, revealing the importance of spatial structure for many ecological phenomena, including cooperation (Momeni et al. 2013, Dal Co et al. 2020, van Vliet et al. 2022, Jeckel et al. 2023, Luo et al. 2024), the spread of mutations and genes (Fusco et al. 2016, Ruan et al. 2022, Michaelis and Grohmann 2023), and **cross-feeding** (Dal Co et al. 2019b, Borer et al. 2020, Goldschmidt et al. 2021). Processes at different spatial scales are tightly coupled: microscale interactions like nutrient gradients, **allele surfing**, or particle colonization can propagate upward to shape population structure, biogeochemical fluxes, and even global carbon cycling (Gralka et al. 2016, Abs et al. 2020, Ebrahimi et al. 2022, Nguyen et al. 2022). Conversely, ecosystem-level perturbations cascade down to restructure **local interactions**, underscoring the need for a truly multiscale perspective. To improve predictions of microbial eco-evolutionary dynamics and their impact, we need to understand how these many non-linear, spatially separated processes integrate and affect the world around us (Friedman et al. 2017, Batsch et al. 2024, Lopes et al. 2024).

**Table 2.**
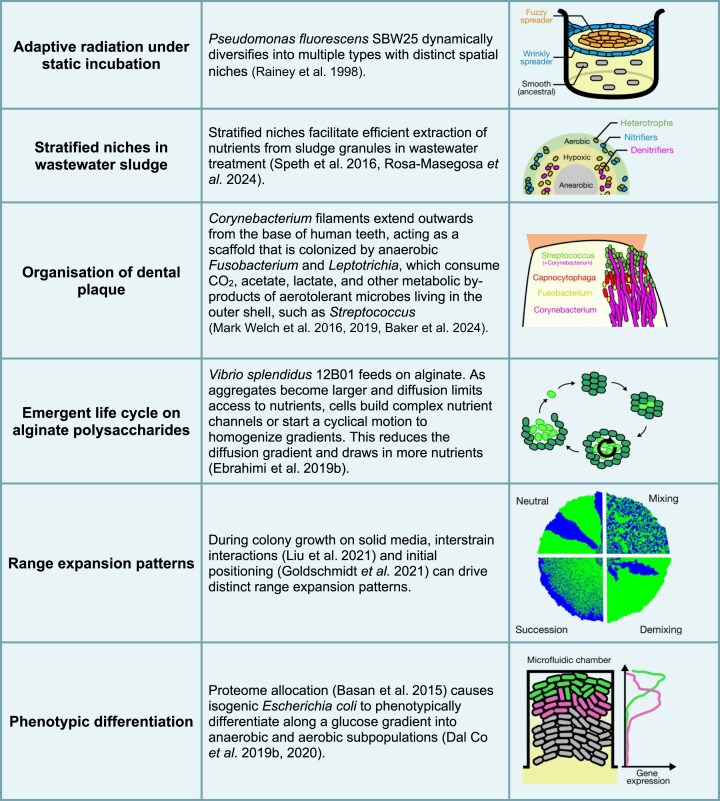
Examples of spatial structure in nature and in the lab.

Similar challenges arise when trying to understand microbial systems **top-down**. For instance, biogeographical studies and metagenomics offer insights into the large-scale properties of microbial systems (Gralka et al. 2023). Yet uncovering underlying causal mechanisms from these large-scale datasets remains challenging due to their complexity, the frequent lack of time-series data, and our limited functional understanding of microbial sequence space. Moreover, these studies usually require invasive sampling: extracting DNA, proteins, or metabolites disrupts the community (Ladau and Eloe-Fadrosh 2019), making it challenging to track dynamics over time. These factors can contribute to poor replicability, as spatial heterogeneity and sampling biases may yield variable outcomes across replicates or studies. The role of spatial structure can, however, be investigated with carefully designed, well-controlled experiments. In particular, compost mesocosms revealed highly parallel dynamics across replicates (Meijer et al. 2025), highlighting the potential of controlled systems to uncover generalizable eco-evolutionary patterns, even in complex microbiomes. Yet, microbial composition becomes increasingly unpredictable at the micrometer scale (Bach et al. 2018, Wilpiszeski et al. 2019, Averill et al. 2021).

In summary, to better understand and predict microbial ecology and evolution in nature, we must grasp not only the role of spatial structure but also how its impacts translate across scales. Spatially resolved models and experiments have the potential to address many critical eco-evolutionary questions. For instance, what mechanisms allow functionally similar species to stably coexist? Why do many bacteria lose key biosynthetic pathways and depend on others for essential metabolites? How do functional redundancies and dependencies impact ecosystem robustness? How does the rapid and continuous evolution of microbial interactions shape spatial community dynamics? These questions lie at the heart of this review.

In this review, we synthesize experimental data and theory on the ecology and evolution of spatially structured microbial ecosystems. We discuss how spatial structure shapes, and is shaped by, molecular, ecological, and evolutionary mechanisms, offering a mechanistic perspective to the biogeographical description of microbiomes (Mark Welch et al. 2016, Dick 2019, Mark Welch et al. 2019, Azimi et al. 2022, Baker et al. 2024, McCallum and Tropini 2024, Philippot et al. 2024) and to the metabolic perspective described elsewhere (Dal Co et al. 2023, Huelsmann et al. 2024, Henderson et al. 2025). Starting at the microscale, we examine how local interactions between individual cells shape spatial organization and ecological dynamics. We then move up to the scale of populations, exploring how interactions among different populations give rise to structured communities and metapopulations, and how these dynamics influence diversity and evolution. We conclude at the macroscale, considering the challenges in modeling and predicting ecosystem behavior across scales, and highlighting recent developments in spatial modeling, cultivation, and omics that open up new research avenues.

## Local interactions

Even in systems that are typically considered well-mixed, such as the ocean (Box 1) or wastewater treatment facilities, microbes often occur in highly structured, localized environments at the microscale (Speth et al. 2016). As cells divide, populations self-organize into spatially separated clusters where genetically similar cells are located in each other’s vicinity (van Vliet et al. 2018, Borer et al. 2020). Spatial arrangement emerges from a combination of environmental constraints, including extracellular matrices or surfaces, diffusion, decay, and wash-out, and microbial activities, including uptake, growth, and physical crowding (Nadell et al. 2016, Jo et al. 2022, Dal Co et al. 2023, Henderson et al. 2025). Interactions, therefore, differ across local environments.

Box 1. **Spatial structure at all scales of marine particle ecology**

Despite currents, tides, and extensive turbulence, the open ocean is not well-mixed; marine microbes see a “sea of gradients'' orchestrating their organization in a multitude of microniches and spatial structures, ranging from largely unstructured planktonic growth to highly structured biofilms on particulate organic matter (Stocker 2012) or in association with phytoplankton (Martínez-Pérez et al. 2024). Marine snow consists of aggregated, decaying organic material sinking in the oceanic water column with ∼25 particles per liter on average (Ebrahimi et al. 2022), thought to be colonized by highly structured bacterial populations (Zomer et al. 2024). Chitin is the second most abundant polysaccharide on earth, found in arthropods, fungi, and algae, and makes up a large fraction of marine snow (Souza et al. 2011). Starting from a pristine chitin particle, the initial microbial colonization is short-lived and as diverse as bulk seawater (Datta et al. 2016). At the scale of individual cells, primary degraders encoding chitin degradation approach particles via chemotaxis. Due to functional redundancy, the taxonomic identity of the initial colonizer is, to a degree, stochastic, imposing far-reaching priority effects on the local ecology since most early degrader populations are founded by one cell and thus clonal (Schwartzman et al. 2022). Smaller groups of cells can share public goods, including exoenzymes. The cost-efficiency of polysaccharide degradation scales with population size (D’Souza et al. 2023) because cells profit from their exoenzymes being used by related cells and the reduction of diffusional losses in the structured populations. However, the rapid uptake of degradation products produces steep gradients radially from the surface, and with time, populations outgrow the interaction ranges. Aggregates growing on alginate circumvent these gradients by constructing microfluidic channels or rotating inside the aggregate to homogenize gradients (Table 2) (Ebrahimi et al. 2019a). Similarly, monocultures of *Vibrio* sp. 1A01 show cells attaching, producing chitinase to engage in exponential growth, and maintaining exponential growth by constantly shedding, i.e. detaching, leaving chitinase behind, and thus maintaining the nutrient supply for a subpopulation (Guessous et al. 2023). Two species can coexist merely by differentiating around such dispersal parameters (Ebrahimi et al. 2022). Chitinases and oligomeric byproducts inevitably attract exploiters, bacteria that cross-feed on short chitin fragments but not chitin (Pontrelli et al. 2022). Presumably due to competition for space (D’Souza et al. 2023) and nutrients, including with exploiters, degraders start to disappear from the particle (Smriga et al. 2016, Stubbusch et al. 2024), slowing down particle degradation (Enke et al. 2018). Exploiters metabolize primary degradation products and secrete secondary byproducts that attract scavengers, further increasing the number of species supported by this microecosystem (Pontrelli et al. 2022, D’Souza et al. 2023). Moreover, natural marine snow particles are usually complex blends of polymers, and their assembly can be modularly combined from pure particles (Enke et al. 2019). Repeated degradation-dispersal cycles and the small number of effectively growing cells during founding or due to gradients amplify founder effects, i.e. stochasticity in the evolution of a population and extreme dependence on the initial colonizers, so that earlier colonizers can outcompete less fit competitors (Eigentler et al. 2022, Schwartzman et al. 2022). The coexistence of degraders, exploiters, and scavengers in the metacommunity hinges on their distribution in space on different particles (Pollak et al. 2021). From a niche perspective, particle degradation supplies the energy to heavily modify the immediate environment by building biofilms, secreting protective and nutrient-scavenging proteins, or engaging in other forms of niche construction. Indeed, the chitin “rafts” hypothesis proposes that the evolution of chitin utilization and attachment in picocyanobacteria 570 Million years ago allowed the transition from living in photosynthetic benthic mats to living as planktonic cells by providing “temporary” refuge from unsuitable environmental conditions over millions of years until the acquisition of planktonic genes and subsequent streamlining of chitin tropism was completed (Capovilla et al. 2023).

### Interaction ranges

**Figure 2.**
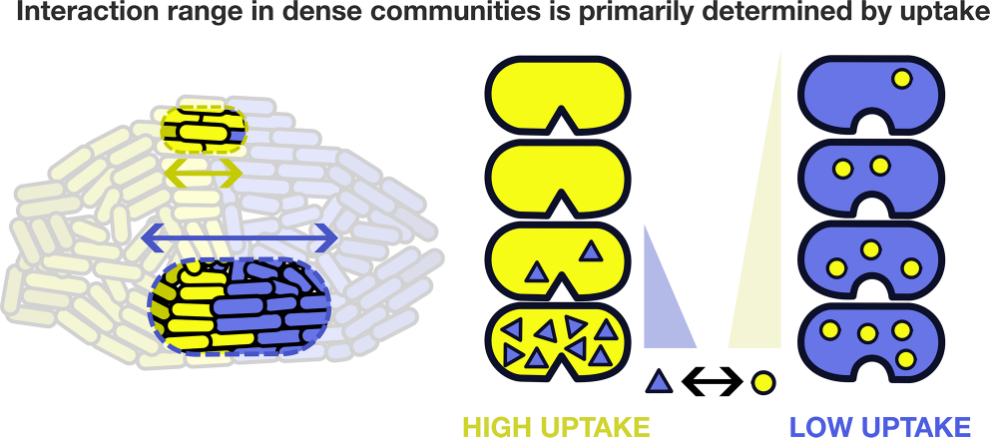
Interaction ranges in microecology are defined by the uptake, leakage, or degradation rates of compounds. The triangles and circles represent compounds such as nutrients or signals. The uptake rate of a compound determines its interaction range. High uptake shortens interaction ranges, as shown for the yellow cells with the triangular compounds, whereas low uptake rate extends interaction range, as shown for the blue cells and the circular compound.

Recent research has shown that most microbial interactions occur over remarkably short spatial scales. While one might expect short-range interactions in the context of contact-dependent processes like type 6 secretion systems (Stubbusch et al. 2025) or metabolite sharing via intercellular nanotubes (Xiong et al. 2018), even interactions that are mediated by diffusible compounds can be remarkably short-ranged. Examples of microscale interactions in microbial ecology include the cross-feeding of metabolites (Dal Co et al. 2019b, Goldford et al. 2018, Dal Bello et al. 2021, Ona et al. 2021, Wang 2025), local antagonism via antibiotic production (Cornforth and Foster 2015, Sharma and Wood 2021), sharing of extracellular enzymes (Garcia-Garcera and Rocha 2020, D’Souza et al. 2023), and quorum sensing (Darch et al. 2018, van Gestel et al. 2021). Although small molecules diffuse rapidly across micrometer distances (e.g. the 3D root mean squared displacement of glucose in water is about 60 µm in one second (Kreft et al. 2012)), the rapid uptake of metabolites by nearby cells creates steep concentration gradients around the source. This scavenging effect can restrict the effective metabolic interaction range to only a few cell lengths (Figure 2) (van Tatenhove-Pel et al. 2021b, Dal Co et al. 2020, 2023). The importance of source-sink dynamics is further highlighted by the drastic difference in range between signaling systems that use absorbing vs non-absorbing signaling molecules, with effects spanning from a few cell lengths to several hundred micrometers, respectively (Darch et al. 2018, van Gestel et al. 2021). Even larger extracellular elements like membrane vesicles (Schwechheimer and Kuehn 2015, Reyes-Robles et al. 2018) and virus particles (Simmons et al. 2018) are likely to act locally, as they will encounter and interact with nearby cells first, making long-range effects unlikely. In summary, the effective local playing field of microbial ecology is often limited to only a few cell lengths. This implies that even within a population of millions of cells, each bacterial individual interacts only with a relatively small local neighborhood of nearby cells.

The interaction range is not only shaped by cellular activity but also by the physical environment. For example, in structured environments like soil, the majority of bacteria reside in small, disconnected patches within clay microaggregates (50–250 µm), reducing connectivity and dispersal between subpopulations (Rillig et al. 2017, Bach et al. 2018, Wilpiszeski et al. 2019, Hartmann and Six 2022). Taking account of pore space and diffusion constraints, the effective interaction range in soil is estimated to be ∼20 µm (Raynaud and Nunan 2014). Similar physical constraints exist in porous or flow-subjected lab systems similar to gut crypts, where local quorum sensing hotspots can emerge (Scheidweiler et al. 2024).

In growing microcolonies, short-range interactions are reinforced by cellular ancestry and spatial history. Cells tend to remain close to their progenitors, forming lineages with shared phenotypic states, since proteins are inherited during division (Veening et al. 2008, Robert et al. 2010, Hormoz et al. 2015, van Vliet et al. 2018). Within microcolonies, spatial gradients in resource availability and signaling molecules often emerge, driving phenotypic diversification and the emergence of new interactions (Julou et al. 2013, van Vliet et al. 2018). For example, an oxygen gradient from the microaerobic flocs and granule surface to an anaerobic granule core allows both oxygen-dependent nitritation and oxygen-sensitive anammox reactions in a single wastewater treatment reactor (Speth et al. 2016). These spatial gradients may lead to the formation of quasi-fragmented subpopulations that primarily interact within discrete patches (Verdon et al. 2025).

### Cross-feeding interactions

Microbial communities commonly share nutrients, genetic information, and proteins among their members, resulting in complex ecological interactions. While some studies argue that microbial cooperation is rare and that **antagonistic interactions** dominate (Palmer and Foster 2022), the frequent occurrence of **auxotrophic** lineages in nature (Kost et al. 2023) suggests that many microbes rely on others for essential metabolites. Microbes can overcome metabolic limitations through a mixture of cooperative and competitive strategies. We therefore focus on the mechanisms and evolutionary origins of these exchanges rather than on broad labels such as cooperation or antagonism.

In the simplest case, a donor cell might simply secrete metabolites that other cells use. For example, short-chain fatty acids may be secreted during nutrient-limited fermentation (overflow metabolism) and respired by neighboring organisms (Basan et al. 2015, Pande and Kost 2017, Amarnath et al. 2023). Beyond unidirectional exchange, microbes can interact bi-directionally, for example by participating in collective metabolism where each microbe can only fulfill partial metabolic reactions in a pathway (Ona et al. 2021, Huelsmann et al. 2024). However, microbes can also overcome nutrient limitations by killing and lysing nearby competitors, gaining access to the released cytoplasmic contents. For example, phage-mediated release has been shown to promote growth by releasing limiting factors such as amino acids and vitamins (Pherribo and Taga 2023, Wienhausen et al. 2024). Marine Vibrio species engage in active killing through type 6 secretion systems (T6SS) (Stubbusch et al. 2025) to resolve their nutrient limitations. All of these strategies presuppose a long-term spatial co-association of the interaction partners. This raises the question of whether these behaviors can persist in natural environments or whether they represent transient states that emerge readily but are equally prone to collapse.

In a long-term evolution experiment (LTEE) with *E. coli*, metabolic dependencies evolved and persisted without spatial structure (Rozen and Lenski 2000), even reemerging rapidly when one partner is removed (Ascensao et al. 2024). Here, strains specialize on either fermenting glucose to acetate or on respiring acetate, generating two temporal niches that favor coexistence (Hui et al. 2015, Schink et al. 2022, Mukherjee et al. 2023). Similar dynamics are expected for many substrates, as models predict thousands of carbon source pairs that could sustain cross-feeding of this kind (Langille et al. 2018). These interactions redistribute metabolic functions and expand the community’s niche space beyond what isolated species could achieve (Ona et al. 2021, Huelsmann et al. 2024). Thus, even well-mixed systems show that microbes rapidly adjust their metabolic strategies to reduce niche overlap, thereby promoting stable coexistence (Rainey and Travisano 1998, France and Forney 2019, Blasche et al. 2021, Muratore et al. 2022). This raises two key questions: what ecological and evolutionary mechanisms drive rapid **niche construction** and emergent dependencies, and are these dependencies expected to emerge in spatially structured systems as well?

The leading model for the evolution of metabolic dependencies is the Black Queen (BQ) model (Morris et al. 2012, Hesse and O’Brien 2024), which proposes that interdependencies naturally emerge in ecosystems through gene loss of costly, publicly accessible functions (**public goods**) that are performed by other community members. These functions may range from extracellular polymer degradation to intracellular processes like antibiotic detoxification, which can lead to cross-protection (Sorg et al. 2016). Even seemingly private functions like macromolecular biosynthesis eventually become part of the public domain after cell lysis. For example, a recent study identified how a bacterial strain synthesizes a ligand that promotes another strain to synthesize vitamin B_12_, which is released through phage-mediated lysis (Wienhausen et al. 2024). This highlights that cell death and lysis may play key roles in shaping dynamics through the lens of the BQ model, contributing to a complex and still poorly understood web of trophic and social interactions. Just as the individual cells of multicellular organisms cannot survive alone, microbes living in dense, spatially structured, polymicrobial communities appear to quickly evolve interdependencies, often at distinct spatial scales depending on the nature and diffusibility of the exchanged functions (D’Souza and Kost 2016, Ona et al. 2021, Giri et al. 2022, Boza et al. 2023).

**Figure 3.**
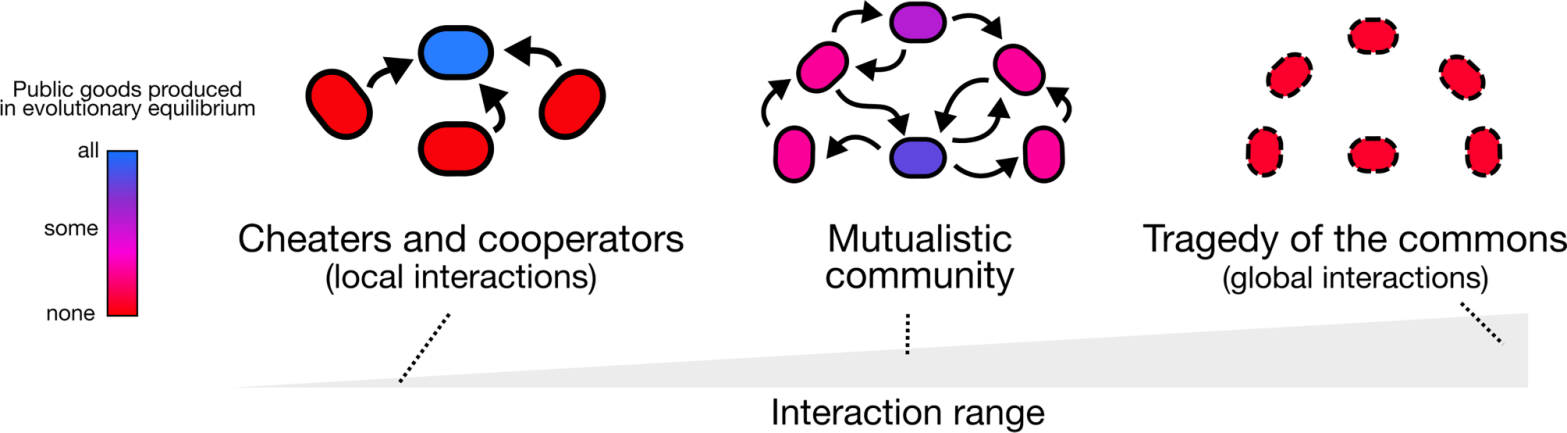
Black Queen dynamics have distinct eco-evolutionary endpoints depending on the interaction range. Short interaction ranges shift the interacting strains to accumulate public functions within a central strain, whereas intermediate interaction ranges give rise to a highly interconnected network of public good producers. At high interaction ranges that approach a well-mixed system, communities relying on public goods ultimately collapse (Fullmer et al. 2024).

Traditionally, the BQ model attributes loss of function to natural selection acting at the level of individual lineages, particularly when the function is metabolically costly. This interpretation assumes that large population sizes minimize the effects of genetic drift. However, even in infinitely large populations, weakly selected traits can be lost through mutational meltdown (Eigen et al. 1988, van Dijk et al. 2020). Further, in spatially structured populations where selection acts locally and competition is primarily among neighbors, the influence of genetic drift may be far greater than population size alone would suggest. On the one hand, this increased level of drift may open up a far richer landscape of eco-evolutionary outcomes, but may hinder the stability of said outcomes. Recent work by Fullmer et al. (2024) shows that tuning the spatial range of BQ interactions results in distinct community outcomes: canonical cheaters and cooperators, fully interdependent communities, and collapse of entire ecosystems (Figure 3). When the range of spatial interactions is large, individual cells have an enhanced probability of having neighbors that sustain their growth. This concept of neighborhood uncertainty has been shown to change the predictions of the BQ model (Stump et al. 2018), and helps promote complex and partially redundant metabolic interactions within ecosystems, especially when interaction ranges are limiting (Fullmer et al. 2024). Interestingly, in spatially explicit simulations where interaction ranges are short and metabolite exchange is highly local, both metabolic autonomy and interdependency have been shown to be viable strategies (Meijer et al. 2020).

The above-mentioned modeling predictions are backed by ancestral state reconstructions on reference genomes from the Earth Microbiome Project, suggesting that auxotrophic communities in nature are far more common than previously recognized (Machado et al. 2021). Furthermore, data indicate that spatially structured biomes contain more extracellular proteins than well-mixed ones (Garcia-Garcera and Rocha 2020), consistent with the underlying idea that spatial structure stabilizes extracellular, cheatable functions. Recent work also shows how spatial structure may hinder Black Queen dynamics; *E. coli* auxotrophs may fail to invade when leakage of amino acids is limited (Ramesh et al. 2025). In other words, while spatial structure clearly plays a critical role in shaping the evolution of cross-feeding and metabolic interdependence in microbial communities, the precise effect can be highly context-dependent. Incorporating spatial structure into models and experimental protocols will allow us to better understand the ecological and evolutionary forces that drive the emergence, maintenance, and fragility of microbial interdependencies.

### Niche construction

As microbes adapt to their environment, they also change it. The ecological niche of an organism is traditionally defined as the set of conditions enabling its growth, but these conditions change over time. Darwin already noted how earthworms alter soil structure in ways that benefit their own physiology (Darwin 1892). Modern niche construction theory formalized this idea by defining niche construction as the process through which organisms, via their metabolism, activities, or choices, modify their own or others’ niches in ways that change one or more of the selection pressures acting on populations, whether through physical alteration of the environment or through relocation (Levins and Lewontin 1985, Odling-Smee et al. 1996, Laland et al. 2016). However, an ongoing challenge is that almost any organismal activity can be interpreted as niche construction, which risks blurring the conceptual boundaries of the term. To maintain analytical clarity, many authors therefore use the term in a narrower sense, reserving it for traits that specifically evolved to modify the organism’s environment in ways that enhance its fitness, potentially affecting other organisms, rather than for incidental byproducts of life (Okasha 2005, Kylafis and Loreau 2011, Scott-Phillips et al. 2014). Classic examples in animals and plants include dam building in beavers and nodule-formation in legumes (Naiman et al. 1988, Oldroyd et al. 2011). For microbes, broad interpretations include the cyanobacteria-driven, planetary oxygenation of the early Earth (Schirrmeister et al. 2015), whereas the secretion of biosurfactants that modify surface properties to enable swarming on surfaces exemplifies the narrow definition of niche construction (Kearns and Losick 2003, Kearns 2010, Flemming and Wuertz 2019). The following sections illustrate how microscale niche modifications interact with spatial structure in microbial communities.

Pristine environments, i.e. environments that start free of microbes and are shaped strongly by early colonizers, provide the clearest view of how niche construction interacts with spatial structure. Early colonizers gain privileged access to space and resources and their microbial activity creates steep microscale gradients that reinforce this initial advantage. For example, nutrient leakage, especially of glutamine, around emerging root tips generates localized hotspots that attract chemotactic bacteria and allow rapid proliferation before other taxa arrive (Tsai et al. 2025). A similar picture holds in sterile human tissues at the onset of infection, where the first invading lineages can alter local nutrient profiles and immune responses, thereby shaping which microbes can establish later (Azimi et al. 2022). Such spatially mediated priority effects are likely widespread because any initially microbe-free and physically heterogeneous habitat offers opportunities for early-arriving taxa to influence the conditions encountered by later ones (Debray et al. 2022).

Polysaccharide particles released from decomposing algae (Box 1) are another vivid example: early colonizers transform the particle chemically and physically, shaping which other microbes can join later (Datta et al. 2016, Debray et al. 2022). Primary degraders produce sugars that may attract secondary consumers (Pollak et al. 2021, Pontrelli et al. 2022), which may then attract tertiary and subsequent consumers (Hehemann et al. 2016). Niche constructions may be chemical in nature, e.g. changing pH (Ratzke and Gore 2018, Ratzke et al 2020), but more surprising strategies, such as decorating the biofilm with phages targeted against competitors (Bond et al. 2021) have also been discovered. Cooperative interactions can also trigger niche construction. *E. coli* cells respond rapidly to reactive oxygen species; when a biofilm is exposed to hydrogen peroxide, peripheral cells detoxify the compound, generating protective gradients for the community (Choudhary et al. 2023). Similarly, host–microbe interactions can be shaped by niche construction through division of labour. During plant infection, *Pseudomonas syringae* subpopulations specialize such that T3SS-expressing cells suppress host immunity as a public good, enabling motile flagellated cells to exit the tissue (López-Pagán et al. 2025). Interspecific niche construction has been shown for biofilm formation. When *Pseudomonas* grows in artificial pores, its biofilm formation is promoted by co-occurring *Arthrobacter* that hydrolyze an inhibitor and subsequently profit from public goods released from the biofilm (Wu et al. 2023). Microbes do not simply occupy niches but actively construct them (Aaby and Desmond 2021), with their progeny also inheriting these niche conditions (Guessous et al. 2023).

Although well-mixed experimental systems can foster the emergence of simple ecosystem interactions through niche construction (Rozen and Lenski 2000), in spatially structured environments, where interactions are inherently local, the feedback between microbial activity and environmental modification can give rise to a mosaic of emergent niches. In such settings, spatial separation allows distinct microenvironments and selection pressures to form side by side, fostering ecological and evolutionary diversification (Rainey and Travisano 1998). These dynamics challenge the classical view of niches as fixed, global properties. Instead, niches emerge locally and dynamically, co-constructed through iterative, space-dependent interactions between organisms and their surroundings. This may help explain why studies that embrace spatial structure, like compost communities grown on cellulose paper (Rainey and Quistad 2020), maintain high diversity, and even see it increase over long times.

Computational models have also helped to unravel the interplay between spatial niche construction and emergent patterning. For example, the *Virtual microbes* framework (van Dijk et al. 2019) has explored how microbes evolve in dynamic, spatially structured environments. By simulating an artificial chemistry where microbes have to produce a set number of building blocks for growth, the work shows that cross-feeding can evolve *de novo* even when not promoted by costs for producers (Meijer et al. 2020). Here, environmental feedback loops play a critical role, which was later also shown in experimental studies (Henderson et al. 2025).

### Quorum sensing

Cross-feeding and niche construction reveal that microbial traits are not always private: the effects of microbial behavior often spill over into the environment, creating shared or semi-shared benefits. The production of costly public goods (e.g. antibiotic-degrading enzymes or iron-scavenging compounds) can be exploited by non-producing cheaters, especially in well-mixed environments like shaking flasks, where benefits can reach unrelated cells (West et al. 2006). Spatial structure limits this mixing and tends to confine the benefits of public goods to nearby cells, likely to be close relatives (kin), thereby stabilizing cooperation (Mund et al. 2017). To reinforce this kin bias, the production of public goods is often regulated by **quorum sensing** (QS), which coordinates gene expression among clonemates and can act as a proxy for kinship (Schluter et al. 2016).

In QS, cells produce a signaling molecule, which is used to regulate certain behavior, such that this behavior only occurs at high cell densities (Waters and Bassler 2005). While often studied in large populations in shaking flasks, QS can also operate effectively in spatially structured environments where local assortment and confinement generate similarly high local densities (Boedicker et al. 2009). QS can be a cross-species phenomenon: some quorum-sensing molecules, such as the metabolite AI-2, are near-universally sensed across the bacterial domain even by non-producing species (Zhang et al. 2020), and cheap (< 1 ATP) to produce (Keller and Surette 2006), implying a complex interplay between population density, population composition and signaling in polymicrobial communities (Pereira et al. 2013). For example, models of anticompetitor toxin regulation have found that regulation can readily evolve in response to a universal QS molecule (Doekes et al 2019), but not in response to a cost-bearing, more specific signal (Czárán and Hoekstra 2007). As mentioned above, signals such as for QS act on different length scales depending on how the signals are processed at the cellular level. If QS signaling molecules are absorbed, they create steep gradients and are of short range, whereas non-absorbing systems are of long range (van Gestel et al. 2021). Beyond range, QS responses are often heterogeneous: signals can generate bimodal activation, with some cells fully activating while others remain unresponsive. Strikingly, heterogeneity can translate into coupled dynamics between neighboring populations. Recent single-cell microfluidics experiments in *Staphylococcus aureus* showed that agr-type quorum-sensing interactions do not always lead to uniform activation across populations. Instead, depending on how much the different agr systems respond to each other’s signals and on the starting conditions of the populations, several outcomes are possible: one strain can consistently dominate, the two can take turns being active over time, or both can remain active but in separate neighboring regions (Bär et al. 2024).

As well as serving as a mechanism for detecting cooperator density, QS also functions as a tool for environmental sensing, especially in assessing the diffusivity of the surrounding microenvironment (Redfield 2002, Mund et al. 2017). As cellular signals are inherently noisy, QS can reduce noise by aggregating inputs from multiple cells that experience similar environmental conditions. This wisdom-of-the-crowds effect can enhance the accuracy of collective decision-making (Moreno-Gamez et al. 2023). Such functionality may explain why QS has been found to regulate not only public goods but also private goods, and offers adaptive advantages in diverse settings (Wetherington et al. 2022, Guessous et al. 2023, Stubbusch et al. 2024).

To summarize, local interactions show that microbes experience their environment over very short distances, where steep gradients, restricted diffusion, and physical crowding structure metabolic exchange, signaling, antagonism, and gene flow. These interactions create patchy ecological landscapes even at the micrometer scale, and establish strong feedbacks between phenotype, ancestry, and microenvironment. Such spatial constraints set the conditions under which small groups of cells begin to form structured multicellular assemblies. In the next section, we examine how these local processes scale up as communities grow into colonies and biofilms.

## Colonies

**Figure 4.**
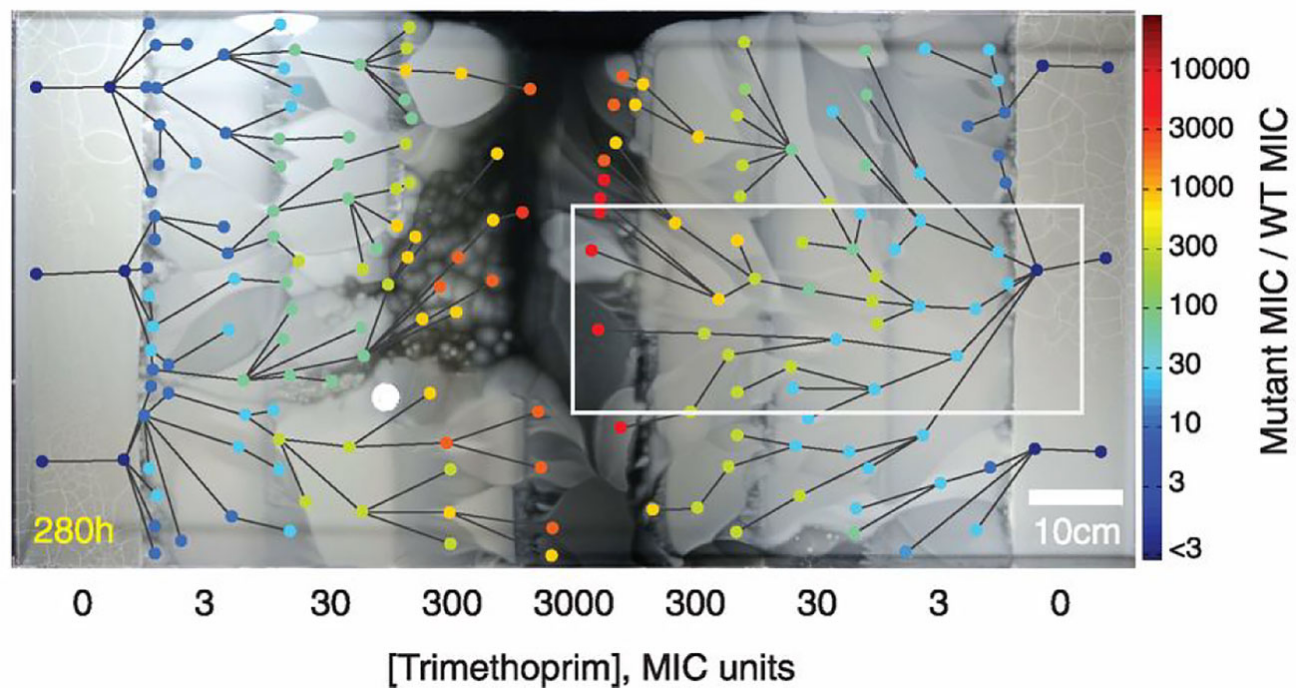
Evolution of antimicrobial resistance in the *E. coli* mega-plate experiment. In a pivotal evolutionary experiment, Baym et al. (2016) plated *E. coli* sensitive to the antibiotic trimethoprim (TMP) on a large (120 by 60 cm) megaplate. Towards the center of this plate, TMP concentrations increase. After 288 hours, *E. coli* populations (appearing as white on a black background) have colonized nearly all of the plate due to the accumulation of resistance mutations. 182 sampled points of clones are indicated by circles, colored by their level of resistance (minimum inhibitory concentration). Lines connecting the dots indicate video-inferred lines of descent, yielding a branching pattern tracking the evolutionary events. Reproduced from Baym et al., Science 353(6304):1147–1151 (2016). © AAAS. Reprinted with permission.

While nutrient competition is a cornerstone of microbial ecology, especially in resource-limited environments (Foster and Bell 2012, Estrela et al. 2022), the spatial arrangement of cells and resources can in many cases become the dominant factor shaping ecological and evolutionary outcomes. Spatial confinement or colonization of new territory often leads to highly stochastic community assembly, with markedly different trajectories depending on the spatial arrangement of founding cells (Lloyd and Allen 2015, Eigentler et al. 2022). Spatial constraints can drastically influence evolution, even under strong selection. For example, using an antibiotic gradient, the pivotal “mega plate experiment” (Baym et al. 2016) showed how resistant strains emerge and spread from initial access points (Figure 4). Cell motility adds another layer to this picture: chemotaxis, gliding, and collective swarming can reorganize spatial structure and reshape ecological interactions. Considering that 40–80% of microbes on Earth live in densely packed communities (Flemming *et al*. 2019), the interplay of growth, movement, and spatial constraints has far-reaching consequences for global microbial ecology and evolution.

These densely-packed communities can be broadly grouped into two categories: aggregates suspended in liquid and surface-attached biofilms (Flemming and Wuertz 2019, Cai 2020). Especially in biofilms, cells are often embedded in an extracellular matrix composed of secreted polysaccharides, proteins, and DNA. The matrix supports the development of 3D spatial structure, and along with external factors such as fluid flow, mediates biophysical interactions between cells and their surroundings, while chemical gradients arising from metabolic activity and cell-to-cell signaling further shape spatial organization (Drescher et al. 2016, Nadell et al. 2016, Evans et al. 2023). The following subsections examine how aggregate and biofilm architectures emerge and how they influence microbial ecology and evolution.

### Self-assembly of 3D spatial structures

#### Microbial aggregates in liquid

Microbes very often clump together in liquid environments, and these aggregates exhibit diverse morphologies, ranging from diffuse, cloud-like structures to dense, compact formations. Diffuse aggregates can be mediated by extracellular DNA (eDNA), which forms flexible loops or strands that loosely connect cells, resulting in an open structure that facilitates fluid infiltration and nutrient exchange (Whitchurch et al. 2002, Das et al. 2011, Tavaddod et al. 2024). In contrast, the formation of dense aggregates can be mediated by exopolysaccharides (EPS), which act as adhesive scaffolds, or by depletion interactions, where EPS are excluded from the narrow spaces between cells, creating an entropic force that pulls cells closer together (Dorken et al. 2012, Secor et al. 2018). These compact structures can protect cells from environmental stress and may also facilitate the establishment of chemical gradients. Aggregate morphology is dynamic and can shift between different forms depending on environmental conditions and matrix composition (Nadell et al. 2016, Flemming and Wuertz 2019, Melaugh et al. 2023). An interesting example is provided by alginate-degrading *Vibrio splendidus* strains. Strains with both weak and strong degradation abilities form aggregates in liquid. However, as the aggregates grow beyond 15-20 µm, diffusion limitations intensify, and different strains adopt distinct strategies to maintain access to alginate. The weak degrader compensates by forming dense aggregates and initiating internal mixing through circular cell movement, homogenizing gradients, and drawing in additional substrate. In contrast, the strong degrader forms loosely packed aggregates and develops fluid channels that enhance alginate flow into the aggregate interior (Ebrahimi et al. 2019b).

Moving beyond bacteria, a complementary system that highlights how spatial organization can generate multicellular properties is snowflake yeast. These clonal clusters of *Saccharomyces cerevisiae* originate from selection for rapid settling or from mutations in *ACE2*, which prevent daughter-cell separation (Ratcliff et al. 2012, 2015). Snowflake yeast develop three-dimensional architectures, internal gradients, and group-level reproduction by fracturing, mirroring features observed in microbial aggregates. Long-term evolution experiments show that snowflake yeast can reach millimeter-scale, mechanically integrated multicellular bodies (Bozdag et al. 2023) and maintain rapid growth at macroscopic sizes by generating metabolically driven fluid flows that circulate nutrients into their interior (Narayanasamy et al. 2025). Their physiology allows the study evolutionary innovations, for instance by heterologous expression of oxygen-binding proteins such as myoglobin or myohemerythrin (Wong et al. 2025). Snowflake yeast is a powerful experimental system for linking unicellular behaviors, spatial structure, and the emergence of macroscopic multicellularity, and illustrates how many multicellular traits that arise in aggregates can be driven and stabilized by spatial organization.

#### Surface-attached biofilms

Microbial communities also often form surface-attached biofilms, in which spatial organization is further influenced by interactions with solid substrates. In biofilms, ecological dynamics are driven by an interplay between nutrient and signal gradients, flow conditions, and physical interactions, including with the extracellular matrix, as well as spatial constraints imposed by the surface and initial colonization patterns—this can result in a range of morphologies. In flow chamber experiments, biofilm structures formed by *Pseudomonas aeruginosa* range from smooth, flat films to mushroom-like colonies, depending on nutrient input, shear forces, and matrix production (Pamp et al. 2009). A further key determinant of biofilm structure is the initial spatial arrangement of cells on the surface. For example, when cells attach in clumps, they can gain early access to nutrients and outcompete others, triggering the emergence of rough biofilms with protruding fronts (Kragh et al. 2016, Melaugh et al. 2016).

As a biofilm grows, nutrients are consumed faster than they diffuse inward, giving rise to a metabolically active surface layer (known as the active layer) and a deeper inactive zone (Dockery and Klapper 2001). The thickness and dynamics of the active layer are central in controlling biofilm structure. Under rich nutrient conditions, a thick, continuous active layer results in a smooth, flat biofilm. In contrast, under nutrient-limited conditions, slight surface elevations result in increased nutrient uptake at the peaks and starvation in the valleys; in turn, the peaks grow faster than the valleys, producing a positive feedback leading to biofilm fingering. This feedback sharpens surface roughness and creates discontinuous growth zones (Dockery and Klapper 2001). Dynamical fluctuations of the active layer also play a role in the transition to biofilm fingering (Young et al. 2023). These differences in surface morphology have ecological and evolutionary consequences: rough, patchy biofilms tend to promote strong genetic drift by isolating lineages within spatial niches; this reduces diversity due to stochastic extinction of lineages, while new clones that arise during growth tend to be spatially localized. Smooth biofilms with thicker active layers, by contrast, support greater spatial mixing among lineages and tend to retain more genetic diversity (Drescher et al. 2016, Young and Allen 2022).

### Colonies on an agar plate

In the lab, microbes are often grown as colonies on semi-solid media in agar plates. These colonies typically exhibit dense packing, with cells competing for space by physically pushing against each other as they grow. Such colonies can also exhibit complex spatial organization. For example, in monoclonal *E. coli* colonies, the steep gradients in resource availability arise, resulting in at least two distinct metabolic phenotypes (Díaz-Pascual et al. 2021). Close to the agar, the colony is anoxic. Here, cells synthesize and secrete alanine, which serves as a carbon and nitrogen source for cells growing in the oxic zones further from the substrate. A similar spatially separated intraspecies cross-feeding interaction has been observed in anoxic pockets where glucose is fermented to short-chain fatty acids, like acetate or lactate, which are then respired in the oxic layer (Cole et al. 2015, Wolfsberg et al. 2018). This phenomenon also appears in microfluidic devices (Dal Co et al. 2019a,b).

The organized spatial arrangement of cells in densely packed communities can also give rise to other emergent physical properties such as structural color. Some dense microbial communities exhibit iridescence, a structural coloration produced through coherent reflection of specific wavelengths (Johansen et al. 2018, Hamidjaja et al. 2020). Mechanistically, this results from the precise, periodic packing of rod-shaped cells, often rapidly assembled through gliding motility, which creates a living photonic crystal. This optical phenotype serves as a direct macroscopic readout of microscale spatial organization because it emerges only when cells align with submicron periodicity. The reflected color is highly sensitive to this spacing; tighter packing shifts the reflected wavelength toward the red end of the spectrum. Iridescent biofilms are predicted to be common at air–water interfaces and on polysaccharide-rich particles in aquatic environments, including marine snow (Box 1). Although the ecological relevance of structural color remains debated, several lines of evidence suggest important functional roles. The phenotype is genomically associated with pathways for polysaccharide metabolism (Zomer et al. 2024), implying that ordered packing may facilitate efficient extracellular polymer degradation by retaining secreted enzymes and reducing diffusional losses during particle sinking (Alcolombri et al. 2021, Chajwa et al. 2024). Notably, the discovery of structural-color genes in deep-sea bacteria where light is absent indicates that the underlying physical structure likely provides non-optical benefits, such as enhanced nutrient acquisition or protection from viral infection, or that the coloration is simply a visible byproduct of organizational strategies that optimize gliding or packing.

### Spatial growth dynamics

**Figure 5.**
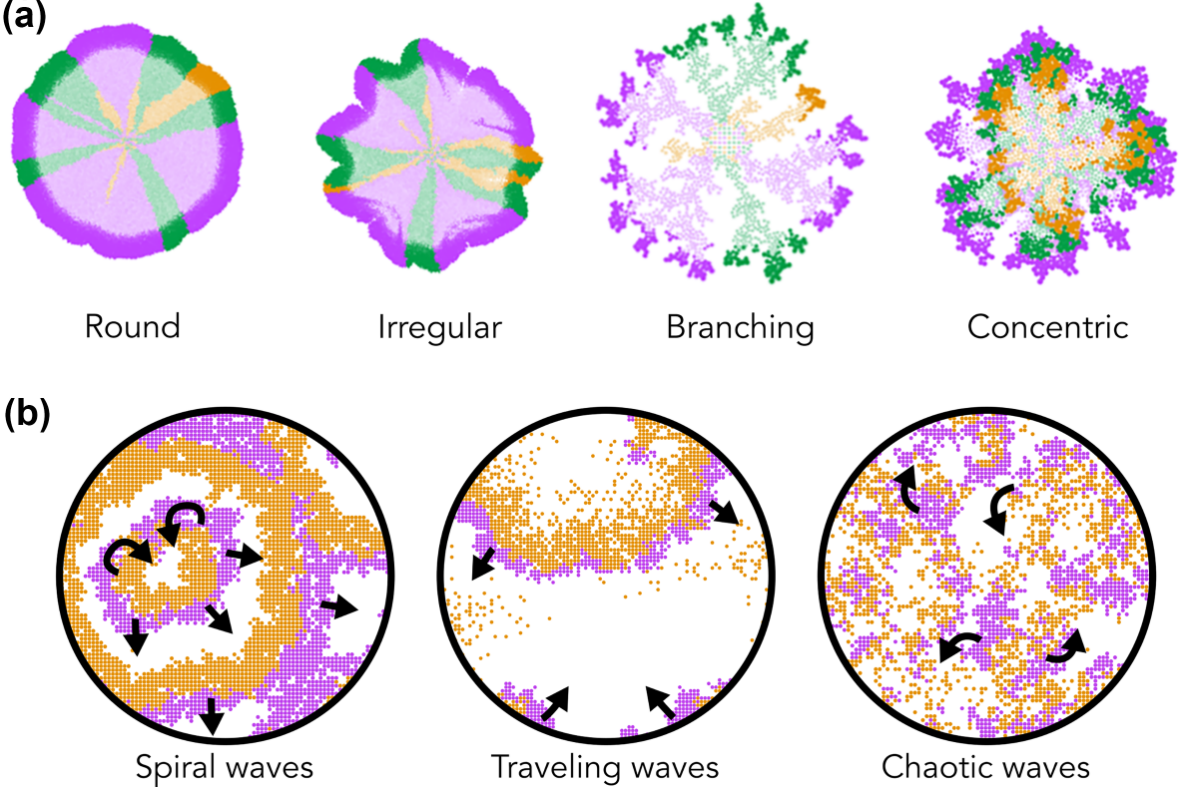
Structure emerges in Individual-based modeling. (a) Ecological and environmental factors explain the complex colony morphology. Image adapted from Martinez-Rabert et al. (2022), with permission. (b) Various waves produced in a simple ecological model of patches, where purple patches can grow into available space (white), and orange patches can displace purple patches, resulting in a variety of wave-patterns dependent on the growth rates (Figure generated with Cacatoo (van Dijk 2022), interactive version available at https://bramvandijk88.github.io/cacatoo/ecology_in_space/).

As microbial colonies expand on the agar surface, they form a wide variety of shapes, sizes, and patterns, ranging from smooth and circular to rough, sectored, or highly branched morphologies. Recent work shows that combining simple rules of cell mechanics with metabolic interactions can already reproduce many of these complex colony morphologies in silico (Dukovski et al. 2025). Many more factors influence colony growth dynamics, including nutrient availability and substrate stiffness (Schwarcz et al. 2016, Bottura et al. 2022), diffusion limitations (Tronnolone et al. 2018), cell motility (Li et al. 2021), mechanical interactions (Delarue et al. 2016, Rani and Sengupta 2024), and ecological interactions (Ruan et al. 2022, 2024). For example, intra-colony channels observed in *E. coli* biofilms (Bottura et al. 2022), thought to be adaptive in the face of spatial restrictions, are shaped by nutrient concentration (Schwarcz et al. 2016, Bottura et al. 2022). Indeed, models that account for different aspects of nutrient- and ecological dynamics can produce a wide variety of emergent colony morphologies (Blanchard and Lu 2015, Gralka and Hallatschek 2019, Martinez-Rabert et al. 2022) (Figure 5a).

While colony morphology reflects a scenario where microbes grow rapidly under constant, resource-rich conditions, dynamics can be very different in natural environments where, for example, nutrients likely undergo cycles of depletion and renewal. Moreover, communities in the natural environment may become structured over much longer eco-evolutionary timescales.

Although the spatial patterns that emerge over extended timescales are difficult to capture experimentally, spatially explicit ecological and evolutionary models consistently produce wave-like dynamics, ranging from orderly spirals and traveling waves to chaotic fronts (Figure 5b). These patterns may influence diverse ecological mechanisms, including cooperation (Blanchard and Lu 2015, Colizzi and Hogeweg 2016a), bet-hedging (Rulands et al. 2014, Lowery et al. 2017), antagonism (Pagie and Hogeweg 1999, 2000, van Dijk and Hogeweg 2015, 2020, Booth et al. 2023), and division of labour (Colizzi et al. 2023). Interestingly, recent experiments show that even single-species systems exhibit complex spatio-temporal patterns (Werner and Arndt 2025).

Why are dynamical patterns important in the context of spatial microbial systems? In well-mixed systems, dynamical instabilities often lead to irreversible collapse. Once cheaters outcompete cooperators or a costly trait is lost, the population typically converges on the more selfish or cheaper strategy. Spatial structure changes this outcome. Local collapses can be counterbalanced by recolonization from neighboring patches, allowing cooperative or otherwise advantageous traits to persist.

Cyclic dominance (rock–paper–scissors dynamics) illustrates this principle well (Figure 1b). In structured environments, cyclic interactions prevent any single strain from monopolizing the system, maintaining diversity through repeated patch turnover (Kerr et al. 2002, Schreiber and Killingback 2013, Gude et al. 2020). One of the most well-studied systems demonstrating this dynamic is colicin-mediated interference in *Escherichia coli*. Colicins are protein toxins released by producing cells to kill susceptible competitors, often at a cost to the producer due to autolysis or the energetic burden of toxin synthesis. In natural *E. coli* populations, colicin-producing strains coexist with colicin-sensitive and colicin-resistant strains, forming a nontransitive competitive loop: producers kill sensitives, sensitives outgrow resistants (which bear a fitness cost for resistance), and resistants outcompete producers. These interactions are profoundly shaped by spatial structure. In well-mixed environments, toxin producers rarely thrive at low densities as the benefits of killing are shared with non-producers, and the cost of toxin production puts them at a disadvantage. However, in structured environments like agar plates or biofilms, local dispersal and limited diffusion allow producers to create zones of high toxin concentration, eliminating nearby sensitive competitors and gaining exclusive access to the vacated space (Chao and Levin 1981, Kerr et al. 2002, Kirkup and Riley 2004). This spatial localization of benefits enables producers to invade from low frequency and stabilize coexistence within spatial mosaics. Spatially explicit simulations further support that such local interference dynamics can robustly maintain high strain diversity through cyclic dominance and self-organizing patch structures, even in the absence of external heterogeneity (Pagie and Hogeweg 1999). These dynamics have also been demonstrated in microbial systems via antibiotic interactions (Kerr et al. 2002, Schreiber and Killingback 2013, Doekes et al. 2019) and also apply to antagonistic mechanisms like the T6SS (Borenstein et al. 2015, Stubbusch et al. 2025). Here, bacteria kill neighbors and exploit the vacated space, while strains with different susceptibilities and resistances coexist, generating complex spatial mosaics (Smith et al. 2020a,b). These mosaics allow traits that would be purged in well-mixed populations to persist at the metapopulation level. Multi-level models predict similarly counterintuitive outcomes, where opposing selection pressures favor traits unlikely to emerge otherwise (van Dijk et al. 2020, 2022, Hermsen 2022). A central challenge is to test how such dynamical patterns, so robust in computational models, shape the ecological and evolutionary trajectories of natural microbial communities.

### Founder effects

Spatial effects are also important for understanding how new microbial populations initiate. Here, genetic drift is highly relevant and can be beneficial, since the few cells that found a new population on a particle can, by chance, fix mutations that would otherwise be selected against, so-called **founder effects**. However, new populations also face hurdles: for instance, microbes can exhibit a phenomenon known as the Allee effect (Allee and Bowen 1932): a requirement for a minimum cell density for some functions that may result in density-dependent fitness, hindering a single cell from founding a new population. Modeling studies indicate that spatial structure may mitigate these Allee effects, as similar ecotypes tend to group together and promote the spread and maintenance of cooperative traits (Colizzi and Hogeweg 2016a, Meijer et al. 2020). Once the initial hurdles of colonization are overcome and populations expand spatially via **range expansion**, genetic drift is again important due to stochastic fluctuations in growth dynamics at the expanding front (Excoffier and Ray 2008, Fusco et al. 2016).

### Range expansion

#### Allele surfing

In expanding colonies, only the cells at the edge have access to sufficient nutrients and space to proliferate. This confines the competition between parental strains and fitter mutants to the expanding interface of the population, thereby reducing the strength of selection. Pre-existing mutants (known as standing variation) (Gralka et al. 2016) or *de novo* mutations occurring early in the expansion, termed **mutational jackpot** events, can form large monoclonal sectors as the population expands (Table 2) (Fusco et al. 2016, Hallatschek 2018). Although selection for advantageous mutations remains effective, this process allows neutral and even deleterious alleles to reach high frequencies (Edmonds et al. 2004, Klopfstein et al. 2006, Peischl et al. 2013), a phenomenon known as allele surfing. As a consequence of allele surfing, tenfold more mutations have been found to reach high frequency during range expansion than during uniform growth (Fusco et al. 2016). Although diversity within the population declines as it expands, due to surfing, *successive* range expansion effects, e.g. when one strain facilitates the growth of another, can promote diversity by breaking up monoclonal sectors and promoting intermixing (Goldschmidt et al. 2017, Meijer et al. 2020, Liu et al. 2021). Genetic drift and diversity loss are further modified by dispersal and Allee effects (Birzu et al. 2019). The Allee effect can reduce genetic drift, as it increases the effective population size by shifting growth away from the population edge. Slowing down the expansion via toxins (Goldschmidt et al. 2018) or phage predation at the front also reduces demixing and drift (Ruan et al. 2024). Dispersal from the non-growing core to the expanding front may also increase effective population size and reduce drift (Birzu et al. 2019), for example, if cells can travel along fungal hyphae (Ruan et al. 2022). Larger population sizes, e.g. enforced by social motility (Li et al. 2021), might also decrease genetic drift. In summary, the concept of effective population size, which reflects how much genetic variation a population can maintain and how strongly selection can act, is strongly influenced by spatial structure. Spatial growth can increase genetic diversity, thereby expanding the pool of variation available to natural selection. At the same time, the inherently local nature of competition in structured environments can reduce the efficiency with which this variation is filtered by selection.

#### Spread of antibiotic resistance in a spatial context

Surface-attached biofilms are well-established hotspots for horizontal gene transfer, including the conjugative spread of plasmids carrying antibiotic resistance genes (Savage et al. 2013, Abe et al. 2020). However, during range expansion, plasmid conjugation is impeded by demixing, i.e. clonal segregation in space, which reduces the interface between interacting strains (Ma et al. 2023). Interestingly, microbial interactions within the expanding populations, such as phage predation (Ruan et al. 2024) and cross-feeding (Ma et al. 2024), can counteract this segregation and maintain opportunities for plasmid transfer. In obligate cross-feeding communities, mutual dependence maintains mixed populations (Müller et al. 2014) while in unidirectional interactions, such as nitrite cross-feeding, consumer strains are pulled along by producer lineages during colony expansion (Goldschmidt et al. 2017, 2021).

The spatial arrangement that emerges during range expansion determines which members of a microbial community are more likely to receive mobile genetic elements such as conjugative plasmids. Since the growth potential of a newly formed transconjugant depends on its spatial position, cells at the expansion front have a higher chance of being the receiver of the plasmid. For example, in nitrite cross-feeding communities, nitrate-reducing producers lead the expansion, while nitrite consumers follow. Consequently, conjugation events involving frontier nitrate producers were found to be more likely to succeed and amplify resistance gene spread (Ma et al. 2024).

Moving beyond gene transfer, spatial structure can also lead to local protection of susceptible strains. For example, some resistant cells can degrade antibiotics locally, creating protective niches that allow the growth of satellite colonies, i.e. sensitive cells surviving in the vicinity of the producer despite external antibiotic pressure. This effect is well documented for β-lactam antibiotics, which are hydrolyzed by β-lactamases produced by resistant cells and in diderms, typically retained in the periplasm, or released via vesicles (Wang et al. 2025b, Medaney et al. 2016). Importantly, the spatial range of this protection can expand over time as resistant populations grow and spread. For instance, in cocultures of *Enterococcus faecalis* grown on agar, β-lactamase-mediated protection was initially confined to the immediate vicinity of resistant cells, acting over only a few micrometers during the first ∼18 h of incubation (Denk-Lobnig and Wood 2025). In contrast, when the same strains were inoculated up to 2 cm apart, sensitive populations were able to grow after ∼36 h, indicating the emergence of long-range protection (Sharma and Wood 2021). In this regime, resistant colonies act as sinks for the antibiotic, continuously degrading diffusing antibiotic molecules and establishing large-scale concentration gradients that shield neighboring cells. These effects have also been observed in multispecies settings, where a β-lactamase-producing strain can protect other community members, though the benefit is highly context-dependent and varies with the antibiotic’s degradation kinetics and the inherent tolerance of the recipient species (Frost et al. 2018). Notably, modeling work and experimental data show that spatial structure affects this cross-protection dynamic: when resistant and susceptible populations remain spatially associated (mixed), protection is maintained, but when they demix, as often occurs during range expansion, susceptible cells are no longer shielded and may be eliminated (Pathak et al. 2023). Moreover, the strength of selection for resistance itself depends on the spatial context. In *Pseudomonas* surface colonies, β-lactamase-mediated protection can be so efficient that susceptible strains outperform resistant ones at intermediate antibiotic concentrations, effectively weakening the selection for resistance (Medaney et al. 2016).

Spatial structure can also slow down the spread of antimicrobial resistance. In the absence of antibiotics, fast-growing sensitive lineages will dominate the expansion front, while slower-growing resistant mutants are confined to interior regions of the colony. When subsequently selecting for antibiotic resistance, the resistant strains may only experience fitness advantages after the sensitive strains have been eliminated (Fusco et al. 2016, Gralka et al. 2016). The MEGA-plate experiment (Figure 4), where *E. coli* expanded across a 1.2-meter gradient of antibiotics, exemplifies this spatial filtering. Early resistance mutations enabled survival at low antibiotic concentrations but later fell behind. Despite subsequently acquiring compensatory mutations and overall higher fitness, these resistant clones remained trapped behind the advancing front, unable to rejoin the leading edge due to spatial constraints (Baym et al. 2016).

#### Range expansion in heterogeneous landscapes

When cells expand into heterogeneous landscapes, such as soil pores or gut villi, expanding clonal sectors can be stalled by obstacles and/or buried by co-expanding fronts (Borer et al. 2020), regardless of fitness differences. The evolutionary trajectory of expanding populations can then decouple from selection and may depend mainly on the obstacle density, the make-up of the physical space, and the overall mutation frequencies (Bosshard et al. 2017, Gralka and Hallatschek 2019). At intermediate obstacle densities and for realistic mutation parameters, genetic drift can theoretically cause a *mutational meltdown*, a mechanism wherein the accumulation of deleterious mutations leads to a self-reinforcing decline in population size and local extinction (Hallatschek and Nelson 2010, Gralka and Hallatschek 2019, Zhang et al. 2022).

### Motility

Beyond expansion via growth, many microbes can move on their own or in groups, shaping ecological interactions and evolutionary dynamics in spatially structured environments. In particular, chemotaxis, where organisms move in response to chemical gradients, enables microbes to navigate toward favorable conditions or away from harmful environments (Raina et al. 2022). This form of directed movement is especially relevant in heterogeneous habitats where nutrient gradients are prevalent and nutrients quickly depleted (Stocker et al. 2008). Chemotaxis significantly impacts community structure and function, promoting localized adaptation and interaction within the community (Cremer et al. 2019, Keegstra et al. 2022, Seymour et al. 2024). For example, heterotrophic bacteria in the oceans use chemotaxis to locate picocyanobacteria and increase their otherwise scarce uptake of heterotrophic carbon and nitrogen (Raina et al. 2023). Similarly, in infection, chemotaxis guides *P. aeruginosa* cells toward host tissues, where surface contact triggers stable attachment. Here, asymmetric cell division then results in one surface-attached cell and one motile daughter cell equipped with flagella, enabling efficient propagation of the infection (Laventie et al. 2019), although the regulatory mechanisms are not completely understood (Dar et al. 2021). Chemotaxis can also drive microbe-microbe interactions, for example, *P. aeruginosa* strains chemotactically sense *Staphylococcus aureus* microcolonies, physically invade them, and kill them from the inside out (Limoli et al. 2019, Sánchez-Peña et al. 2024). Chemotactic motility can even facilitate new eco-evolutionary mechanisms, for example, in collectively migrating populations, a form of non-genetic inheritance can arise when individuals with less well-adapted chemotaxis ability fail to keep up with the migrating population (Mattingly and Emonet 2022).

Swarming is another important form of microbial mobility. Swarming involves collective movement across surfaces, allowing microbes, for example *Bacillus subtilis*, to explore and colonize new niches (Jeckel et al. 2023). Interestingly, swarming has been linked to “cooperation across generations”, in which earlier generations deposit metabolites that can be consumed by later generations that cross the same path (Jeckel et al. 2023). Swarming is also linked with kin selection and thus genetic spatial structuring, since closely related strains tend to swarm together while more distantly related strains do not (Kraigher et al. 2022). In general, the combination of spatial structure and microbial motility can drive species coexistence by enabling the exploitation of diverse niches and minimizing direct competition (Gude et al. 2020).

To summarize, spatial constraints and local interactions shape microbial ecology and evolution in diverse settings from colonies to biofilms and aggregates. As populations expand, nutrient gradients, mechanical forces, and limited mixing through motility combine to alter competition and selection. Spatial structure can allow neutral and deleterious mutations to rise in frequency, facilitate the emergence of metabolic dependencies, and influence the spread of mobile genetic elements. Motility adds further complexity by redistributing cells across the landscape and potentially linking otherwise isolated, fragmented populations, as we discuss in the next chapter.

## Metapopulations

### Microbial islands and habitat fragmentation

Natural microbial populations are often fragmented, i.e. distributed across diverse microhabitats or sites, forming multiple relatively isolated subpopulations. This is analogous to the macroecological concept of island ecology. Examples include soil pores (Carson et al. 2010, Totsche et al. 2018, Bickel and Or 2023, Li et al. 2024), polysaccharide particles (Nguyen et al. 2022), villi and crypts in the gut (McCallum and Tropini 2024), and skin pores (Conwill et al. 2022). We highlight polysaccharide particles in more detail in Box 1, because they offer a well-studied system in which many of the processes discussed throughout this review, i.e. colonization, succession, niche construction, and eco-evolutionary feedbacks, play out within a single discrete habitat, making them an instructive model for the metapopulation concepts explored in this section.

In soil, most pores harbor very low numbers of microbial cells (ranging from 100 to 1000), and these subpopulations are founded by far fewer cells (Bickel and Or 2023). In hair follicles, population sizes are larger; for instance, the anaerobic *Cutibacterium acnes* range between 10^4^ and 10^6^ cells per follicle (Claesen et al. 2020), with an average of 50 000 cells (Conwill et al. 2022). In skin pores, however, the number of cells that have access to nutrients and are protected from oxygen is much smaller due to the physical structure of the pores (Conwill et al. 2022). Similar spatial constraints may also limit the number of actively growing cells in other biomes where habitats exist as fragments or islands. Even habitats that appear continuous, such as large microbial mats or biofilms, are internally structured by fine-scale heterogeneity in nutrients, oxygen, and physical architecture. These gradients create pockets of relative isolation that shape local interactions, constrain dispersal, and influence how lineages spread or evolve across the community. Models that treat space as a connected continuum should therefore still account for the effective separation of microhabitats imposed by this heterogeneity. Physical and spatial constraints jointly determine how organisms are distributed across microhabitats and how ecological and evolutionary processes unfold within and between them.

#### Ecological and evolutionary consequences of habitat fragmentation

**Figure 6.**
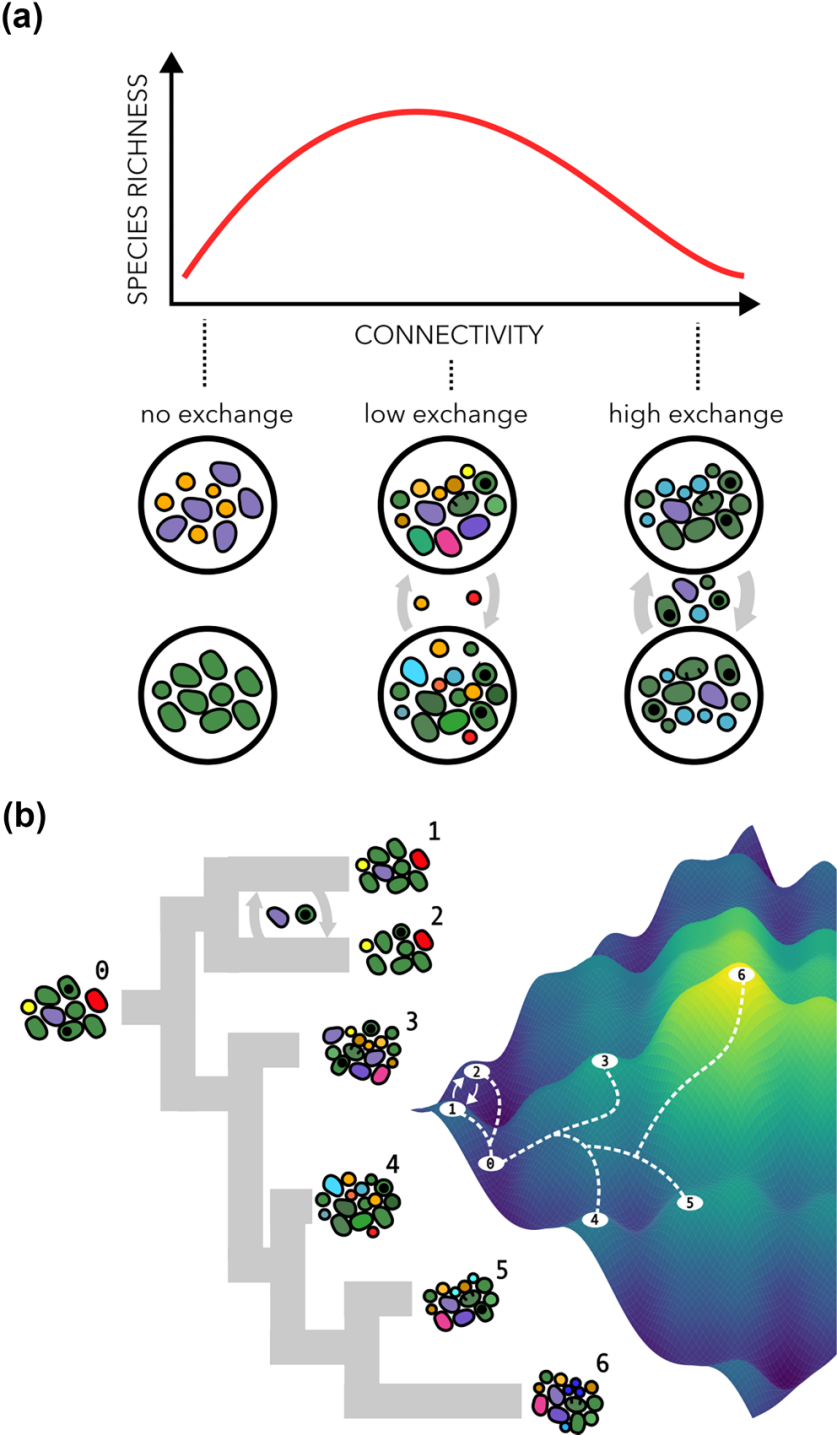
Fragmentation of populations affects diversity and evolvability. (a) Connectivity of spatially separated metapopulations shapes their diversity, with intermediate connectivity yielding the highest species richness (Carson et al. 2010, Bickel and Or 2020). (b) Metapopulation diversity can enhance evolutionary exploration through a parallel search within genotype space. Too much mixing (or too little) may therefore decrease the chance of deviating from local peaks, as illustrated by the top two populations. The other populations are spatially segregated, allowing a parallel search where one of the populations eventually finds the global fitness optimum.

In macro-ecology, habitat fragmentation can either enhance or reduce diversity (Fahrig 2017), with the degree of connectivity between subpopulations being an important driver of metacommunity diversity (Figure 6a) (Leibold et al. 2004). For microbial communities, metagenomic studies suggest that intermediate connectivity yields higher diversity than either complete isolation or free migration between microbial populations (Carson et al. 2010, Bickel and Or 2020). The effects of habitat fragmentation have often been viewed from the perspective of cooperative traits. Fragmented habitats can stabilize cross-feeding by shielding cooperators from cheaters (Nowak and May 1992, Momeni et al. 2013, Lobanov et al. 2023), whereas traits such as mobility have lower fitness benefits in fragmented habitats and may be disfavored (Luo et al. 2024). Other studies have found that spatial structure can favor cooperation even at high costs (Colizzi and Hogeweg 2016a), although both cooperation and spatial structure can also reduce total community productivity (Oliveira et al. 2014, van Vliet et al. 2022). This touches on the concepts of Simpson’s paradox, in which cooperators can increase in overall frequency in a fragmented metapopulation, even though they are outcompeted by cheaters in each individual subpopulation (Wilson 1975, Chuang et al. 2009, Hermsen 2022).

Small local populations experience strong ecological drift, that is, random compositional changes due to stochastic birth-death fluctuations (Vellend 2010, Siqueira et al. 2020). Droplet microfluidics enables the simultaneous analysis of thousands of small, stochastically seeded subpopulations in the lab (Hsu et al. 2019, Mahler et al. 2021). For example, when a mixture of *Pseudomonas* strains are seeded with only 0–3 cells per strain per droplet, the fitter strain can be locally overgrown, reversing large-scale competitive outcomes (Batsch et al. 2024). Random assortment into small subpopulations is also predicted theoretically to strongly promote survival of cooperative antibiotic-degrading microbes under antibiotic stress, a phenomenon termed fragmentation-rescue (Verdon et al. 2025). Both these effects stem from the strong influence of stochasticity in small populations (Mant et al. 2025). Experiments and modeling further show that spatial partitioning itself modulates which interactions dominate: partitioning promotes persistence under antagonism but suppresses cooperative dependencies, with maximal diversity observed at intermediate partitioning (Wu et al. 2022). Consistent with this, microdroplet experiments using synthetic *Lactococcus* consortia revealed that both cooperation and cheating hinge on co-localization probabilities: cooperators can prevail only at intermediate founder sizes, whereas even modest cooperation-independent growth enables cheaters to dominate (van Tatenhove-Pel et al. 2021a).

Microfluidic droplet experiments mimic some, but not all, aspects of fragmented natural habitats. In soil, microaggregates are founded by a few cells, grow mostly isolated from other aggregates, and periodically reintegrate into the metacommunity when aggregates dissolve, thus allowing for parallel exploration of evolutionary landscapes, potentially fixing novel or rare adaptations (Figure 6b). Indeed, some authors consider microaggregates in soil “massively concurrent evolutionary incubators” (Rillig et al. 2017), since the sheer number of microaggregates enables the testing of numerous evolutionary trajectories. Conversely, the skin pore microbiome is also highly fragmented, but without apparent dispersal and selection mechanisms, the skin microbiome appears to evolve neutrally (Conwill et al. 2022).

From an evolutionary perspective, graph theory provides a helpful way to study the effects of habitat fragmentation. Here, the population structure is represented as a connected network (graph) and depending on the topology of the graph, the selective effects of fitness differences between strains can be either reduced or enhanced (Lieberman et al. 2005, Pavlogiannis et al. 2018). If subpopulations are small, selection is weaker, allowing deleterious or neutral mutations to be retained. This facilitates the exploration of epistatic genotypes via sequential mutational pathways where individual mutations might be detrimental, but their combination enhances fitness (Handel and Rozen 2009). However, for highly adaptive mutations and/or larger subpopulations, selection remains effective, and adaptive mutants can disperse to other subpopulations, where they outcompete competitors and increase overall fitness. This dynamic has been observed in *E. coli* populations growing in engineered landscapes in microfluidic devices, where fragmentation facilitates diversification and adaptation (Keymer et al. 2006, France and Forney 2019).

Habitat fragmentation is also relevant in the context of **eco-evolutionary tunneling**, in which ecological factors facilitate transitions across evolutionary fitness valleys to otherwise inaccessible evolutionarily stable states (Nowak 1990, Kotil and Vetsigian 2018). This is a collective, community-level phenomenon that can occur when ecological and evolutionary timescales coincide (Kotil and Vetsigian 2018). Spatially structured systems promote the coexistence of strains by forming local patches where each strain periodically invades and displaces its competitor, leading to a dynamic but stable equilibrium (Kerr et al. 2002, Doekes et al. 2019). Intermediate levels of migration between communities in local patches boost eco-evolutionary tunneling by effectively raising the mutation rate (Vetsigian 2017, Kotil and Vetsigian 2018). Furthermore, the locally stable communities act as self-replicating units that spread via local nucleation events, enabling access to complex ecologies like autonomous or cross-feeding systems (Meijer et al. 2020).

### Viruses and mobile elements as drivers of spatial evolution

Horizontal gene transfer (HGT) mediated by mobile genetic elements (MGEs) is a central engine of microbial evolution. Beyond enabling adaptation to new niches, HGT can affect spatially structured environments at fine scales. Localized transfer of genes coding for toxins, antimicrobials, or polymer degradation can rapidly modify the microenvironment, shifting selection on neighboring cells (Rankin et al. 2011). Modeling shows that spatial structure can stabilize genes that would otherwise be lost under weak or waning selection pressures (van Dijk 2020, van Dijk et al. 2020). This gene rescue effect can promote diversity (van Dijk and Hogeweg 2015) and helps explain why virulence factors are frequently associated with MGEs (Nazir and van Dijk 2025), a phenomenon observed in bacteriophages and fungal viruses alike (Abedon and LeJeune 2007, Gluck-Thaler et al. 2022, Urquhart et al. 2025). Without spatial structure, many ecologically relevant genes would likely be lost in mixed systems where selection pressures are more homogeneous.

Viruses further shape microbial community dynamics through predation and coevolution. The “Kill the Winner” hypothesis predicts that dominant taxa are preferentially targeted by viruses, preventing competitive exclusion and promoting coexistence (Rodriguez-Valera et al. 2009). While evidence is mixed (Castledine and Buckling 2024), case studies of algal blooms (Flynn et al. 2022) and cholera epidemics (Jensen et al. 2006) support this view. Spatial structure can further amplify these dynamics: in microbial colonies, fast-growing lineages tend to occupy the expanding edge, where increased exposure to phage attack leads to their preferential lysis, allowing slower-growing, less vulnerable lineages to persist behind the growth front (Ruan et al. 2024, 2025). These spatial Kill the Winner effects may help explain the persistence of costly antibiotic resistance genes even in the absence of direct selection for resistance.

Phage–host interactions also diversify under spatial constraints. In well-mixed environments, coevolution typically follows arms-race dynamics, with successive sweeps of resistance and counter-resistance mutations resulting in limited diversity (Borin et al. 2023). In spatially structured systems, interactions are localized, resulting in mosaics of infectivity and resistance that increase diversity (Shaer Tamar and Kishony 2022). On larger scales, phages show local adaptation: Vibrio–phage networks from oyster farms reveal modular infection patterns where phages most effectively infect co-occurring *Vibrio crassostreae* strains (Piel et al. 2022), and in soil, phages are more infective toward local than foreign bacteria even on centimeter scales (Vos et al. 2009). Phage lysis can also reshape colony organization by reducing lineage segregation, thereby increasing cell–cell contact, HGT, and altering evolutionary outcomes (Ruan et al. 2024).

Alongside its beneficial functions, HGT can also spread selfish genetic elements (SGEs), such as lysogenic phages and transposons. These elements exploit their hosts, and their epidemiology is shaped by spatial structure because they rely on the proximity and mixing of hosts (Berngruber et al. 2015, Simmons et al. 2018, Eriksen et al. 2020, van Dijk et al. 2020, 2022). Infection by SGEs can lead to local decline or extinction unless countered by resistance (Jonge et al. 2019, Attrill et al. 2021, Romeyer Dherbey et al. 2023). Many temperate phages regulate their life cycle using density-dependent cues. High host densities often favor lysis, as abundant susceptible hosts increase opportunities for horizontal transmission (Silpe and Bassler 2019, Huisman et al. 2025). Conversely, high density, such as in spatially structured systems, can also indicate rapid host growth, favoring lysogeny by allowing temperate phages to piggyback on host success through vertical transmission, often coupled with superinfection exclusion under the “Piggyback the Winner” hypothesis (Knowles et al. 2016, Silveira and Rohwer 2016, Erez et al. 2017, Tan et al. 2020, Doekes et al. 2021, Aframian et al. 2022). Generally, spatial structure enables resistant and sensitive strains to persist in different areas, allowing for long-term coexistence between SGEs and hosts (van Dijk and Hogeweg 2015, van Dijk et al. 2020). Models incorporating spatial structure further show long-term coevolutionary outcomes, including genome streamlining (van Dijk et al. 2022), source-sink dynamics (Bull et al. 2018), and mutational division of labor (Colizzi and Hogeweg 2014).

Taken together, viruses, MGEs, and SGEs are not passive passengers but active players in a spatially structured evolutionary game. Spatial structure amplifies their impact, sustaining both adaptive innovations and parasitic burdens, and thereby shaping microbial diversity and evolution.

In summary, fragmented habitats create networks of small, partially isolated subpopulations where local drift, limited dispersal, and strong environmental heterogeneity dominate ecological and evolutionary dynamics. These conditions can stabilize cooperative traits, enable rare evolutionary innovations, and maintain diversity through spatial turnover and recolonization. Spatial fragmentation also influences the evolutionary fate of antagonistic interactions, metabolic dependencies, and mobile genetic elements. Importantly, metapopulations do not function in isolation: they contribute to emergent patterns that are observable at ecosystem scales. In the following section, we explore how spatial processes propagate upward to shape macroscale ecosystem properties and biogeochemical outcomes.

## The Macroscale

At the macroscale, the central question is how microscale interactions scale up to shape ecosystem processes. Microbes underpin global carbon, nitrogen, and sulfur cycles (Falkowski et al. 2008), but these fluxes often depend on spatial organization at the micrometer scale. In the ocean, the degradation of sinking particles depends on extracellular enzymes such as chitinases, where the spatial balance between degraders, exploiters, and scavengers governs particle turnover and carbon export (Datta et al. 2016, Alcolombri et al. 2021, Nguyen et al. 2022, Pontrelli et al. 2022). Viruses also modulate biogeochemical cycling by reshaping community composition and releasing necromass in both marine and soil microbiomes (Zimmerman et al. 2020, Sokol et al. 2022). In addition, viral auxiliary metabolic genes can directly alter major ecosystem processes: for example, many oceanic cyanophages encode the proteolysis adaptor NblA, which degrades the host’s light-harvesting complexes and is estimated to reduce global photosynthetic light capture by up to 5% (Nadel et al. 2025). Importantly, these systems are not static: microbial traits evolve continuously, feeding back into ecosystem processes. For example, eco-evolutionary modeling of soils shows that the adaptive evolution of exoenzyme production can strongly alter decomposition rates and carbon stocks (Abs et al. 2020). These cases illustrate that global fluxes hinge on spatial dynamics that are both ecological and evolutionary. Microbial macroecology offers a framework for connecting such microscale processes to diversity and function across landscapes and biomes (Mascarenhas et al. 2020). These perspectives highlight the need for models that explicitly link microscale mechanisms, dispersal, and spatial heterogeneity to ecosystem-level outcomes.

### Connecting models on different spatial scales

Spatial structure divides microbiomes into eco-evolutionary microunits that are connected by dispersal (Carson et al. 2010, Bach et al. 2018, Kotil and Vetsigian 2018, Batsch et al. 2024, Doekes and Hermsen 2024); this picture lies at the heart of the metapopulation models discussed in the previous chapter. While high rates of dispersal in spatially structured models can approximate well-mixed models (van Dijk et al. 2020, Nazir and van Dijk 2025), if dispersal is rare, it becomes harder to connect microscale processes to global outcomes. Because cells can only sense their immediate surroundings, the globally averaged concentration of a quorum-sensing signal cannot predict how many cells activate quorum-regulated functions. Gene expression depends on local cell density, signal gradients, and a nonlinear response curve (Boedicker et al. 2009, Darch et al. 2018, Dal Co et al. 2020, van Gestel et al. 2021). Thus, global averages of signaling molecules may not accurately capture population-level activity; instead, the spatial distribution of signals, cells, and responses must be considered to estimate global output.

The example above shows the importance of addressing both environmental heterogeneity and the non-linear nature of biological mechanisms in eco-evolutionary models, even when the aim is to predict macro-scale phenomena (Melbourne and Chesson 2006, Chesson 2012). In this direction, explicitly multiscale frameworks have been developed that can decompose selection into local components and components at larger scales (Doekes and Hermsen 2024). While these are useful tools for the analysis of spatially-structured simulations in which patterns at the microscale are known, it is challenging to account accurately for local heterogeneity in omics-powered models, because the omics data that these models rely on often lacks spatiotemporal resolution. Biogeochemical models, e.g. for microbial nutrient cycling, would greatly benefit from including spatial and temporal heterogeneity (Lehmann et al. 2020, Smercina et al. 2021, Camenzind et al. 2023), but to predict the impact of spatial structure, models need to be developed from the bottom-up and trained on multidimensional, spatially resolved data. Techniques have been developed for spatially resolved simulation of metabolic interactions (Angeles-Martinez and Hatzimanikatis 2021, Dukovski et al. 2021). However, in general, models that predict metabolic interactions from (meta-) genomic datasets, including those based on genome-scale metabolic models, do not take space into account, limiting their ability to go beyond the provision of broad mechanistic hypotheses (Diener et al. 2020).

### Drivers of microbial biogeography on local and global scales

Microbes and their metabolism are deeply connected to the geosphere, as they are involved in virtually all redox reactions and elemental cycles. At local scales, microbial community assembly is driven by the spatial or temporal abundance of primary resources, such as fallen leaves, decomposing animals, food moving through an animal’s digestive system, or organic particles in rivers and oceans. The first microorganisms to colonize newly available resources may depend on chance (Gralka et al. 2020) as well as on traits such as motility and growth rate potential (Lopez et al. 2023). At the global scale, two major axes separate microbiomes: (i) association with higher hosts, and (ii) divergence along an environmental salinity gradient (Lozupone and Knight 2007, Thompson et al. 2017, von Meijenfeldt et al. 2023). The first axis represents a large difference in microbial density and diversity. For example, soils have low microbial density and high alpha diversity, but relatively low beta diversity between sites (Fierer and Jackson 2006, Delgado-Baquerizo et al. 2018, von Meijenfeldt et al. 2023). In contrast, the gut microbiomes of higher organisms contain very dense communities, dominated by relatively few lineages, and thus show lower alpha diversity but higher inter-individual beta diversity (Huttenhower et al. 2012, Falony et al. 2016). There are several reasons for this contrast. First, host-associated systems provide a continuous supply of energy-rich substrates, providing strong selection for fast growing organisms, whose competitive exclusion causes the community composition to diverge (Lopez et al. 2023, von Meijenfeldt et al. 2023). Second, host-associated microbes experience strong selection by host physiology and immune filtering, leading to reduced within-sample diversity and high inter-individual variability. In soils, the density is much lower, and heterogeneous availability of relatively low-energy organic substrates allows many different microbes to coexist (Allison and Martiny 2008, Zelezniak et al. 2015). Thus, the first axis of global microbial biogeography reflects a gradient of resource predictability and host association (Lozupone and Knight 2007, von Meijenfeldt et al. 2023).

While microbiomes associated with higher organisms are divergent from free-living microbiomes, microbiomes associated with invertebrates and plants tend to be more similar in composition to their surroundings. For example, microbiomes associated with water plants and macroalgae contain many aquatic taxa, while land plants contain taxa that are also found in other terrestrial habitats. Similarly, the microbiomes of aquatic sediments more closely resemble those of the associated marine or freshwater than of soil. These widespread observations illustrate Lourens Baas Becking’s dictum, “everything is everywhere, but the environment selects” (Baas Becking 1934, De Wit and Bouvier 2006, Baas Becking and Canfield 2015). All spatial systems are dispersion-limited, but microbes can be transported on a global scale through air, water, animal, and anthropogenic vectors. Upon dispersal, many factors contribute to selection. The chemical composition of the environment differs across systems, contributing to the selection of microbes with different biogeochemical preferences and metabolic traits (Shaffer et al. 2022, Peets et al. 2025). The evolutionary conservation of the associated adaptations reflects the spatial and temporal variability of these factors in the environment. Adaptations to relatively stable environmental factors such as pH and salinity are deeply conserved within broad taxonomic lineages, while adaptations to variable factors such as temperature and drought are more recent (Martiny et al. 2015). Thus, identifying environmental patterns is strongly dependent on the taxonomic resolution at which the microbiome is described. Higher-level taxa such as orders and phyla may best reflect general biome descriptions, but finer-scale habitat structuring may be better reflected at lower taxonomic ranks, such as families or genera (Thompson et al. 2017, von Meijenfeldt et al. 2023). For example, cosmopolitan groups such as the *Pelagibacterales* (SAR11) have divergent ecotypes specialized to distinct ecological and geographic niches (Giovannoni et al. 2014, Delmont et al. 2019).

Large-scale latitudinal diversity gradients exist in both terrestrial and marine microbiomes, with tropical soils supporting higher bacterial richness than high-latitude soils, paralleling plant and animal diversity (Fuhrman et al. 2008, Thompson et al. 2017, Bahram et al. 2018, Delgado-Baquerizo et al. 2018). In the ocean, microbial communities are partitioned in oceanographic provinces defined by temperature, nutrient flux, oxygen, and circulation patterns (Salazar et al. 2016, Bahram et al. 2018, van Vliet et al. 2021). Depth gradients are structured by light and temperature, including surface photic zones dominated by cyanobacteria, copiotrophs, and diverse photoheterotrophs. In addition to chlorophyll-based phototrophy, retinal-binding rhodopsins such as proteorhodopsins are widespread in surface bacterioplankton and can capture amounts of solar energy comparable to, or exceeding, chlorophyll-a-based photosynthesis in oligotrophic waters (Gómez-Consarnau et al. 2019). The deeper aphotic zones are dominated by chemolithoautotrophs such as sulfur-oxidizers, anaerobic ammonium oxidizers, and methanogens (DeLong et al. 2006, Acinas et al. 2021). Finally, soil bacterial diversity often declines with elevation, the dominant lineages shifting with temperature and energy availability along altitudinal gradients (Bryant et al. 2008, Schmidt et al. 2010).

Not all microbes are equally constrained by geography and habitat type. Some lineages act as global generalists, widely distributed across environments. Generalists tend to be fast growers with flexible genomes. This evolutionary flexibility can provide generalist lineages with a broad metabolic repertoire, genes for defense or stress tolerance, and flexible nutrient uptake systems (Martiny et al. 2015, von Meijenfeldt et al. 2023). Other microbes are restricted to particular hosts or habitats. Specialists are genomically more stable, often slow growers that rely on specific functions, depending on their niche. Thus, the biogeography of microbes across the globe is shaped not only by dispersal and the environment, but also by eco-evolutionary strategies of the microbes themselves.

To summarize, macroscale patterns in microbial diversity, function, and biogeochemistry emerge from processes at the micrometer scale. Local interactions, metapopulation structure, and eco-evolutionary feedbacks shape how traits manifest across landscapes and ultimately influence global fluxes. Although large-scale biogeographic gradients and ecosystem processes are often described based on spatially averaged data, they depend on spatial heterogeneity, nonlinear responses, and patch dynamics that originate at much smaller scales. Connecting these levels remains difficult because most models lack explicit spatial resolution. This gap highlights the need for experimental systems and modeling approaches that account for spatial structure, allowing the study of the emergence of microscale mechanisms under controlled conditions. We turn to this topic in the next section on spatial cultivation approaches.

## Researching spatial structure in microbial communities

### Spatial modeling

The previous chapters of this review have shown how strongly spatial structure impacts microbial ecology and evolution. Because space is the natural context in which microbes live and interact, it is crucial to also incorporate it in our mathematical and computational models. Spatially explicit models often yield outcomes that deviate from their well-mixed counterparts, not just in quantitative terms, such as in predictions for growth rates, but also qualitatively, such as whether coexistence is possible at all. Expanding the repertoire of spatially explicit models is thus essential for getting a deeper understanding of the microbial world.

#### Spatial structure in individual-based modeling

While modeling spatial structure in physics is often done with partial differential equations (PDEs), which describe reaction rates in both time and space, these do not fully capture the ecological and evolutionary dynamics discussed in this review. While PDEs can be used to describe how quantities such as cell density and nutrient concentration change smoothly in space and time (Lega and Passot 2003), they do not fully capture the complex heterogeneity within real microbial systems. Because of this, individual-based modeling (IBM) is becoming the gold standard for modeling the intricate, heterogeneous parts of spatial structure in the microbial world (Wang et al. 2025a).

**Figure 7.**
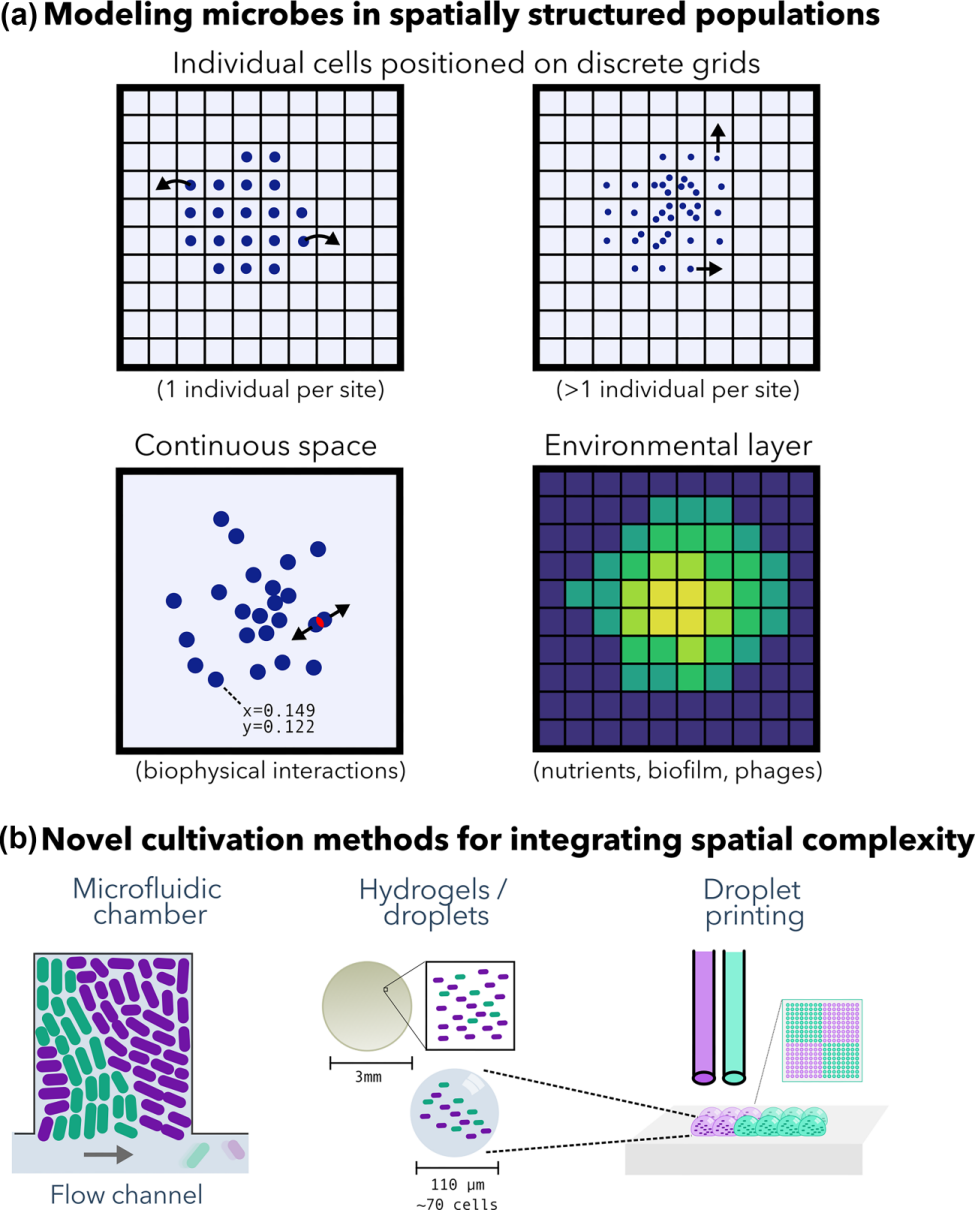
(a) Design choices for modeling microbes in spatially structured populations. Models with discrete grids, where each grid cell can be occupied by one or multiple individuals, are suited for studying how simple rules can create complexity. Models with continuous space may be better at capturing biophysical interactions such as cell shoving, but have a higher computational cost compared to models with discrete grids. In both models, dynamic environmental layers can be included to study how variables like depleting nutrients and accumulating metabolic by-products shape the ecology and evolution. (b) Novel cultivation approaches emerged over the last decade to unpack the impact of spatial structure on microbial ecology and evolution. Microfluidic chambers are micrometer–sized compartments etched in glass or polymer that allow researchers to control nutrient flow and create stable chemical gradients, mimicking the spatial complexity of natural habitats. Hydrogels are composed of highly hydrated polymer networks, which allow for microbial growth. Hydrogels enable the printing of defined microbial arrangements in space *e.g*. by droplet printing.

In grid-based IBMs, individual microbes are positioned on a discrete grid (Figure 7a), interacting only with individuals in neighboring grid cells. Inspired by cellular automata (Hogeweg 1988), the earliest grid-based IBMs allowed at most one individual per grid point (Pagie and Hogeweg 1999, 2000), a simplifying modeling choice that continues to be applied successfully today (Dal Co et al. 2020, Gorter et al. 2020, van Dijk et al. 2020). However, as this choice intensifies competition for “empty space”, additional individuals may be allowed to occupy the same site. In that scenario, additional constraints must be included to prevent unchecked growth, like locally depletable resources. By implementing such constraints, more variability in local cell densities across the grid can arise compared to systems where only one individual is allowed per site. Such local density differences could, for example, be relevant for many of the processes discussed in this review, like quorum sensing, host-phage interactions, and cross-feeding.

Grid-based models have the benefit of being computationally efficient; neighborhoods in these systems are predefined and do not ever need to be recomputed. This allows for large population sizes, which makes them an ideal model formalism to study emergent phenomena. Yet, depending on the biological question, it may be necessary to model space continuously in order to overcome some unrealistic constraints of grid-based models. Continuous representation of space enables biophysical interactions such as mechanical pushing, repulsion, and attraction (Hartmann et al. 2019), which can readily give rise to complex swarming patterns (Reynolds 1987, Vicsek et al. 1995). To the extent that such interactions are relevant for the question at hand, IBMs of microbial systems should also include continuous space (Lardon et al. 2011, Li et al. 2019). Because stable simulations of physics are challenging to implement from scratch, it is advisable to build on existing modeling frameworks and tools that have already solved these issues (Lardon et al. 2011, Li et al. 2019), particularly in cases where capturing physical realism is an important part of the research question. Note, however, that continuous-space simulations are computationally more expensive, which limits them to smaller populations than grid-based models and makes them most suitable for questions where fine-scale physical interactions matter.

IBMs with both discrete and continuous descriptions of space generally use additional, grid-based layers to track environmental variables such as depletable nutrients, biofilms, phages, or other biologically relevant components. These environmental grids then interact dynamically with the population, enabling feedback between microbial behavior and the local environment, which play a central role in, for example, niche construction.

#### Predicting life and exploring basic principles

Models of microbial communities can be used to make quantitative predictions of real-world systems or to explore fundamental principles. **Predictive modeling** using IBM was recently extensively reviewed (Wang et al. 2025a). Great examples include recent efforts that combine deep learning with IBM to predict microbial interactions from self-organized spatiotemporal patterns (Lee et al. 2020). Bottom-up predictive models start from sequencing data to reconstruct metabolic models (Garza and Dutilh 2015, Garza et al. 2022, Bauer et al. 2017, Dukovski et al. 2021) and simulate them in a spatial arena (Bauer et al. 2017, Dukovski et al. 2021). BacArena, for example, revealed the formation of metabolic zones driven by cross-feeding in *Pseudomonas aeruginosa* colonies (Bauer et al. 2017).

IBMs are also an important framework for **exploratory modeling** that aims to explore fundamental eco-evolutionary principles, allowing the inclusion and emergence of spatial patterns (Kerr et al. 2002, Grimm et al. 2005, Hogeweg 2007). For example, a simple computational model combining costly public good production with spatial growth has shown that individuals invested more in public-good production when it was more expensive (Colizzi and Hogeweg 2016a). This finding contrasts sharply with classical game theory, which predicts the extinction of cooperation under analogous conditions (Frey 2010). In spatially structured systems, however, extinctions are not necessarily the endpoint. A local extinction event creates vacant niche space that cooperators can uniquely re-colonize. Another great example of exploratory modeling is the work by Van Gestel and Weissing (2016), who showed that mechanistic gene regulatory networks generate more variable colony responses to nutrient conditions (and morphologies) than simple reaction norms. Long-term evolutionary models have also been used to explore the predictability of evolution (Stephen Jay Gould’s idea of “replaying the tape” of life), complementing insights from experimental studies (Orgogozo 2015, Ratcliff et al. 2015, Blount et al. 2018). For example, the *Virtual Microbes* model (van Dijk et al. 2019, Meijer et al. 2020) integrates spatial structure with genome evolution, metabolism, cell growth, and gene regulation, and shows that the predictability of evolution is strongly influenced by spatial structure: well-mixed populations always followed the same evolutionary trajectory while the outcomes of spatially structured experiments was variable (Meijer et al. 2020).

In summary, while predictive models can aid with practical applications and forecasting real-world ecosystems, exploratory models are useful for challenging assumptions and creating and reshaping intuitions about complex biological systems. It is important to stay cautious about what we think we know about biology, especially when generalizing results from well-mixed systems (both models and experiments) to the complex, spatially structured world in which microbes actually live.

### Spatial cultivation

Shaken, well-mixed cultures are the backbone of many laboratory protocols. Yet, as this review has emphasised throughout, spatial structure plays a crucial role in microbial life at all scales, which limits how well-mixed systems capture spatial ecology and evolution. It follows that experimental setups, including shaken or static liquid cultures, growth on agar plates, or cultivation in microdroplets can only partially grasp the impact of spatial structure in nature. Cultivation approaches often aim to recapitulate nature as much as possible, but full ecological realism is incompatible with complete experimental control. Model systems, therefore, need to strike a balance that preserves key environmental features while remaining tractable. Recent perspectives on synthetic ecosystems and laboratory model habitats have highlighted this balance and demonstrated how controlled synthetic communities can still incorporate ecologically relevant spatial features (Zhalnina et al. 2018, Tecon et al. 2019, Chesneau et al. 2025). Novel cultivation approaches have emerged over the last decade that allow insight into the effects of spatial structure: these include microfluidic devices and hydrogels (Figure 7b).

#### Microfluidics

Microfluidic cultivation methods in microbial ecology (Ugolini et al. 2024) allow the cultivation of microbes in PDMS chambers on small scales closely resembling those of natural environments; they facilitate the emergence of metabolite gradients (Dal Co et al. 2019b, 2020, Díaz-Pascual et al. 2021, Jo et al. 2022, Choudhary et al. 2023, D’Souza et al. 2023), allow time-resolved imaging of individual cells within small communities, and can be designed to resemble porous structures and mimic marine flow environments (Nguyen et al. 2022, Raina et al. 2022, D’Souza et al. 2023), with fine spatial resolution on the scale of a few micrometers (Wu et al. 2023). Droplet microfluidics is a parallel approach that allows the cost-efficient ultra-high-throughput cultivation of microbes in picoliter-sized droplets: this is especially useful for studying founder effects and stochasticity (Mant et al. 2025).

#### Hydrogels

Hydrogels provide a versatile experimental platform for studying spatial structure in microbial communities. They can be formed by various polysaccharides that are compatible with microbial growth (agarose (Krishna Kumar et al. 2021)), polypeptides (mucin (Darch et al. 2018, Jin et al. 2023), gelatin (Connell et al. 2013)), and other polymers (polyethylene glycol-dimethacrylate (Candry et al. 2024)). These three-dimensional, water-retaining polymer networks mimic natural environments by providing a matrix for microbial growth and interactions, bridging the gap between well-mixed liquid cultures and rigid solid substrates. Hydrogels with encapsulated microbes are simple to produce, convenient to image due to their transparency, and allow control of environmental parameters such as nutrient gradients, mechanical properties, and spatial confinement (although with less spatial resolution than with microfluidics), making them a convenient tool for investigating biofilm formation, colonization dynamics, and quorum sensing, among other processes. For example, hydrogel aggregates with encapsulated microbes can be used as soil aggregate surrogates (Candry et al. 2024), while mucin-agar hydrogels are used to study the human gut microbiome (Jin et al. 2023). Similarly, hydrogels have been used to fragment *Pseudomonas aeruginosa* populations by entrapping low numbers of cells to study quorum sensing (Connell et al. 2014).

For more complex applications, shear-thinning, temperature-sensitive, or low-viscosity hydrogels can be 3D printed either through bottom-up assembly (Krishna Kumar et al. 2021, Krishna Kumar and Foster 2022) or top-down etching away of unwanted structures photolithographically (Connell et al. 2013). Advances in bottom-up printing have recently achieved resolutions below the millimeter scale, reaching approximately 110 µm (Krishna Kumar et al. 2021). 3D printing of hydrogels enables, in principle, the arrangement of microbes in any desired spatial configuration, allowing spatial structure to be experimentally manipulated as an independent variable rather than observed as a dependent outcome.

### Spatial omics

Omic techniques are extremely powerful in microbial ecology, but they are generally used for bulk samples, and hence they lack spatial resolution. However, the spatial dimension can be probed by analyzing subsamples, either separately or together via the addition of spatial tags that allow later reconstruction.

#### Spatial (meta-) transcriptomics

In recent years, spatial (meta-) omics sequencing technologies have gained traction for studying eukaryotic organisms (Vandereyken et al. 2023). However, physiological differences between eukaryotes and prokaryotes present challenges for the application of these methods to bacteria. Most spatial transcriptomics techniques consist of chips with 55 µm diameter spots that capture the polyA tail of eukaryotic mRNAs (Moses and Pachter 2022). mRNA, in both bacteria and eukaryotes, makes up roughly 5% of total RNA (Westermann and Vogel 2021). However, bacteria, with their few µm size and absence of polyA tails, present unique challenges (Mohanty and Kushner 2011). Smaller chips with 0.22 µm spot sizes have been used to study eukaryotes and might be suitable for the bacterial size range (Chen et al. 2022) but would require a priori probe design or usage of random probes with additional rRNA depletion steps.

#### Spatial imaging omics

Imaging omics provides an alternative to sequencing approaches. One example is parallel and sequential FISH (par-seqFISH), a spatial transcriptomics technique that has been used, for example, to profile the expression of 105 marker genes in *Pseudomonas aeruginosa* biofilms (Dar et al. 2021). This method revealed age-dependent spatial partitioning of functions such as virulence factor production, denitrification, and fermentation, and even resolved subcellular localization of specific transcripts like *pyocin* mRNA at the cell poles (Dar et al. 2021). In contrast, RAINBOW-seq labels actively growing biofilms in vivo using fluorescent D-alanine conjugates that become irreversibly incorporated into the cell wall. Combined with imaging, machine-learning-guided cell sorting, and low-input RNA sequencing, this approach revealed spatial coordination of alanine transport in *Escherichia coli* biofilms: cells in the nutrient-deprived interior upregulated importers, while peripheral cells expressed exporters (Wang et al. 2023). Similar approaches exploiting the oxygen sensitivity of fluorescent proteins were used to differentially sequence the core and outside of biofilms (Díaz-Pascual et al. 2021).

Highly multiplexed FISH approaches may be used to assess the spatial distribution of taxa. For example, CLASI-FISH (Valm et al. 2012, Mark Welch et al. 2016), HiPR-FISH (Shi et al. 2020), and SEER-FISH (Cao et al. 2023) offer detailed images of the distribution of cells, with the caveat of requiring prior knowledge of the community members for probe design, which could be gathered by sequencing a representative replicate sample. In the absence of universally taggable sequences in bacterial mRNA, universal bacterial and archaeal 16S rRNA and fungal ITS primers may be used to target these microbial groups for taxonomic assessment, combined with standard polyT-probed eukaryotic transcriptomics (Lotstedt et al. 2023, Saarenpaa et al. 2023).

Combining imaging with metabolomics enables a more comprehensive view of microbial spatial organization. Techniques such as MALDI-MSI and Nano-SIMS correlate metabolic activity with spatial structure at micrometer resolution (Geier et al. 2020, Bourceau et al. 2023), while automatic microscopy with robotic sampling can allow further integration of imaging optics (Jeckel et al. 2023). Structural quantification tools such as BiofilmQ extend this by measuring parameters like cell–interface distances and spatial correlation patterns within biofilms (Hartmann et al. 2021). More broadly, spatial structure can be described in diverse ways, for example, by analyzing strain interfaces or periodic autocorrelation patterns. Standardizing such approaches will be key for consistent modeling and cross-system comparisons (Yip et al. 2022, Henderson et al. 2025).

Progress in understanding spatial structure relies on methods that capture microbial life where it actually unfolds: in heterogeneous, locally interacting communities. Spatial models reveal how simple rules generate complex patterns, while microfluidic systems, hydrogels, and emerging spatial omics provide controlled windows into these same processes in real biological systems. These approaches expose mechanisms that are invisible in well-mixed assays and help bridge the gap between theory, laboratory observations and natural ecosystems.

## Conclusion

Spatial structure fundamentally influences how microbes interact, evolve, and persist. From cross-feeding and quorum sensing at micrometer scales to community turnover across fragmented habitats, spatial organization drives patterns that cannot be captured by well-mixed models or bulk metagenomics alone. This review has shown how spatial structure can stabilize cooperation, amplify genetic drift, and enable novel eco-evolutionary pathways. Yet, many challenges remain. First, predictions at the community or ecosystem level are limited by our inability to scale up our modeling studies from individual-level interactions to community-level outcomes. Because spatial structure creates heterogeneity and historical contingency, understanding outcomes requires models that are spatially explicit and grounded in empirical data. Second, while metagenomic sequencing has revolutionized microbial ecology, real-world sampling is often destructive and cannot resolve spatial context. Cultivation approaches that replicate spatial structure are therefore essential for disentangling cause and effect. Future progress will depend on integrating spatial models such as individual-based simulations with emerging experimental tools such as hydrogel-based cultivation and spatial omics. These approaches will help clarify how microbial communities respond to perturbations, adapt to landscape fragmentation, and shape biogeochemical processes. Spatial structure is not merely a complication to be averaged out; it is a key factor in understanding the rules that govern microbial ecosystems. Embracing spatial complexity can help enable quantitative prediction and multiscale mechanistic understanding in microbial ecology.

## References

[bib1] Aaby BH, Desmond H. Niche construction and teleology: organisms as agents and contributors in ecology, development, and evolution. Biol Philos. 2021;36:47. 10.1007/s10539-021-09821-2.

[bib2] Abe K, Nomura N, Suzuki S. Biofilms: hot spots of horizontal gene transfer (HGT) in aquatic environments, with a focus on a new HGT mechanism. FEMS Microbiol Ecol. 2020;96:fiaa031. 10.1093/femsec/fiaa031. 32109282 PMC7189800

[bib3] Abedon ST, LeJeune JT. Why bacteriophage encode exotoxins and other virulence factors. Evol Bioinforma Online. 2007;1:97–110. PMC2658872 19325857

[bib4] Abs E, Leman H, Ferrière R. A multi-scale eco-evolutionary model of cooperation reveals how microbial adaptation influences soil decomposition. Commun Biol. 2020;3:520. 10.1038/s42003-020-01198-4. 32958833 PMC7505970

[bib5] Acinas SG, Sánchez P, Salazar G et al. Deep ocean metagenomes provide insight into the metabolic architecture of bathypelagic microbial communities. Commun Biol. 2021;4:604. 10.1038/s42003-021-02112-2. 34021239 PMC8139981

[bib6] Aframian N, Omer Bendori S, Kabel S et al. Dormant phages communicate via arbitrium to control exit from lysogeny. Nat Microbiol. 2022;7:145–53. 10.1038/s41564-021-01008-5. 34887546

[bib7] Alcolombri U, Peaudecerf FJ, Fernandez VI et al. Sinking enhances the degradation of organic particles by marine bacteria. Nat Geosci. 2021;14:775–80. 10.1038/s41561-021-00817-x.

[bib8] Allee WC, Bowen ES. Studies in animal aggregations: mass protection against colloidal silver among goldfishes. J Exp Zool. 1932;61:185–207. 10.1002/jez.1400610202.

[bib9] Allison SD, Martiny JBH. Resistance, resilience, and redundancy in microbial communities. Proc Natl Acad Sci USA. 2008;105:11512–9. 10.1073/pnas.0801925105. 18695234 PMC2556421

[bib10] Amarnath K, Narla AV, Pontrelli S et al. Stress-induced metabolic exchanges between complementary bacterial types underly a dynamic mechanism of inter-species stress resistance. Nat Commun. 2023;14:3165. 10.1038/s41467-023-38913-8. 37258505 PMC10232422

[bib11] Angeles-Martinez L, Hatzimanikatis V. Spatio-temporal modeling of the crowding conditions and metabolic variability in microbial communities. PLoS Comput Biol. 2021;17:e1009140. 10.1371/journal.pcbi.1009140. 34292935 PMC8297787

[bib12] Ascensao JA, Denk J, Lok K et al. Rediversification following ecotype isolation reveals hidden adaptive potential. Curr Biol. 2024;34:855–867 e6. 10.1016/j.cub.2024.01.029. 38325377 PMC10911448

[bib13] Attrill EL, Claydon R, Łapińska U et al. Individual bacteria in structured environments rely on phenotypic resistance to phage. PLOS Biol. 2021;19:e3001406. 10.1371/journal.pbio.3001406. 34637438 PMC8509860

[bib14] Averill C, Werbin ZR, Atherton KF et al. Soil microbiome predictability increases with spatial and taxonomic scale. Nat Ecol Evol. 2021;5:747–56. 10.1038/s41559-021-01445-9. 33888877

[bib15] Azimi S, Lewin GR, Whiteley M. The biogeography of infection revisited. Nat Rev Micro. 2022;20:579–92. 10.1038/s41579-022-00683-3. PMC9357866 35136217

[bib24] Baas Becking LGM Geobiologie of Inleiding Tot De Milieukunde. WP Van Stockum & Zoon, 1934.

[doi402_772_141526] Baas Becking LGM, Canfield DE Baas Becking's Geobiology. 2015. 10.1002/9781118295472

[bib16] Bach EM, Williams RJ, Hargreaves SK et al. Greatest soil microbial diversity found in micro-habitats. Soil Biol Biochem. 2018;118:217–26. 10.1016/j.soilbio.2017.12.018.

[bib17] Bahram M, Hildebrand F, Forslund SK et al. Structure and function of the global topsoil microbiome. Nature. 2018;560:233–237. 10.1038/s41586-018-0386-6. 30069051

[bib18] Baker JL, Mark Welch JL, Kauffman KM et al. The oral microbiome: diversity, biogeography and human health. Nat Rev Micro. 2024;22:89–104. 10.1038/s41579-023-00963-6. PMC11084736 37700024

[bib19] Bär J, Charlton SGV, Tarnutzer A et al. Single-cell approach dissecting agr quorum sensing dynamics in Staphylococcus aureus. bioRxiv. 2024:2024.02.27.582246. 10.1101/2024.02.27.582246 PMC13458059 42162019

[bib20] Basan M, Hui S, Okano H et al. Overflow metabolism in Escherichia coli results from efficient proteome allocation. Nature. 2015;528:99–104. 10.1038/nature15765. 26632588 PMC4843128

[bib21] Batsch M, Guex I, Todorov H et al. Fragmented micro-growth habitats present opportunities for alternative competitive outcomes. Nat Commun. 2024;15:7591. 10.1038/s41467-024-51944-z. 39217178 PMC11365936

[bib22] Bauer E, Zimmermann J, Baldini F et al. BacArena: individual-based metabolic modeling of heterogeneous microbes in complex communities. PLOS Comput Biol. 2017;13:e1005544. 10.1371/journal.pcbi.1005544. 28531184 PMC5460873

[bib23] Baym M, Lieberman TD, Kelsic ED et al. Spatiotemporal microbial evolution on antibiotic landscapes. Science. 2016;353:1147–51. 10.1126/science.aag0822. 27609891 PMC5534434

[bib25] Berngruber TW, Lion S, Gandon S. Spatial structure, transmission modes and the evolution of viral exploitation strategies. PLOS Pathog. 2015;11:e1004810. 10.1371/journal.ppat.1004810. 25898324 PMC4405370

[bib27] Bickel S, Or D. Aqueous habitats and carbon inputs shape the microscale geography and interaction ranges of soil bacteria. Commun Biol. 2023;6:1–10. 10.1038/s42003-023-04703-7. 36966207 PMC10039866

[bib26] Bickel S, Or D. Soil bacterial diversity mediated by microscale aqueous-phase processes across biomes. Nat Commun. 2020;11:116. 10.1038/s41467-019-13966-w. 31913270 PMC6949233

[bib28] Birzu G, Matin S, Hallatschek O et al. Genetic drift in range expansions is very sensitive to density dependence in dispersal and growth. Ecol Lett. 2019;22:1817–27. 10.1111/ele.13364. 31496047

[bib29] Blanchard AE, Lu T. Bacterial social interactions drive the emergence of differential spatial colony structures. BMC Syst Biol. 2015;9:59. 10.1186/s12918-015-0188-5. 26377684 PMC4573487

[bib30] Blasche S, Kim Y, Mars RAT et al. Metabolic cooperation and spatiotemporal niche partitioning in a kefir microbial community. Nat Microbiol. 2021;6:196–208. 10.1038/s41564-020-00816-5. 33398099 PMC7610452

[bib31] Blount ZD, Lenski RE, Losos JB. Contingency and determinism in evolution: replaying life’s tape. Science. 2018;362:eaam5979. 10.1126/science.aam5979. 30409860

[bib32] Boedicker JQ, Vincent ME, Ismagilov RF. Microfluidic confinement of single cells of bacteria in small volumes initiates high-density behavior of quorum sensing and growth and reveals its variability. Angew Chem Int Ed. 2009;48:5908–11. 10.1002/anie.200901550. PMC2748941 19565587

[bib33] Bond MC, Vidakovic L, Singh PK et al. Matrix-trapped viruses can prevent invasion of bacterial biofilms by colonizing cells. eLife. 2021;10:e65355. 10.7554/eLife.65355. 34240700 PMC8346279

[bib34] Booth SC, Meacock OJ, Foster KR. Cell motility empowers bacterial contact weapons. ISME J. 2024;18:wrae141. 10.1093/ismejo/wrae141. 39073907 PMC11482024

[bib35] Booth SC, Smith WPJ, Foster KR. The evolution of short- and long-range weapons for bacterial competition. Nat Ecol Evol. 2023;7:2080–91. 10.1038/s41559-023-02234-2. 38036633 PMC10697841

[bib36] Borenstein DB, Ringel P, Basler M et al. Established microbial colonies can survive type VI secretion assault. PLoS Comput Biol. 2015;11:e1004520. 10.1371/journal.pcbi.1004520. 26485125 PMC4619000

[bib37] Borer B, Ciccarese D, Johnson D et al. Spatial organization in microbial range expansion emerges from trophic dependencies and successful lineages. Commun Biol. 2020;3:1–10. 10.1038/s42003-020-01409-y. 33208809 PMC7674409

[bib38] Borin JM, Lee JJ, Lucia-Sanz A et al. Rapid bacteria-phage coevolution drives the emergence of multiscale networks. Science. 2023;382:674–8. 10.1126/science.adi5536. 37943920

[bib39] Bosshard L, Dupanloup I, Tenaillon O et al. Accumulation of deleterious mutations during bacterial range expansions. Genetics. 2017;207:669–84. 10.1534/genetics.117.300144. 28821588 PMC5629331

[bib40] Bottura B, Rooney LM, Hoskisson PA et al. Intra-colony channel morphology in Escherichia coli biofilms is governed by nutrient availability and substrate stiffness. Biofilm. 2022;4:100084. 10.1016/j.bioflm.2022.100084. 36254115 PMC9568850

[bib41] Bourceau P, Geier B, Suerdieck V et al. Visualization of metabolites and microbes at high spatial resolution using MALDI mass spectrometry imaging and in situ fluorescence labeling. Nat Protoc. 2023;18:3050–79. 10.1038/s41596-023-00864-1. 37674095

[bib42] Boza G, Barabás G, Scheuring I et al. Eco-evolutionary modelling of microbial syntrophy indicates the robustness of cross-feeding over cross-facilitation. Sci Rep. 2023;13:907. 10.1038/s41598-023-27421-w. 36650168 PMC9845244

[bib43] Bozdag GO, Zamani-Dahaj SA, Day TC et al. De novo evolution of macroscopic multicellularity. Nature. 2023;617:747–54. 10.1038/s41586-023-06052-1. 37165189 PMC10425966

[bib44] Bryant JA, Lamanna C, Morlon H et al. Microbes on mountainsides: contrasting elevational patterns of bacterial and plant diversity. Proc Natl Acad Sci USA. 2008;105:11505–11. 10.1073/pnas.0801920105. 18695215 PMC2556412

[bib45] Bull JJ, Christensen KA, Scott C et al. Phage-bacterial dynamics with spatial structure: self organization around Phage sinks can promote increased cell densities. Antibiotics. 2018;7:8. 10.3390/antibiotics7010008. 29382134 PMC5872119

[bib46] Cai YM. Non-surface attached bacterial aggregates: a ubiquitous third lifestyle. Front Microbiol. 2020;11:557035. 10.3389/fmicb.2020.557035. 33343514 PMC7746683

[bib47] Camenzind T, Mason-Jones K, Mansour I et al. Formation of necromass-derived soil organic carbon determined by microbial death pathways. Nat Geosci. 2023;16:115–22. 10.1038/s41561-022-01100-3.

[bib48] Candry P, Godfrey BJ, Winkler MK-H. Microbe-cellulose hydrogels as a model system for particulate carbon degradation in soil aggregates. ISME Commun. 2024;4:ycae068. 10.1093/ismeco/ycae068. 38800124 PMC11126157

[bib49] Cao Z, Zuo W, Wang L et al. Spatial profiling of microbial communities by sequential FISH with error-robust encoding. Nat Commun. 2023;14:1477. 10.1038/s41467-023-37188-3. 36932092 PMC10023729

[bib50] Capovilla G, Braakman R, Fournier GP et al. Chitin utilization by marine picocyanobacteria and the evolution of a planktonic lifestyle. Proc Natl Acad Sci U A. 2023;120:e2213271120. 10.1073/pnas.2213271120. PMC10194020 37159478

[bib51] Carson JK, Gonzalez-Quiñones V, Murphy DV et al. Low pore connectivity increases bacterial diversity in soil. Appl Environ Microb. 2010;76:3936–42. 10.1128/AEM.03085-09. PMC2893478 20418420

[bib52] Castledine M, Buckling A. Critically evaluating the relative importance of phage in shaping microbial community composition. Trends Microbiol. 2024;32:957–69. 10.1016/j.tim.2024.02.014. 38604881

[bib53] Chajwa R, Flaum E, Bidle KD et al. Hidden comet tails of marine snow impede ocean-based carbon sequestration. Science. 2024;386:eadl5767. 10.1126/science.adl5767. 39388567

[bib54] Chao L, Levin BR. Structured habitats and the evolution of anticompetitor toxins in bacteria. Proc Natl Acad Sci USA. 1981;78:6324–8. 10.1073/pnas.78.10.6324. 7031647 PMC349031

[bib55] Chen A, Liao S, Cheng M et al. Spatiotemporal transcriptomic atlas of mouse organogenesis using DNA nanoball-patterned arrays. Cell. 2022;185:1777–92.e21. 10.1016/j.cell.2022.04.003. 35512705

[bib56] Chesneau G, Herpell J, Garrido-Oter R et al. From synthetic communities to synthetic ecosystems: exploring causalities in plant–microbe–environment interactions. New Phytol. 2025;245:496–502. 10.1111/nph.20250. 39501565

[bib57] Chesson P. Scale transition theory: its aims, motivations and predictions. Ecol Complex. 2012;10:52–68. 10.1016/j.ecocom.2011.11.002.

[bib58] Choudhary D, Lagage V, Foster KR et al. Phenotypic heterogeneity in the bacterial oxidative stress response is driven by cell-cell interactions. Cell Rep. 2023;42:112168. 10.1016/j.celrep.2023.112168. 36848288 PMC10935545

[bib59] Chuang JS, Rivoire O, Leibler S. Simpson’s paradox in a synthetic microbial system. Science. 2009;323:272–5. 10.1126/science.1166739. 19131632

[bib60] Claesen J, Spagnolo JB, Ramos SF et al. A cutibacterium acnes antibiotic modulates human skin microbiota composition in hair follicles. Sci Transl Med. 2020;12:eaay5445. 10.1126/scitranslmed.aay5445. 33208503 PMC8478231

[bib61] Cole JA, Kohler L, Hedhli J et al. Spatially-resolved metabolic cooperativity within dense bacterial colonies. BMC Syst Biol. 2015;9:15. 10.1186/s12918-015-0155-1. 25890263 PMC4376365

[bib62] Colizzi ES, Hogeweg P. Evolution of functional diversification within quasispecies. Genome Biol Evol. 2014;6:1990–2007. 10.1093/gbe/evu150. 25056399 PMC4159002

[bib63] Colizzi ES, Hogeweg P. High cost enhances cooperation through the interplay between evolution and self-organisation. Bmc Evol Biol. 2016a;16:31. 10.1186/s12862-016-0600-9. 26832152 PMC4736645

[bib64] Colizzi ES, Hogeweg P. Parasites sustain and enhance RNA-like replicators through spatial self-organisation. PLoS Comput Biol. 2016b;12:e1004902. 10.1371/journal.pcbi.1004902. 27120344 PMC4847872

[bib65] Colizzi ES, van Dijk B, Merks RMH et al. Evolution of genome fragility enables microbial division of labor. Mol Syst Biol. 2023;19:e11353. 10.15252/msb.202211353. 36727665 PMC9996244

[bib66] Connell JL, Kim J, Shear JB et al. Real-time monitoring of quorum sensing in 3D-printed bacterial aggregates using scanning electrochemical microscopy. Proc Natl Acad Sci USA. 2014;111:18255–60. 10.1073/pnas.1421211111. 25489085 PMC4280622

[bib67] Connell JL, Ritschdorff ET, Whiteley M et al. 3D printing of microscopic bacterial communities. Proc Natl Acad Sci USA. 2013;110:18380–5. 10.1073/pnas.1309729110. 24101503 PMC3832025

[bib68] Conwill A, Kuan AC, Damerla R et al. Anatomy promotes neutral coexistence of strains in the human skin microbiome. Cell Host Microbe. 2022;30:171–182.e7. 10.1016/j.chom.2021.12.007. 34995483 PMC8831475

[bib69] Cornforth DM, Foster KR. Antibiotics and the art of bacterial war. Proc Natl Acad Sci USA. 2015;112:10827–8. 10.1073/pnas.1513608112. 26305963 PMC4568221

[bib70] Cremer J, Honda T, Tang Y et al. Chemotaxis as a navigation strategy to boost range expansion. Nature. 2019;575:658–63. 10.1038/s41586-019-1733-y. 31695195 PMC6883170

[bib71] Czárán T, Hoekstra RF. A spatial model of the evolution of quorum sensing regulating bacteriocin production. Behav Ecol. 2007;18:866–73. 10.1093/beheco/arm061.

[bib72] Czárán T, Hoekstra RF. Microbial communication, cooperation and cheating: quorum sensing drives the evolution of cooperation in bacteria. PLoS One. 2009;4:e6655. 10.1371/journal.pone.0006655. 19684853 PMC2722019

[bib73] Czárán TL, Hoekstra RF, Pagie L. Chemical warfare between microbes promotes biodiversity. Proc Natl Acad Sci USA. 2002;99:786–90. 10.1073/pnas.012399899. 11792831 PMC117383

[bib100] D’Souza G, Kost C. Experimental evolution of metabolic dependency in bacteria. PLoS Genet. 2016;12:e1006364. 27814362 10.1371/journal.pgen.1006364 PMC5096674

[bib101] D’Souza G, Schwartzman J, Keegstra J et al. Interspecies interactions determine growth dynamics of biopolymer-degrading populations in microbial communities. Proc Natl Acad Sci U A. 2023;120:e2305198120. 10.1073/pnas.2305198120 PMC10622921 37878716

[bib74] Dal Bello M, Lee H, Goyal A et al. Resource–diversity relationships in bacterial communities reflect the network structure of microbial metabolism. Nat Ecol Evol. 2021;5:1424–34. 10.1038/s41559-021-01535-8. 34413507

[bib75] Dal Co A, Ackermann M, van Vliet S. Metabolic activity affects the response of single cells to a nutrient switch in structured populations. J R Soc Interface. 2019a;16:20190182. 10.1098/rsif.2019.0182. 31288652 PMC6685030

[bib76] Dal Co A, Ackermann M, van Vliet S. Spatial self-organization of metabolism in microbial systems: a matter of enzymes and chemicals. Cell Syst. 2023;14:98–108. 10.1016/j.cels.2022.12.009. 36796335

[bib77] Dal Co A, van Vliet S, Ackermann M. Emergent microscale gradients give rise to metabolic cross-feeding and antibiotic tolerance in clonal bacterial populations. Philos Trans R Soc B Biol Sci. 2019b;374:20190080. 10.1098/rstb.2019.0080. PMC6792440 31587651

[bib78] Dal Co A, van Vliet S, Kiviet DJ et al. Short-range interactions govern the dynamics and functions of microbial communities. Nat Ecol Evol. 2020;4:366–75. 10.1038/s41559-019-1080-2. 32042125

[bib79] Dar D, Dar N, Cai L et al. Spatial transcriptomics of planktonic and sessile bacterial populations at single-cell resolution. Science. 2021;373:eabi4882. 10.1126/science.abi4882. 34385369 PMC8454218

[bib80] Darch SE, Simoska O, Fitzpatrick M et al. Spatial determinants of quorum signaling in a Pseudomonas aeruginosa infection model. Proc Natl Acad Sci USA. 2018;115:4779–84. 10.1073/pnas.1719317115. 29666244 PMC5939081

[bib81] Darwin C. The Formation of Vegetable Mould, through the Action of Worms, with Observations on Their Habits. England: J. Murray, 1892.

[bib82] Das T, P. Krom B, Mei HCvd et al. DNA-mediated bacterial aggregation is dictated by acid–base interactions. Soft Matter. 2011;7:2927–35. 10.1039/c0sm01142h.

[bib83] Datta MS, Sliwerska E, Gore J et al. Microbial interactions lead to rapid micro-scale successions on model marine particles. Nat Commun. 2016;7:11965. 10.1038/ncomms11965. 27311813 PMC4915023

[bib90] De Wit R, Bouvier T.‘Everything is everywhere, but, the environment selects’; what did Baas Becking and Beijerinck really say?. Environ Microbiol. 2006;8:755–8. 10.1111/j.1462-2920.2006.01017.x. 16584487

[bib84] Debray R, Herbert RA, Jaffe AL et al. Priority effects in microbiome assembly. Nat Rev Micro. 2022;20:109–21. 10.1038/s41579-021-00604-w. 34453137

[bib85] Delarue M, Hartung J, Schreck C et al. Self-driven jamming in growing microbial populations. Nat Phys. 2016;12:762–6. 10.1038/nphys3741. 27642362 PMC5022770

[bib86] Delgado-Baquerizo M, Oliverio AM, Brewer TE et al. A global atlas of the dominant bacteria found in soil. Science. 2018;359:320–325. 10.1126/science.aap9516. 29348236

[bib87] Delmont TO, Kiefl E, Kilinc O et al. Single-amino acid variants reveal evolutionary processes that shape the biogeography of a global SAR11 subclade. Rainey PB, Weigel D, Rainey PB et al. (eds.). eLife. 2019;8:e46497. 10.7554/eLife.46497. 31478833 PMC6721796

[bib88] DeLong EF, Preston CM, Mincer T et al. Community genomics among stratified microbial assemblages in the ocean’s interior. Science. 2006;311:496–503. 10.1126/science.1120250. 16439655

[bib89] Denk-Lobnig MK, Wood KB. Spatial population dynamics of bacterial colonies with social antibiotic resistance. Proc Natl Acad Sci USA. 2025;122:e2417065122. 10.1073/pnas.2417065122. 39937854 PMC11848446

[bib91] Díaz-Pascual F, Lempp M, Nosho K et al. Spatial alanine metabolism determines local growth dynamics of Escherichia coli colonies. Xavier KB, Storz G, Xavier KB (eds.). eLife. 2021;10:e70794. 10.7554/eLife.70794. 34751128 PMC8579308

[bib92] Dick GJ. The microbiomes of deep-sea hydrothermal vents: distributed globally, shaped locally. Nat Rev Micro. 2019;17:271–83. 10.1038/s41579-019-0160-2. 30867583

[bib93] Diener C, Gibbons SM, Resendis-Antonio O. MICOM: metagenome-scale modeling to infer metabolic interactions in the gut microbiota. Msystems. 2020;5:e00606–19. 10.1128/msystems.00606-19. 31964767 PMC6977071

[bib94] Dockery J, Klapper I. Finger formation in biofilm layers. SIAM J Appl Math. 2001;62:853–69.

[bib95] Doekes HM, Boer RJd, Hermsen R. Toxin production spontaneously becomes regulated by local cell density in evolving bacterial populations. PLOS Comput Biol. 2019;15:e1007333. 10.1371/journal.pcbi.1007333. 31469819 PMC6742444

[bib96] Doekes HM, Hermsen R. Multiscale selection in spatially structured populations. Proc. R. Soc. B. Biol. Sci. 2024;291:20232559. 10.1098/rspb.2023.2559. PMC11285734 38808450

[bib97] Doekes HM, Mulder GA, Hermsen R. Repeated outbreaks drive the evolution of bacteriophage communication. Díaz-Muñoz SL, Walczak AM, Díaz-Muñoz SL (eds.). eLife. 2021;10:e58410. 10.7554/eLife.58410. 33459590 PMC7935489

[bib98] Dorken G, Ferguson GP, French CE et al. Aggregation by depletion attraction in cultures of bacteria producing exopolysaccharide. J R Soc Interface. 2012;9:3490–502. 10.1098/rsif.2012.0498. 22896568 PMC3481587

[bib99] Drescher K, Dunkel J, Nadell CD et al. Architectural transitions in vibrio cholerae biofilms at single-cell resolution. Proc Natl Acad Sci USA. 2016;113:E2066–72. 10.1073/pnas.1601702113. 26933214 PMC4833255

[bib102] Dukovski I, Bajic D, Chacón JM et al. A metabolic modeling platform for the computation of microbial ecosystems in time and space (COMETS). Nat Protoc. 2021;16:5030–5082. 10.1038/s41596-021-00593-3. 34635859 PMC10824140

[bib103] Dukovski I, Golden L, Zhang J et al. Biophysical metabolic modeling of complex bacterial colony morphology. Cell Syst. 2025;16:101352. 10.1016/j.cels.2025.101352. 40782802 PMC12393670

[bib104] Ebrahimi A, Goyal A, Cordero OX. Particle foraging strategies promote microbial diversity in marine environments. eLife. 2022;11:e73948. 10.7554/eLife.73948. 35289269 PMC8956285

[bib105] Ebrahimi A, Schwartzman J, Cordero OX. Cooperation and spatial self-organization determine rate and efficiency of particulate organic matter degradation in marine bacteria. Proc Natl Acad Sci USA. 2019a;116:23309–16. 10.1073/pnas.1908512116. 31666322 PMC6859336

[bib106] Ebrahimi A, Schwartzman J, Cordero OX. Multicellular behaviour enables cooperation in microbial cell aggregates. Philos Trans R Soc B-Biol Sci. 2019b;374:20190077. 10.1098/rstb.2019.0077. PMC6792450 31587643

[bib397_732_135426] Edmonds CA, Lillie AS and Cavalli-Sforza LL. Mutations arising in the wave front of an expanding population. Proc Natl Acad Sci U S A. 2004;101:975–9. 14732681 10.1073/pnas.0308064100 PMC327127

[bib107] Eigen M, McCaskill J, Schuster P. Molecular quasi-species. J Phys Chem. 1988;92:6881–91. 10.1021/j100335a010.

[bib108] Eigentler L, Kalamara M, Ball G et al. Founder cell configuration drives competitive outcome within colony biofilms. ISME J. 2022;16:1512–22. 10.1038/s41396-022-01198-8. 35121821 PMC9122948

[bib109] Enke TN, Datta MS, Schwartzman J et al. Modular assembly of polysaccharide-degrading marine microbial communities. Curr Biol. 2019;29:1528–1535. 10.1016/j.cub.2019.03.047. 31031118

[bib110] Enke TN, Leventhal GE, Metzger M et al. Microscale ecology regulates particulate organic matter turnover in model marine microbial communities. Nat Commun. 2018;9:2743. 10.1038/s41467-018-05159-8. 30013041 PMC6048024

[bib111] Erez Z, Steinberger-Levy I, Shamir M et al. Communication between viruses guides lysis–lysogeny decisions. Nature. 2017;541:488–93. 10.1038/nature21049. 28099413 PMC5378303

[bib112] Eriksen RS, Mitarai N, Sneppen K. Sustainability of spatially distributed bacteria-phage systems. Sci Rep. 2020;10:3154. 10.1038/s41598-020-59635-7. 32081858 PMC7035299

[bib113] Estrela S, Vila JCC, Lu N et al. Functional attractors in microbial community assembly. Cell Syst. 2022;13:29–42.e7. 10.1016/j.cels.2021.09.011. 34653368 PMC8800145

[bib114] Evans CR, Smiley MK, Asahara Thio S et al. Spatial heterogeneity in biofilm metabolism elicited by local control of phenazine methylation. Proc Natl Acad Sci U A. 2023;120:e2313208120. 10.1073/pnas.2313208120. PMC10614215 37847735

[bib115] Excoffier L, Ray N. Surfing during population expansions promotes genetic revolutions and structuration. Trends Ecol Evol. 2008;23:347–51. 10.1016/j.tree.2008.04.004. 18502536

[bib116] Fahrig L. Ecological responses to habitat fragmentation per Se. Annu Rev Ecol Evol Syst. 2017;48:1–23. 10.1146/annurev-ecolsys-110316-022612.

[bib117] Falkowski PG, Fenchel T, Delong EF. The microbial engines that drive earth’s biogeochemical cycles. Science. 2008;320:1034–9. 10.1126/science.1153213. 18497287

[bib118] Falony G, Joossens M, Vieira-Silva S et al. Population-level analysis of gut microbiome variation. Science. 2016;352:560–4. 10.1126/science.aad3503. 27126039

[bib119] Fierer N, Jackson RB. The diversity and biogeography of soil bacterial communities. Proc Natl Acad Sci USA. 2006;103:626–31. 10.1073/pnas.0507535103. 16407148 PMC1334650

[bib120] Flemming HC, Wuertz S. Bacteria and archaea on Earth and their abundance in biofilms. Nat Rev Micro. 2019;17:247–60. 10.1038/s41579-019-0158-9. 30760902

[bib121] Flynn KJ, Mitra A, Wilson WH et al.‘Boom-and-busted’ dynamics of phytoplankton–virus interactions explain the paradox of the plankton. New Phytol. 2022;234:990–1002. 10.1111/nph.18042. 35179778 PMC9313554

[bib122] Foster KR, Bell T. Competition, not cooperation, dominates interactions among culturable microbial species. Curr Biol. 2012;22:1845–50. 10.1016/j.cub.2012.08.005. 22959348

[bib124] France MT, Forney LJ. The relationship between spatial structure and the maintenance of diversity in microbial populations. Am Nat. 2019;193:503–13. 10.1086/701799. 30912968

[bib125] Frey E. Evolutionary game theory: theoretical concepts and applications to microbial communities. Phys Stat Mech Its Appl. 2010;389:4265–98. 10.1016/j.physa.2010.02.047.

[bib126] Friedman J, Higgins LM, Gore J. Community structure follows simple assembly rules in microbial microcosms. Nat Ecol Evol. 2017;1:1–7. 10.1038/s41559-017-0109. 28812687

[bib127] Frost I, Smith WPJ, Mitri S et al. Cooperation, competition and antibiotic resistance in bacterial colonies. ISME J. 2018;12:1582–93. 10.1038/s41396-018-0090-4. 29563570 PMC5955900

[bib128] Fuhrman JA, Steele JA, Hewson I et al. A latitudinal diversity gradient in planktonic marine bacteria. Proc Natl Acad Sci USA. 2008;105:7774–8. 10.1073/pnas.0803070105. 18509059 PMC2409396

[bib129] Fullmer MS, van Dijk B, Takeuchi N. Interaction range of common goods shapes Black Queen dynamics beyond the cheater-cooperator narrative. 2024:2024.07.16.603646. bioRxiv. 10.1098/rspb.2026.0911 42379595

[bib130] Fusco D, Gralka M, Kayser J et al. Excess of mutational jackpot events in expanding populations revealed by spatial Luria–Delbrück experiments. Nat Commun. 2016;7:12760. 10.1038/ncomms12760. 27694797 PMC5059437

[bib131] Garcia-Garcera M, Rocha EPC. Community diversity and habitat structure shape the repertoire of extracellular proteins in bacteria. Nat Commun. 2020;11:758. 10.1038/s41467-020-14572-x. 32029728 PMC7005277

[bib132] Garza DR, Dutilh BE. From cultured to uncultured genome sequences: metagenomics and modeling microbial ecosystems. Cell Mol Life Sci. 2015;72:4287–308. 10.1007/s00018-015-2004-1. 26254872 PMC4611022

[bib133] Garza DR, von Meijenfeldt FAB, van Dijk B et al. Nutrition or nature: using elementary flux modes to disentangle the complex forces shaping prokaryote pan-genomes. BMC Ecol Evol. 2022;22:101. 10.1186/s12862-022-02052-3. 35974327 PMC9382767

[bib134] Geier B, Sogin EM, Michellod D et al. Spatial metabolomics of in situ host–microbe interactions at the micrometre scale. Nat Microbiol. 2020;5:498–510. 10.1038/s41564-019-0664-6. 32015496

[bib135] Giovannoni SJ, Thrash JC, Temperton B. Implications of streamlining theory for microbial ecology. ISME J. 2014;8:1553–65. 10.1038/ismej.2014.60. 24739623 PMC4817614

[bib136] Giri S, Yousif G, Shitut S et al. Prevalent emergence of reciprocity among cross-feeding bacteria. ISME Commun. 2022;2:71. 10.1038/s43705-022-00155-y. 37938764 PMC9723789

[bib137] Gluck-Thaler E, Ralston T, Konkel Z et al. Giant starship elements mobilize accessory genes in fungal genomes. Mol Biol Evol. 2022;39:msac109. 10.1093/molbev/msac109. 35588244 PMC9156397

[bib138] Goldford JE, Lu N, Bajić D et al. Emergent simplicity in microbial community assembly. Science. 2018;361:469–74. 10.1126/science.aat1168. 30072533 PMC6405290

[bib139] Goldschmidt F, Caduff L, Johnson DR. Causes and consequences of pattern diversification in a spatially self-organizing microbial community. ISME J. 2021;15:2415–26. 10.1038/s41396-021-00942-w. 33664433 PMC8319339

[bib141] Goldschmidt F, Regoes RR, Johnson DR. Metabolite toxicity slows local diversity loss during expansion of a microbial cross-feeding community. ISME J. 2018;12:136–44. 10.1038/ismej.2017.147. 28914879 PMC5739007

[bib140] Goldschmidt F, Regoes RR, Johnson DR. Successive range expansion promotes diversity and accelerates evolution in spatially structured microbial populations. ISME J. 2017;11:2112–23. 10.1038/ismej.2017.76. 28534878 PMC5563963

[bib142] Gómez-Consarnau L, Raven JA, Levine NM et al. Microbial rhodopsins are major contributors to the solar energy captured in the sea. Sci Adv. 2019;5:eaaw8855. 10.1126/sciadv.aaw8855. 31457093 PMC6685716

[bib143] Gorter FA, Manhart M, Ackermann M. Understanding the evolution of interspecies interactions in microbial communities. Philos Trans R Soc B Biol Sci. 2020;375:20190256. 10.1098/rstb.2019.0256. PMC7133538 32200743

[bib144] Gralka M, Hallatschek O. Environmental heterogeneity can tip the population genetics of range expansions. eLife. 2019;8:e44359. 10.7554/eLife.44359. 30977724 PMC6513619

[bib145] Gralka M, Pollak S, Cordero OX. Genome content predicts the carbon catabolic preferences of heterotrophic bacteria. Nat Microbiol. 2023;8:1799–808. 10.1038/s41564-023-01458-z. 37653010

[bib146] Gralka M, Stiewe F, Farrell F et al. Allele surfing promotes microbial adaptation from standing variation. Ecol Lett. 2016;19:889–98. 10.1111/ele.12625. 27307400 PMC4942372

[bib147] Gralka M, Szabo R, Stocker R et al. Trophic interactions and the drivers of microbial community assembly. Curr Biol. 2020;30:R1176–88. 10.1016/j.cub.2020.08.007. 33022263

[bib148] Grimm V, Revilla E, Berger U et al. Pattern-oriented modeling of agent-based Complex systems: lessons from ecology. Science. 2005;310:987–91. 10.1126/science.1116681. 16284171

[bib149] Gude S, Pinçe E, Taute KM et al. Bacterial coexistence driven by motility and spatial competition. Nature. 2020;578:588–92. 10.1038/s41586-020-2033-2. 32076271

[bib150] Guessous G, Patsalo V, Balakrishnan R et al. Inherited chitinases enable sustained growth and rapid dispersal of bacteria from chitin particles. Nat Microbiol. 2023;8:1695–705. 10.1038/s41564-023-01444-5. 37580592 PMC13050353

[bib151] Hallatschek O, Nelson DR. Life at the front of an expanding population. Evolution. 2010;64:193–206. 10.1111/j.1558-5646.2009.00809.x. 19682067

[bib152] Hallatschek O. Selection-like biases emerge in population models with recurrent jackpot events. Genetics. 2018;210:1053–73. 10.1534/genetics.118.301516. 30171032 PMC6218241

[bib153] Hamidjaja R, Capoulade J, Catón L et al. The cell organization underlying structural colour is involved in Flavobacterium IR1 predation. ISME J. 2020;14:2890–900. 10.1038/s41396-020-00760-6. 32873891 PMC7784876

[bib154] Handel A, Rozen DE. The impact of population size on the evolution of asexual microbes on smooth versus rugged fitness landscapes. BMC Evol Biol. 2009;9:236. 10.1186/1471-2148-9-236. 19765292 PMC2753573

[bib155] Hartmann M, Six J. Soil structure and microbiome functions in agroecosystems. Nat Rev Earth Environ. 2022;4:4–18. 10.1038/s43017-022-00366-w.

[bib156] Hartmann R, Jeckel H, Jelli E et al. Quantitative image analysis of microbial communities with BiofilmQ. Nat Microbiol. 2021;6:151–6. 10.1038/s41564-020-00817-4. 33398098 PMC7840502

[bib157] Hartmann R, Singh PK, Pearce P et al. Emergence of three-dimensional order and structure in growing biofilms. Nat Phys. 2019;15:251–6. 10.1038/s41567-018-0356-9. 31156716 PMC6544526

[bib158] Hehemann J-H, Arevalo P, Datta MS et al. Adaptive radiation by waves of gene transfer leads to fine-scale resource partitioning in marine microbes. Nat Commun. 2016;7:12860. 10.1038/ncomms12860. 27653556 PMC5036157

[bib159] Henderson A, Del Panta A, Schubert OT et al. Disentangling the feedback loops driving spatial patterning in microbial communities. npj Biofilms Microbiomes. 2025;11:1–14. 10.1038/s41522-025-00666-1. 39979272 PMC11842706

[bib160] Hermsen R. Emergent multilevel selection in a simple spatial model of the evolution of altruism. PLOS Comput Biol. 2022;18:e1010612. 10.1371/journal.pcbi.1010612. 36282807 PMC9595567

[bib161] Hesse E, O’Brien S. Ecological dependencies and the illusion of cooperation in microbial communities. Microbiol Read. 2024;170:001442. 10.1099/mic.0.001442 PMC10924460 38385784

[bib162] Hogeweg P. Cellular automata as a paradigm for ecological modeling. Appl Math Comput. 1988;27:81–100.

[bib163] Hogeweg P. From population dynamics to ecoinformatics: ecosystems as multilevel information processing systems. Ecol Inform. 2007;2:103–11. 10.1016/j.ecoinf.2007.01.002.

[bib164] Hormoz S, Desprat N, Shraiman BI. Inferring epigenetic dynamics from kin correlations. Proc Natl Acad Sci USA. 2015;112:E2281–9. 10.1073/pnas.1504407112. 25902540 PMC4426465

[bib165] Hsu RH, Clark RL, Tan JW et al. Microbial interaction network inference in microfluidic droplets. Cell Syst. 2019;9:229–242.e4. 10.1016/j.cels.2019.06.008. 31494089 PMC6763379

[bib166] Huelsmann M, Schubert OT, Ackermann M. A framework for understanding collective microbiome metabolism. Nat Microbiol. 2024;9:3097–109. 10.1038/s41564-024-01850-3. 39604625

[bib167] Hui S, Silverman JM, Chen SS et al. Quantitative proteomic analysis reveals a simple strategy of global resource allocation in bacteria. Mol Syst Biol. 2015;11:784. 10.15252/msb.20145697. 25678603 PMC4358657

[bib168] Huisman JS, Bernhard A, Igler C. Should I stay or should I go: transmission trade-offs in phages and plasmids. Trends Microbiol. 2025;33:484–95. 10.1016/j.tim.2025.01.007. 39979200

[bib169] Huttenhower C, Gevers D, Knight R et al. Structure, function and diversity of the healthy human microbiome. Nature. 2012;486:207–14. 22699609 10.1038/nature11234 PMC3564958

[bib170] Jeckel H, Nosho K, Neuhaus K et al. Simultaneous spatiotemporal transcriptomics and microscopy of Bacillus subtilis swarm development reveal cooperation across generations. Nat Microbiol. 2023;8:2378–91. 10.1038/s41564-023-01518-4. 37973866 PMC10686836

[bib171] Jensen MA, Faruque SM, Mekalanos JJ et al. Modeling the role of bacteriophage in the control of cholera outbreaks. Proc Natl Acad Sci USA. 2006;103:4652–7. 10.1073/pnas.0600166103. 16537404 PMC1450226

[bib172] Jin X, Yu FB, Yan J et al. Culturing of a complex gut microbial community in mucin-hydrogel carriers reveals strain- and gene-associated spatial organization. Nat Commun. 2023;14:3510. 10.1038/s41467-023-39121-0. 37316519 PMC10267222

[bib173] Jo J, Price-Whelan A, Dietrich LEP. Gradients and consequences of heterogeneity in biofilms. Nat Rev Micro. 2022;20:593–607. 10.1038/s41579-022-00692-2. PMC9590228 35149841

[bib174] Johansen VE, Catón L, Hamidjaja R et al. Genetic manipulation of structural color in bacterial colonies. Proc Natl Acad Sci USA. 2018;115:2652–7. 10.1073/pnas.1716214115. 29472451 PMC5856530

[bib175] Jonge PAd, Nobrega FL, Brouns SJJ et al. Molecular and evolutionary determinants of bacteriophage host range. Trends Microbiol. 2019;27:51–63. 10.1016/j.tim.2018.08.006. 30181062

[bib176] Julou T, Mora T, Guillon L et al. Cell–cell contacts confine public goods diffusion inside Pseudomonas aeruginosa clonal microcolonies. Proc Natl Acad Sci USA. 2013;110:12577–82. 10.1073/pnas.1301428110. 23858453 PMC3732961

[bib177] Kearns DB, Losick R. Swarming motility in undomesticated Bacillus subtilis. Mol Microbiol. 2003;49:581–90. 10.1046/j.1365-2958.2003.03584.x. 12864845

[bib178] Kearns DB. A field guide to bacterial swarming motility. Nat Rev Micro. 2010;8:634–44. 10.1038/nrmicro2405. PMC3135019 20694026

[bib179] Keegstra JM, Carrara F, Stocker R. The ecological roles of bacterial chemotaxis. Nat Rev Micro. 2022;20:491–504. 10.1038/s41579-022-00709-w. 35292761

[bib180] Keller L, Surette MG. Communication in bacteria: an ecological and evolutionary perspective. Nat Rev Micro. 2006;4:249–58. 10.1038/nrmicro1383. 16501584

[bib181] Kerr B, Riley MA, Feldman MW et al. Local dispersal promotes biodiversity in a real-life game of rock–paper–scissors. Nature. 2002;418:171–4. 10.1038/nature00823. 12110887

[bib182] Keymer JE, Galajda P, Muldoon C et al. Bacterial metapopulations in nanofabricated landscapes. Proc Natl Acad Sci USA. 2006;103:17290–5. 10.1073/pnas.0607971103. 17090676 PMC1635019

[bib183] Kirkup BC, Riley MA. Antibiotic-mediated antagonism leads to a bacterial game of rock–paper–scissors in vivo. Nature. 2004;428:412–4. 10.1038/nature02429. 15042087

[bib399_852_133526] Klopfstein S, Currat M and Excoffier L. The fate of mutations surfing on the wave of a range expansion. Mol Biol Evol. 2006;23:482–90. 16280540 10.1093/molbev/msj057

[bib184] Knowles B, Silveira CB, Bailey BA et al. Lytic to temperate switching of viral communities. Nature. 2016;531:466–70. 10.1038/nature17193. 26982729

[bib185] Kost C, Patil KR, Friedman J et al. Metabolic exchanges are ubiquitous in natural microbial communities. Nat Microbiol. 2023;8:2244–52. 10.1038/s41564-023-01511-x. 37996708

[bib186] Kotil SE, Vetsigian K. Emergence of evolutionarily stable communities through eco-evolutionary tunnelling. Nat Ecol Evol. 2018;2:1644–53. 10.1038/s41559-018-0655-7. 30242295

[bib187] Kragh KN, Hutchison JB, Melaugh G et al. Role of multicellular aggregates in biofilm formation. mBio. 2016;7.e00237. 10.1128/mbio.00237-16. 27006463 PMC4807362

[bib188] Kraigher B, Butolen M, Stefanic P et al. Kin discrimination drives territorial exclusion during swarming and restrains exploitation of surfactin. ISME J. 2022;16:833–41. 10.1038/s41396-021-01124-4. 34650232 PMC8857193

[bib189] Kreft M, Lukšič M, Zorec TM et al. Diffusion of d-glucose measured in the cytosol of a single astrocyte. Cell Mol Life Sci CMLS. 2012;70:1483–92. 10.1007/s00018-012-1219-7. 23224430 PMC11113596

[doi401_746_143426] Krishna Kumar R, Foster KR. 3D printing of microbial communities: A new platform for understanding and engineering microbiomes. Microbial Biotechnology. 2022;16:489–493. 10.1111/1751-7915.14168 36511313 PMC9948180

[doi400_782_142926] Krishna Kumar R, Meiller-Legrand TA., Alcinesio A et al. Droplet printing reveals the importance of micron-scale structure for bacterial ecology. Nature Communications. 2021;12. 10.1038/s41467-021-20996-w PMC7870943 33558498

[bib191] Kylafis G, Loreau M. Niche construction in the light of niche theory. Ecol Lett. 2011;14:82–90. 10.1111/j.1461-0248.2010.01551.x. 21073644

[bib192] Ladau J, Eloe-Fadrosh EA. Spatial, temporal, and phylogenetic scales of microbial ecology. Trends Microbiol. 2019;27:662–9. 10.1016/j.tim.2019.03.003. 31000488

[bib193] Laland K, Matthews B, Feldman MW. An introduction to niche construction theory. Evol Ecol. 2016;30:191–202. 10.1007/s10682-016-9821-z. 27429507 PMC4922671

[bib194] Langille M, San Roman M, Wagner A. An enormous potential for niche construction through bacterial cross-feeding in a homogeneous environment. PLOS Comput Biol. 2018;14:e1006340. 30040834 10.1371/journal.pcbi.1006340 PMC6080805

[bib195] Lardon LA, Merkey BV, Martins S et al. iDynoMiCS: next-generation individual-based modelling of biofilms. Environ Microbiol. 2011;13:2416–34. 10.1111/j.1462-2920.2011.02414.x. 21410622

[bib196] Laventie B-J, Sangermani M, Estermann F et al. A surface-induced asymmetric program promotes tissue colonization by Pseudomonas aeruginosa. Cell Host Microbe. 2019;25:140–52.e6. 10.1016/j.chom.2018.11.008. 30581112

[bib197] Lee J-Y, Sadler NC, Egbert RG et al. Deep learning predicts microbial interactions from self-organized spatiotemporal patterns. Comput Struct Biotechnol J. 2020;18:1259–69. 10.1016/j.csbj.2020.05.023. 32612750 PMC7298420

[bib198] Lega J, Passot T. Hydrodynamics of bacterial colonies: a model. Phys Rev E. 2003;67:031906. 10.1103/PhysRevE.67.031906. 12689100

[bib199] Lehmann J, Hansel CM, Kaiser C et al. Persistence of soil organic carbon caused by functional complexity. Nat Geosci. 2020;13:529–34. 10.1038/s41561-020-0612-3.

[bib200] Leibold MA, Holyoak M, Mouquet N et al. The metacommunity concept: a framework for multi-scale community ecology. Ecol Lett. 2004;7:601–13. 10.1111/j.1461-0248.2004.00608.x.

[bib201] Levins R, Lewontin R. The Dialectical Biologist. Unites States: Harvard University Press, 1985.

[bib202] Li B, Taniguchi D, Gedara JP et al. NUFEB: a massively parallel simulator for individual-based modelling of microbial communities. PLOS Comput Biol. 2019;15:e1007125. 10.1371/journal.pcbi.1007125. 31830032 PMC6932830

[bib203] Li C, Hurley A, Hu W et al. Social motility of biofilm-like microcolonies in a gliding bacterium. Nat Commun. 2021;12:5700. 10.1038/s41467-021-25408-7. 34588437 PMC8481357

[bib204] Li Z, Kravchenko AN, Cupples A et al. Composition and metabolism of microbial communities in soil pores. Nat Commun. 2024;15:3578. 10.1038/s41467-024-47755-x. 38678028 PMC11055953

[bib205] Lieberman E, Hauert C, Nowak MA. Evolutionary dynamics on graphs. Nature. 2005;433:312–6. 10.1038/nature03204. 15662424

[bib206] Limoli DH, Warren EA, Yarrington KD et al. Interspecies interactions induce exploratory motility in Pseudomonas aeruginosa. Newman DK, Storz G, Wolfgang M (eds.). eLife. 2019;8:e47365. 10.7554/eLife.47365. 31713513 PMC6910820

[bib207] Liu W, Tokuyasu TA, Fu X et al. The spatial organization of microbial communities during range expansion. Curr Opin Microbiol. 2021;63:109–16. 10.1016/j.mib.2021.07.005. 34329942

[bib208] Lloyd DP, Allen RJ. Competition for space during bacterial colonization of a surface. J R Soc Interface. 2015;12:20150608. 10.1098/rsif.2015.0608. 26333814 PMC4614474

[bib209] Lobanov A, Dyckman S, Kurkjian H et al. Spatial structure favors microbial coexistence except when slower mediator diffusion weakens interactions. eLife. 2023;12:e82504. 10.7554/eLife.82504. 37350317 PMC10348751

[bib210] Lopes W, Amor DR, Gore J. Cooperative growth in microbial communities is a driver of multistability. Nat Commun. 2024;15:4709. 10.1038/s41467-024-48521-9. 38830891 PMC11148146

[bib211] Lopez JL, Fourie A, Poppeliers SWM et al. Growth rate is a dominant factor predicting the rhizosphere effect. ISME J. 2023;17:1396–405. 10.1038/s41396-023-01453-6. 37322285 PMC10432406

[bib212] López-Pagán N, Rufián JS, Luneau J et al. Pseudomonas syringae subpopulations cooperate by coordinating flagellar and type III secretion spatiotemporal dynamics to facilitate plant infection. Nat Microbiol. 2025;10:958–72. 40175722 10.1038/s41564-025-01966-0 PMC11964935

[bib213] Lotstedt B, Strazar M, Xavier R et al. Spatial host-microbiome sequencing reveals niches in the mouse gut. Nat Biotechnol. 2023:1–10. 37985876 10.1038/s41587-023-01988-1 PMC11392810

[bib214] Lowery NV, McNally L, Ratcliff WC et al. Division of labor, bet hedging, and the evolution of mixed biofilm investment strategies. mBio. 2017;8:e00672–17. 10.1128/mBio.00672-17. 28790201 PMC5550747

[bib215] Lozupone CA, Knight R. Global patterns in bacterial diversity. Proc Natl Acad Sci USA. 2007;104:11436–40. 10.1073/pnas.0611525104. 17592124 PMC2040916

[bib216] Luo N, Lu J, Simsek E et al. The collapse of cooperation during range expansion of Pseudomonas aeruginosa. Nat Microbiol. 2024;9:1220–30. 10.1038/s41564-024-01627-8. 38443483 PMC7615952

[bib217] Ma Y, Kan A, Johnson DR. Metabolic interactions control the transfer and spread of plasmid-encoded antibiotic resistance during surface-associated microbial growth. Cell Rep. 2024;43.114653. 10.1016/j.celrep.2024.114653. 39213158

[bib218] Ma Y, Ramoneda J, Johnson DR. Timing of antibiotic administration determines the spread of plasmid-encoded antibiotic resistance during microbial range expansion. Nat Commun. 2023;14:3530. 10.1038/s41467-023-39354-z. 37316482 PMC10267205

[bib219] Machado D, Maistrenko OM, Andrejev S et al. Polarization of microbial communities between competitive and cooperative metabolism. Nat Ecol Evol. 2021;5:195–203. 10.1038/s41559-020-01353-4. 33398106 PMC7610595

[bib220] Mahler L, Niehs SP, Martin K et al. Highly parallelized droplet cultivation and prioritization of antibiotic producers from natural microbial communities. Kana BD, Dhar N, Sclavi B (eds.). eLife. 2021;10:e64774. 10.7554/eLife.64774. 33764297 PMC8081529

[bib221] Mant D, Orevi T, Kashtan N. Impact of micro-habitat fragmentation on microbial population growth dynamics. ISME J. 2025;19:wrae256. 10.1093/ismejo/wrae256. 39711055 PMC11964898

[bib222] Mark Welch JL, Rossetti BJ, Rieken CW et al. Biogeography of a human oral microbiome at the micron scale. Proc Natl Acad Sci USA. 2016;113:E791–800. 10.1073/pnas.1522149113. 26811460 PMC4760785

[bib374] Mark Welch JL, Dewhirst FE, Borisy GG. Biogeography of the oral microbiome: the site-specialist hypothesis. Annu Rev Microbiol. 2019;73:335–58. 10.1146/annurev-micro-090817-062503. 31180804 PMC7153577

[bib223] Martínez-Pérez C, Zweifel ST, Pioli R et al. Space, the final frontier: the spatial component of phytoplankton–bacterial interactions. Mol Microbiol. 2024;122:331–46. 38970428 10.1111/mmi.15293

[bib224] Martinez-Rabert E, Amstel Cv, Smith C et al. Environmental and ecological controls of the spatial distribution of microbial populations in aggregates. PLOS Comput Biol. 2022;18:e1010807. 10.1371/journal.pcbi.1010807. 36534694 PMC9810174

[bib225] Martiny JBH, Jones SE, Lennon JT et al. Microbiomes in light of traits: a phylogenetic perspective. Science. 2015;350:aac9323. 10.1126/science.aac9323. 26542581

[bib226] Mascarenhas R, Ruziska FM, Moreira EF et al. Integrating computational methods to investigate the macroecology of microbiomes. Front Genet. 2020;10. 10.3389/fgene.2019.01344. PMC6979972 32010196

[bib227] Mattingly HH, Emonet T. Collective behavior and nongenetic inheritance allow bacterial populations to adapt to changing environments. Proc Natl Acad Sci USA. 2022;119:e2117377119. 10.1073/pnas.2117377119. 35727978 PMC9245662

[bib228] McCallum G, Tropini C. The gut microbiota and its biogeography. Nat Rev Micro. 2024;22:105–18. 10.1038/s41579-023-00969-0. 37740073

[bib229] Medaney F, Dimitriu T, Ellis RJ et al. Live to cheat another day: bacterial dormancy facilitates the social exploitation of β-lactamases. ISME J. 2016;10:778–87. 10.1038/ismej.2015.154. 26505830 PMC4817691

[bib230] Meijer J, Skiadas P, Rainey PB et al. Eco-evolutionary dynamics of massive, parallel bacteriophage outbreaks in compost communities. 2025:bioRxiv. 2023.07.31.550844. 10.1101/2023.07.31.550844. PMC13220884 42213849

[bib231] Meijer J, van Dijk B, Hogeweg P. Contingent evolution of alternative metabolic network topologies determines whether cross-feeding evolves. Commun Biol. 2020;3:401. 10.1038/s42003-020-1107-x. 32728180 PMC7391776

[bib232] Melaugh G, Hutchison J, Kragh KN et al. Shaping the growth behaviour of biofilms initiated from bacterial aggregates. PLoS One. 2016;11:e0149683. 10.1371/journal.pone.0149683. 26934187 PMC4774936

[bib233] Melaugh G, Martinez VA, Baker P et al. Distinct types of multicellular aggregates in Pseudomonas aeruginosa liquid cultures. npj Biofilms Microbiomes. 2023;9:52. 10.1038/s41522-023-00412-5. 37507436 PMC10382557

[bib234] Melbourne BA, Chesson P. The scale transition: scaling up population dynamics with field data. Ecology. 2006;87:1478–88. 10.1890/0012-9658(2006)87[1478:TSTSUP]2.0.CO;2. 16869424

[bib235] Michaelis C, Grohmann E. Horizontal gene transfer of antibiotic resistance genes in biofilms. Antibiot Basel Switz. 2023;12:328. 10.3390/antibiotics12020328 PMC9952180 36830238

[bib236] Mohanty BK, Kushner SR Bacterial. /archaeal/organellar polyadenylation. WIREs RNA. 2011;2:256–76. 10.1002/wrna.51. PMC3041983 21344039

[bib237] Momeni B, Waite AJ, Shou W. Spatial self-organization favors heterotypic cooperation over cheating. Tautz D (ed.). eLife. 2013;2:e00960. 10.7554/eLife.00960. 24220506 PMC3823188

[bib238] Moreno-Gamez S, Hochberg ME, van Doorn GS. Quorum sensing as a mechanism to harness the wisdom of the crowds. Nat Commun. 2023;14:3415. 10.1038/s41467-023-37950-7. 37296108 PMC10256802

[bib239] Morris JJ, Lenski RE, Zinser ER. The black Queen hypothesis: evolution of dependencies through adaptive gene loss. mBio. 2012;3:e00036–12. 10.1128/mBio.00036-12. 22448042 PMC3315703

[bib240] Moses L, Pachter L. Museum of spatial transcriptomics. Nat Methods. 2022;19:534–46. 10.1038/s41592-022-01409-2. 35273392

[bib241] Mukherjee A, Ealy J, Huang Y et al. Coexisting ecotypes in long-term evolution emerged from interacting trade-offs. Nat Commun. 2023;14:3805. 10.1038/s41467-023-39471-9. 37365188 PMC10293278

[bib242] Müller MJI, Neugeboren BI, Nelson DR et al. Genetic drift opposes mutualism during spatial population expansion. Proc Natl Acad Sci USA. 2014;111:1037–42. 24395776 10.1073/pnas.1313285111 PMC3903240

[bib243] Mund A, Diggle SP, Harrison F. The fitness of Pseudomonas aeruginosa quorum sensing signal cheats is influenced by the diffusivity of the environment. mBio. 2017;8. 10.1128/mbio.00353-17. PMC5414003 28465424

[bib244] Muratore D, Boysen AK, Harke MJ et al. Complex marine microbial communities partition metabolism of scarce resources over the diel cycle. Nat Ecol Evol. 2022;6:218–29. 10.1038/s41559-021-01606-w. 35058612

[bib245] Nadel O, Hanna R, Rozenberg A et al. Viral NblA proteins negatively affect oceanic cyanobacterial photosynthesis. Nature. 2025;648:434–42. 10.1038/s41586-025-09656-x. 41224996 PMC12695635

[bib246] Nadell CD, Drescher K, Foster KR. Spatial structure, cooperation and competition in biofilms. Nat Rev Micro. 2016;14:589–600. 10.1038/nrmicro.2016.84. 27452230

[bib247] Naiman RJ, Johnston CA, Kelley JC. Alteration of North American streams by Beaver. Bioscience. 1988;38:753–62. 10.2307/1310784.

[bib248] Narayanasamy N, Bingham E, Fadero T et al. Metabolically driven flows enable exponential growth in macroscopic multicellular yeast. Sci Adv. 2025;11:eadr6399. 10.1126/sciadv.adr6399. 40540574 PMC12180493

[bib249] Nazir SA, van Dijk B. Viral lifecycle dynamics and spatial structure explain why pathogenicity is prophage-encoded. bioRxiv. 2025:2025.02.28.640786. 10.1101/2025.02.28.640786. PMC13427762 42455142

[bib250] Nguyen TTH, Zakem EJ, Ebrahimi A et al. Microbes contribute to setting the ocean carbon flux by altering the fate of sinking particulates. Nat Commun. 2022;13:1657. 10.1038/s41467-022-29297-2. 35351873 PMC8964765

[bib251] Nowak M. An evolutionarily stable strategy may Be inaccessible. J Theor Biol. 1990;142:237–41. 10.1016/S0022-5193(05)80224-3. 2352434

[bib252] Nowak MA, May RM. Evolutionary games and spatial chaos. Nature. 1992;359:826–9. 10.1038/359826a0.

[bib253] Odling-Smee FJ, Laland KN, Feldman MW. Niche Construction. Am Nat. 1996;147:641–8. 10.1086/285870.

[bib254] Okasha S. Altruism, group selection and correlated interaction. Br J Philos Sci. 2005;56:703–25. 10.1093/bjps/axi143.

[bib255] Oldroyd GED, Murray JD, Poole PS et al. The rules of engagement in the legume-rhizobial symbiosis. Annu Rev Genet. 2011;45:119–44. 10.1146/annurev-genet-110410-132549. 21838550

[bib256] Oliveira NM, Niehus R, Foster KR. Evolutionary limits to cooperation in microbial communities. Proc Natl Acad Sci USA. 2014;111:17941–6. 10.1073/pnas.1412673111. 25453102 PMC4273359

[bib257] Ona L, Giri S, Avermann N et al. Obligate cross-feeding expands the metabolic niche of bacteria. Nat Ecol Evol. 2021;5:1224–32. 10.1038/s41559-021-01505-0. 34267366

[bib258] Orgogozo V. Replaying the tape of life in the twenty-first century. Interface Focus. 2015;5:20150057. 10.1098/rsfs.2015.0057. 26640652 PMC4633862

[bib259] Pagie L, Hogeweg P. Colicin diversity: a result of eco-evolutionary dynamics. J Theor Biol. 1999;196:251–261. 10.1006/jtbi.1998.0838. 10049618

[bib260] Pagie L, Hogeweg P. Individual- and population-based diversity in restriction-modification systems. Bull Math Biol. 2000;62:759–74. 10.1006/bulm.2000.0177. 10938631

[bib261] Palmer JD, Foster KR. Bacterial species rarely work together. Science. 2022;376:581–2. 10.1126/science.abn5093. 35511986

[bib262] Pamp SJ, Sternberg C, Tolker-Nielsen T. Insight into the microbial multicellular lifestyle via flow-cell technology and confocal microscopy. Cytometry A. 2009;75A:90–103. 10.1002/cyto.a.20685. 19051241

[bib263] Pande S, Kost C. Bacterial unculturability and the formation of intercellular metabolic networks. Trends Microbiol. 2017;25:349–61. 10.1016/j.tim.2017.02.015. 28389039

[bib264] Pathak A, Angst DC, Leon-Sampedro R et al. Antibiotic-degrading resistance changes bacterial community structure via species-specific responses. ISME J. 2023;17:1495–503. 10.1038/s41396-023-01465-2. 37380830 PMC10432403

[bib265] Pavlogiannis A, Tkadlec J, Chatterjee K et al. Construction of arbitrarily strong amplifiers of natural selection using evolutionary graph theory. Commun Biol. 2018;1:71. 10.1038/s42003-018-0078-7. 30271952 PMC6123726

[bib266] Peets P, Litos A, Dührkop K et al. Chemical characteristics vectors map the chemical space of natural biomes from untargeted mass spectrometry data. J Cheminformatics. 2025;17:82. 10.1186/s13321-025-01031-2. PMC12107775 40420312

[bib398_191_132426] Peischl S, Dupanloup I, Kirkpatrick M and Excoffier L. On the accumulation of deleterious mutations during range expansions. Mol Ecol. 2013;22:5972–82. 10.1111/mec.12524. 24102784

[bib267] Pereira CS, Thompson JA, Xavier KB. AI-2-mediated signalling in bacteria. FEMS Microbiol Rev. 2013;37:156–81. 10.1111/j.1574-6976.2012.00345.x. 22712853

[bib268] Pherribo GJ, Taga ME. Bacteriophage-mediated lysis supports robust growth of amino acid auxotrophs. ISME J. 2023;17:1785–8. 10.1038/s41396-023-01452-7. 37322284 PMC10504361

[bib269] Philippot L, Chenu C, Kappler A et al. The interplay between microbial communities and soil properties. Nat Rev Micro. 2024;22:226–39. 10.1038/s41579-023-00980-5. 37863969

[bib270] Piel D, Bruto M, Labreuche Y et al. Phage-host coevolution in natural populations. Nat Microbiol. 2022;7:1075–86. 10.1038/s41564-022-01157-1. 35760840

[bib271] Pollak S, Gralka M, Sato Y et al. Public good exploitation in natural bacterioplankton communities. Sci Adv. 2021;7:eabi4717. 10.1126/sciadv.abi4717. 34321201 PMC8318375

[bib272] Pontrelli S, Szabo R, Pollak S et al. Metabolic cross-feeding structures the assembly of polysaccharide degrading communities. Sci Adv. 2022;8:eabk3076. 10.1126/sciadv.abk3076. 35196097 PMC8865766

[bib273] Raina JB, Giardina M, Brumley DR et al. Chemotaxis increases metabolic exchanges between marine picophytoplankton and heterotrophic bacteria. Nat Microbiol. 2023;8:510–21. 36759754 10.1038/s41564-023-01327-9

[bib274] Raina JB, Lambert BS, Parks DH et al. Chemotaxis shapes the microscale organization of the ocean’s microbiome. Nature. 2022;605:132–8. 10.1038/s41586-022-04614-3. 35444277

[bib275] Rainey PB, Quistad SD. Toward a dynamical understanding of microbial communities. Philos Trans R Soc B Biol Sci. 2020;375:20190248. 10.1098/rstb.2019.0248. PMC7133524 32200735

[bib276] Rainey PB, Travisano M. Adaptive radiation in a heterogeneous environment. Nature. 1998;394:69–72. 10.1038/27900. 9665128

[bib277] Ramesh D, Fara E, Oschmann F et al. When less is not more: limits to the evolution of metabolic dependence in spatially structured microbial communities. bioRxiv. 2025:2025.05.09.651753. 10.1101/2025.05.09.651753. PMC12936941 41767253

[bib278] Rani G, Sengupta A. Growing bacterial colonies harness emergent genealogical demixing to regulate organizational entropy. Biophys Rep. 2024;4.100175. 10.1016/j.bpr.2024.100175. PMC11416667 39197679

[bib279] Rankin DJ, Rocha EP, Brown SP. What traits are carried on mobile genetic elements, and why?. Heredity. 2011;106:1–10. 10.1038/hdy.2010.24. 20332804 PMC3183850

[bib280] Ratcliff WC, Denison RF, Borrello M et al. Experimental evolution of multicellularity. Proc Natl Acad Sci USA. 2012;109:1595–600. 10.1073/pnas.1115323109. 22307617 PMC3277146

[bib281] Ratcliff WC, Fankhauser JD, Rogers DW et al. Origins of multicellular evolvability in snowflake yeast. Nat Commun. 2015;6:6102. 10.1038/ncomms7102. 25600558 PMC4309424

[bib282] Ratzke C, Barrere J, Gore J. Strength of species interactions determines biodiversity and stability in microbial communities. Nat Ecol Evol. 2020;4:376–83. 10.1038/s41559-020-1099-4. 32042124

[bib283] Ratzke C, Gore J. Modifying and reacting to the environmental pH can drive bacterial interactions. PLoS Biol. 2018;16:e2004248. 10.1371/journal.pbio.2004248. 29538378 PMC5868856

[bib284] Raynaud X, Nunan N. Spatial ecology of bacteria at the microscale in soil. PLoS One. 2014;9:e87217. 10.1371/journal.pone.0087217. 24489873 PMC3905020

[bib285] Redfield RJ. Is quorum sensing a side effect of diffusion sensing?. Trends Microbiol. 2002;10:365–70. 10.1016/S0966-842X(02)02400-9. 12160634

[bib286] Reyes-Robles T, Dillard RS, Cairns LS et al. *Vibrio cholerae* Outer membrane vesicles inhibit bacteriophage infection. J Bacteriol. 2018;200:e00792–17. 10.1128/jb.00792-17. 29661863 PMC6040182

[bib287] Reynolds CW. Flocks, herds and schools: a distributed behavioral model. Proceedings of the 14th Annual Conference on Computer Graphics and Interactive Techniques. New York, NY, USA: Association for Computing Machinery, 1987,25–34. 10.1145/37401.37406.

[bib288] Rillig MC, Muller LAH, Lehmann A. Soil aggregates as massively concurrent evolutionary incubators. ISME J. 2017;11:1943–8. 10.1038/ismej.2017.56. 28409772 PMC5563948

[bib289] Robert L, Paul G, Chen Y et al. Pre-dispositions and epigenetic inheritance in the Escherichia coli lactose operon bistable switch. Mol Syst Biol. 2010;6:357. 10.1038/msb.2010.12. 20393577 PMC2872608

[bib290] Rodriguez-Valera F, Martin-Cuadrado A-B, Rodriguez-Brito B et al. Explaining microbial population genomics through phage predation. Nat Rev Micro. 2009;7:828–36. 10.1038/nrmicro2235. 19834481

[bib291] Romeyer Dherbey J, Parab L, Gallie J et al. Stepwise evolution of E. coli C and ΦX174 reveals unexpected lipopolysaccharide (LPS) diversity. Mol Biol Evol. 2023;40:msad154. 10.1093/molbev/msad154. 37399035 PMC10368449

[bib292] Rosa-Masegosa A, Rodriguez-Sanchez A, Gorrasi S et al. Microbial ecology of granular biofilm technologies for wastewater treatment: a review. Microorganisms. 2024;12:433. 10.3390/microorganisms12030433. 38543484 PMC10972187

[bib293] Rozen DE, Lenski RE. Long-term experimental evolution in.: VIII.: dynamics of a balanced polymorphism. Am Nat. 2000;155:24–35. 10.1086/303299. 10657174

[bib294] Ruan C, Ramoneda J, Gogia G et al. Fungal hyphae regulate bacterial diversity and plasmid-mediated functional novelty during range expansion. Curr Biol. 2022;32:5285–5294 e4. 10.1016/j.cub.2022.11.009. 36455559

[bib295] Ruan C, Ramoneda J, Kan A et al. Phage predation accelerates the spread of plasmid-encoded antibiotic resistance. Nat Commun. 2024;15:5397. 10.1038/s41467-024-49840-7. 38926498 PMC11208555

[bib296] Ruan C, Vinod DP, Johnson DR. Phage-mediated peripheral kill-the-winner facilitates the maintenance of costly antibiotic resistance. Nat Commun. 2025;16:5839. 10.1038/s41467-025-61055-y. 40592899 PMC12219745

[bib297] Rulands S, Jahn D, Frey E. Specialization and bet hedging in heterogeneous populations. Phys Rev Lett. 2014;113:108102. 10.1103/PhysRevLett.113.108102. 25238387

[bib298] Saarenpaa S, Shalev O, Ashkenazy H et al. Spatial metatranscriptomics resolves host-bacteria-fungi interactomes. Nat Biotechnol. 2023:1–10. 37985875 10.1038/s41587-023-01979-2 PMC11392817

[bib299] Salazar G, Cornejo-Castillo FM, Benítez-Barrios V et al. Global diversity and biogeography of deep-sea pelagic prokaryotes. ISME J. 2016;10:596–608. 10.1038/ismej.2015.137. 26251871 PMC4817678

[bib300] Sánchez-Peña A, Winans JB, Nadell CD et al. Pseudomonas aeruginosa surface motility and invasion into competing communities enhance interspecies antagonism. mBio. 2024;15:e00956–24. 39105585 10.1128/mbio.00956-24 PMC11389416

[bib301] Savage VJ, Chopra I, O’Neill AJ. Staphylococcus aureus biofilms promote horizontal transfer of antibiotic resistance. Antimicrob Agents Chemother. 2013;57:1968–70. 10.1128/AAC.02008-12. 23357771 PMC3623343

[bib302] Scheidweiler D, Bordoloi AD, Jiao W et al. Spatial structure, chemotaxis and quorum sensing shape bacterial biomass accumulation in complex porous media. Nat Commun. 2024;15:191. 10.1038/s41467-023-44267-y. 38167276 PMC10761857

[bib303] Schink SJ, Christodoulou D, Mukherjee A et al. Glycolysis/gluconeogenesis specialization in microbes is driven by biochemical constraints of flux sensing. Mol Syst Biol. 2022;18:e10704. 10.15252/msb.202110704. 34994048 PMC8738977

[bib304] Schirrmeister BE, Gugger M, Donoghue PCJ. Cyanobacteria and the Great Oxidation Event: evidence from genes and fossils. Palaeontology. 2015;58:769–85. 10.1111/pala.12178. 26924853 PMC4755140

[bib305] Schluter J, Schoech AP, Foster KR et al. The evolution of quorum sensing as a mechanism to infer kinship. PLoS Comput Biol. 2016;12:e1004848. 10.1371/journal.pcbi.1004848. 27120081 PMC4847791

[bib306] Schmickl T, Stefanec M, Crailsheim K. How a life-like system emerges from a simplistic particle motion law. Sci Rep. 2016;6:37969. 10.1038/srep37969. 27901107 PMC5346932

[bib307] Schmidt SK, Lynch RC, King AJ et al. Phylogeography of microbial phototrophs in the dry valleys of the high Himalayas and Antarctica. Proc. R. Soc. B. Biol. Sci. 2010;278:702–8. 10.1098/rspb.2010.1254. PMC3030841 20826485

[bib308] Schreiber SJ, Killingback TP. Spatial heterogeneity promotes coexistence of rock-paper-scissors metacommunities. Theor Popul Biol. 2013;86:1–11. 10.1016/j.tpb.2013.02.004. 23474219

[bib309] Schwarcz D, Levine H, Ben-Jacob E et al. Uniform modeling of bacterial colony patterns with varying nutrient and substrate. Phys Nonlinear Phenom. 2016;318–319:91–9. 10.1016/j.physd.2015.11.002.

[bib310] Schwartzman JA, Ebrahimi A, Chadwick G et al. Bacterial growth in multicellular aggregates leads to the emergence of complex life cycles. Curr Biol. 2022;32:3059–3069 e7. 10.1016/j.cub.2022.06.011. 35777363 PMC9496226

[bib311] Schwechheimer C, Kuehn MJ. Outer-membrane vesicles from gram-negative bacteria: biogenesis and functions. Nat Rev Micro. 2015;13:605–19. 10.1038/nrmicro3525. PMC5308417 26373371

[bib312] Scott-Phillips TC, Laland KN, Shuker DM et al. The niche construction perspective: a critical appraisal. Evolution. 2014;68:1231–43. 10.1111/evo.12332. 24325256 PMC4261998

[bib313] Secor PR, Michaels LA, Ratjen A et al. Entropically driven aggregation of bacteria by host polymers promotes antibiotic tolerance in Pseudomonas aeruginosa. Proc Natl Acad Sci USA. 2018;115:10780–5. 10.1073/pnas.1806005115. 30275316 PMC6196481

[bib314] Seymour JR, Brumley DR, Stocker R et al. Swimming towards each other: the role of chemotaxis in bacterial interactions. Trends Microbiol. 2024;32:640–9. 10.1016/j.tim.2023.12.008. 38212193

[bib315] Shaer Tamar E, Kishony R. Multistep diversification in spatiotemporal bacterial-phage coevolution. Nat Commun. 2022;13:7971. 10.1038/s41467-022-35351-w. 36577749 PMC9797572

[bib316] Shaffer JP, Nothias LF, Thompson LR et al. Standardized multi-omics of Earth’s microbiomes reveals microbial and metabolite diversity. Nat Microbiol. 2022;7:2128–50. 10.1038/s41564-022-01266-x. 36443458 PMC9712116

[bib317] Sharma A, Wood KB. Spatial segregation and cooperation in radially expanding microbial colonies under antibiotic stress. ISME J. 2021;15:3019–33. 10.1038/s41396-021-00982-2. 33953363 PMC8443724

[bib318] Shi H, Shi QJ, Grodner B et al. Highly multiplexed spatial mapping of microbial communities. Nature. 2020;588:676–681. 10.1038/s41586-020-2983-4. 33268897 PMC8050837

[bib319] Shibasaki S, Mitri S. A spatially structured mathematical model of the gut microbiome reveals factors that increase community stability. iScience. 2023;26:107499. 10.1016/j.isci.2023.107499. 37670791 PMC10475486

[bib320] Silpe JE, Bassler BL. A host-produced quorum-sensing autoinducer controls a phage lysis-lysogeny decision. Cell. 2019;176:268–80.e13. 10.1016/j.cell.2018.10.059. 30554875 PMC6329655

[bib321] Silveira CB, Rohwer FL. Piggyback-the-winner in host-associated microbial communities. npj Biofilms Microbiomes. 2016;2:16010. 10.1038/npjbiofilms.2016.10. 28721247 PMC5515262

[bib322] Simmons EL, Drescher K, Nadell CD et al. Phage mobility is a core determinant of phage–bacteria coexistence in biofilms. ISME J. 2018;12:532–43. 10.1038/ismej.2017.190. PMC5776469 29125597

[bib323] Siqueira T, Saito VS, Bini LM et al. Community size can affect the signals of ecological drift and niche selection on biodiversity. Ecology. 2020;101:e03014. 10.1002/ecy.3014. 32068259

[bib324] Smercina DN, Bailey VL, Hofmockel KS. Micro on a macroscale: relating microbial-scale soil processes to global ecosystem function. FEMS Microbiol Ecol. 2021;97. 10.1093/femsec/fiab091. 34223869

[bib325] Smith WPJ, Brodmann M, Unterweger D et al. The evolution of tit-for-tat in bacteria via the type VI secretion system. Nat Commun. 2020a;11:5395. 10.1038/s41467-020-19017-z. 33106492 PMC7589516

[bib326] Smith WPJ, Vettiger A, Winter J et al. The evolution of the type VI secretion system as a disintegration weapon. PLoS Biol. 2020b;18:e3000720. 10.1371/journal.pbio.3000720. 32453732 PMC7274471

[bib327] Smriga S, Fernandez VI, Mitchell JG et al. Chemotaxis toward phytoplankton drives organic matter partitioning among marine bacteria. Proc Natl Acad Sci USA. 2016;113:1576–81. 10.1073/pnas.1512307113. 26802122 PMC4760798

[bib328] Sokol NW, Slessarev E, Marschmann GL et al. Life and death in the soil microbiome: how ecological processes influence biogeochemistry. Nat Rev Micro. 2022;20:415–30. 10.1038/s41579-022-00695-z. 35228712

[bib329] Sorg RA, Lin L, Doorn GSv et al. Collective resistance in microbial communities by intracellular antibiotic deactivation. PLOS Biol. 2016;14:e2000631. 10.1371/journal.pbio.2000631. 28027306 PMC5189934

[bib330] Souza CP, Almeida BC, Colwell RR et al. The importance of chitin in the marine environment. Mar Biotechnol N Y N. 2011;13:823–30. 10.1007/s10126-011-9388-1. 21607543

[bib331] Speth DR, Zandt MH, Guerrero-Cruz S et al. Genome-based microbial ecology of anammox granules in a full-scale wastewater treatment system. Nat Commun. 2016;7:11172. 10.1038/ncomms11172. 27029554 PMC4821891

[bib332] Stocker R, Seymour JR, Samadani A et al. Rapid chemotactic response enables marine bacteria to exploit ephemeral microscale nutrient patches. Proc Natl Acad Sci USA. 2008;105:4209–14. 10.1073/pnas.0709765105. 18337491 PMC2393791

[bib333] Stocker R. Marine microbes see a sea of gradients. Science. 2012;338:628–33. 10.1126/science.1208929. 23118182

[bib334] Stubbusch AKM, Keegstra JM, Schwartzman J et al. Polysaccharide breakdown products drive degradation-dispersal cycles of foraging bacteria through changes in metabolism and motility. Momeni B, Weigel D (eds.). eLife. 2024;13:RP93855. 10.7554/eLife.93855. 39429128 PMC11493405

[bib335] Stubbusch AKM, Peaudecerf FJ, Lee KS et al. Antagonism as a foraging strategy in microbial communities. Science. 2025;388:1214–7. 10.1126/science.adr8286. 40504916

[bib336] Stump SM, Johnson EC, Sun Z et al. How spatial structure and neighbor uncertainty promote mutualists and weaken black queen effects. J Theor Biol. 2018;446:33–60. 10.1016/j.jtbi.2018.02.031. 29499252

[bib337] Takeuchi N, Fullmer MS, Maddock DJ et al. The constructive Black Queen hypothesis: new functions can evolve under conditions favouring gene loss. ISME J. 2024;18:wrae011. 10.1093/ismejo/wrae011. 38366199 PMC10942775

[bib338] Tan D, Hansen MF, de Carvalho LN et al. High cell densities favor lysogeny: induction of an H20 prophage is repressed by quorum sensing and enhances biofilm formation in Vibrio anguillarum. ISME J. 2020;14:1731–42. 10.1038/s41396-020-0641-3. 32269377 PMC7305317

[bib339] Tavaddod S, Dawson A, Allen RJ. Bacterial aggregation triggered by low-level antibiotic-mediated lysis. npj Biofilms Microbiomes. 2024;10:1–12. 10.1038/s41522-024-00553-1. 39327479 PMC11427687

[bib340] Tecon R, Mitri S, Ciccarese D et al. Bridging the holistic-reductionist divide in microbial ecology. Msystems. 2019;4:e00265–18. 10.1128/msystems.00265-18. 30746494 PMC6365645

[bib341] Thompson LR, Sanders JG, McDonald D et al. A communal catalogue reveals Earth’s multiscale microbial diversity. Nature. 2017;551:457–463. 10.1038/nature24621. 29088705 PMC6192678

[bib342] Totsche KU, Amelung W, Gerzabek MH et al. Microaggregates in soils. J Plant Nutr Soil Sci. 2018;181:104–36. 10.1002/jpln.201600451.

[bib343] Tronnolone H, Tam A, Szenczi Z et al. Diffusion-limited growth of microbial colonies. Sci Rep. 2018;8:5992. 10.1038/s41598-018-23649-z. 29662092 PMC5902472

[bib344] Tsai H-H, Tang Y, Jiang L et al. Localized glutamine leakage drives the spatial structure of root microbial colonization. Science. 2025;390:eadu4235. 10.1126/science.adu4235. 41037624

[bib345] Ugolini GS, Wang M, Secchi E et al. Microfluidic approaches in microbial ecology. Lab Chip. 2024;24:1394–418. 10.1039/D3LC00784G. 38344937 PMC10898419

[bib346] Urquhart AS, Forsythe A, Vogan AA. Are fungal disease outbreaks instigated by starship transposons?. Mol Plant Pathol. 2025;26:e70124. 10.1111/mpp.70124. 40657948 PMC12257634

[bib347] Valm AM, Mark Welch JL, Borisy GG. CLASI-FISH: principles of combinatorial labeling and spectral imaging. Syst Appl Microbiol. 2012;35:496–502. 10.1016/j.syapm.2012.03.004. 22521573 PMC3407316

[bib349] van Dijk B, Bertels F, Stolk L et al. Transposable elements promote the evolution of genome streamlining. Philos Trans R Soc Lond B Biol Sci. 2022;377:20200477. 10.1098/rstb.2020.0477. 34839699 PMC8628081

[bib350] van Dijk B, Hogeweg P, Doekes HM et al. Slightly beneficial genes are retained by bacteria evolving DNA uptake despite selfish elements. eLife. 2020;9:e56801. 10.7554/eLife.56801. 32432548 PMC7316506

[bib351] van Dijk B, Hogeweg P. In Silico gene-level evolution explains microbial population diversity through differential gene mobility. Genome Biol Evol. 2015;8:176–88. 10.1093/gbe/evv255. 26710854 PMC4758251

[bib352] van Dijk B, Meijer J, Cuypers TD et al. Trusting the hand that feeds: microbes evolve to anticipate a serial transfer protocol as individuals or collectives. BMC Evol Biol. 2019;19:201. 10.1186/s12862-019-1512-2. 31684861 PMC6829849

[bib354] van Dijk B. Cacatoo: building, exploring, and sharing spatially structured models of biological systems. J Open Source Softw. 2022;7:3948. 10.21105/joss.03948.

[bib353] van Dijk B. Can mobile genetic elements rescue genes from extinction?. Curr Genet. 2020;66:1069–71. 10.1007/s00294-020-01104-9. 32880674 PMC7599165

[bib355] van Gestel J, Bareia T, Tenennbaum B et al. Short-range quorum sensing controls horizontal gene transfer at micron scale in bacterial communities. Nat Commun. 2021;12:2324. 10.1038/s41467-021-22649-4. 33875666 PMC8055654

[bib356] van Gestel J, Weissing FJ. Regulatory mechanisms link phenotypic plasticity to evolvability. Sci Rep. 2016;6:24524. 10.1038/srep24524. 27087393 PMC4834480

[bib357] van Tatenhove-Pel RJ, de Groot DH, Bisseswar AS et al. Population dynamics of microbial cross-feeding are determined by co-localization probabilities and cooperation-independent cheater growth. ISME J. 2021a;15:3050–61. 10.1038/s41396-021-00986-y. 33953364 PMC8443577

[bib358] van Tatenhove-Pel RJ, Rijavec T, Lapanje A et al. Microbial competition reduces metabolic interaction distances to the low µm-range. ISME J. 2021b;15:688–701. 10.1038/s41396-020-00806-9. 33077887 PMC8027890

[bib359] van Vliet DM, von Meijenfeldt FAB, Dutilh BE et al. The bacterial sulfur cycle in expanding dysoxic and euxinic marine waters. Environ Microbiol. 2021;23:2834–57. 10.1111/1462-2920.15265. 33000514 PMC8359478

[bib360] van Vliet S, Dal Co A, Winkler AR et al. Spatially correlated gene expression in bacterial groups: the role of lineage history, spatial gradients, and cell-cell interactions. Cell Syst. 2018;6:496–507.e6. 10.1016/j.cels.2018.03.009. 29655705 PMC6764841

[bib361] van Vliet S, Hauert C, Fridberg K et al. Global dynamics of microbial communities emerge from local interaction rules. PLoS Comput Biol. 2022;18:e1009877. 10.1371/journal.pcbi.1009877. 35245282 PMC8926250

[bib348] Vandereyken K, Sifrim A, Thienpont B et al. Methods and applications for single-cell and spatial multi-omics. Nat Rev Genet. 2023;24:494–515. 10.1038/s41576-023-00580-2. 36864178 PMC9979144

[bib362] Veening JW, Stewart EJ, Berngruber TW et al. Bet-hedging and epigenetic inheritance in bacterial cell development. Proc Natl Acad Sci USA. 2008;105:4393–8. 10.1073/pnas.0700463105. 18326026 PMC2393751

[bib363] Vellend M. Conceptual Synthesis in Community Ecology. Q Rev Biol. 2010;85:183–206. 10.1086/652373. 20565040

[bib364] Verdon N, Popescu O, Titmuss S et al. Habitat fragmentation enhances microbial collective defence. J R Soc Interface. 2025;22:20240611. 10.1098/rsif.2024.0611. 39933594 PMC11813583

[bib365] Vetsigian K. Diverse modes of eco-evolutionary dynamics in communities of antibiotic-producing microorganisms. Nat Ecol Evol. 2017;1:0189. 10.1038/s41559-017-0189.

[bib366] Vicsek T, Czirók A, Ben-Jacob E et al. Novel type of phase transition in a system of self-driven particles. Phys Rev Lett. 1995;75:1226–9. 10.1103/PhysRevLett.75.1226. 10060237

[bib367] von Meijenfeldt FAB, Hogeweg P, Dutilh BE. A social niche breadth score reveals niche range strategies of generalists and specialists. Nat Ecol Evol. 2023;7:768–81. 10.1038/s41559-023-02027-7. 37012375 PMC10172124

[bib368] Vos M, Birkett PJ, Birch E et al. Local adaptation of bacteriophages to their bacterial hosts in soil. Science. 2009;325:833. 10.1126/science.1174173. 19679806

[bib369] Wang J, Hashem I, Bhonsale S et al. Individual-based modelling (IbM) in food microbiology: a comprehensive guideline. Food Res Int. 2025a;213:116408. 10.1016/j.foodres.2025.116408. 40436598

[bib370] Wang Q, Wei S, Madsen JS. Cooperative resistance varies among β-lactamases in E. coli, with some enabling cross-protection and sustained extracellular activity. Commun Biol. 2025b;8:968. 10.1038/s42003-025-08392-2. 40595357 PMC12217783

[bib371] Wang T, Shen P, He Y et al. Spatial transcriptome uncovers rich coordination of metabolism in E. coli K12 biofilm. Nat Chem Biol. 2023;19:940–50. 10.1038/s41589-023-01282-w. 37055614

[bib372] Wang X. Overflow metabolism originates from growth optimization and cell heterogeneity. eLife. 2025. 10.7554/eLife.94586.4. PMC12140630 40472190

[bib373] Waters CM, Bassler BL. Quorum sensing: cell-to-cell communication in bacteria. Annu Rev Cell Dev Biol. 2005;21:319–46. 10.1146/annurev.cellbio.21.012704.131001. 16212498

[bib375] Werner J, Arndt H. Spatio-temporal pattern formation of living organisms at the edge of chaos. ISME J. 2025;19:wraf050. 10.1093/ismejo/wraf050. 40079679 PMC11964086

[bib376] West SA, Griffin AS, Gardner A et al. Social evolution theory for microorganisms. Nat Rev Micro. 2006;4:597–607. 10.1038/nrmicro1461. 16845430

[bib377] Westermann AJ, Vogel J. Cross-species RNA-seq for deciphering host–microbe interactions. Nat Rev Genet. 2021;22:361–78. 10.1038/s41576-021-00326-y. 33597744

[bib378] Wetherington MT, Nagy K, Der L et al. Ecological succession and the competition-colonization trade-off in microbial communities. BMC Biol. 2022;20:262. 10.1186/s12915-022-01462-5. 36447225 PMC9710175

[bib379] Whitchurch CB, Tolker-Nielsen T, Ragas PC et al. Extracellular DNA required for bacterial biofilm formation. Science. 2002;295:1487. 10.1126/science.295.5559.1487. 11859186

[bib380] Wienhausen G, Moraru C, Bruns S et al. Ligand cross-feeding resolves bacterial vitamin B(12) auxotrophies. Nature. 2024;629:886–92. 10.1038/s41586-024-07396-y. 38720071

[bib381] Wilpiszeski RL, Aufrecht JA, Retterer ST et al. Soil aggregate microbial communities: towards understanding microbiome interactions at biologically relevant scales. Appl Environ Microb. 2019;85:e00324–19. 10.1128/AEM.00324-19. PMC6606860 31076430

[bib382] Wilson DS. A theory of group selection. Proc Natl Acad Sci USA. 1975;72:143–6. 10.1073/pnas.72.1.143. 1054490 PMC432258

[bib383] Wolfsberg E, Long CP, Antoniewicz MR. Metabolism in dense microbial colonies: 13C metabolic flux analysis of *E. coli* grown on agar identifies two distinct cell populations with acetate cross-feeding. Metab Eng. 2018;49:242–7. 10.1016/j.ymben.2018.08.013. 30179665

[bib384] Wong W, Bravo P, Yunker PJ et al. Oxygen-binding proteins aid oxygen diffusion to enhance fitness of a yeast model of multicellularity. PLOS Biol. 2025;23:e3002975. 10.1371/journal.pbio.3002975. 39883703 PMC11781632

[bib385] Wu F, Ha Y, Weiss A et al. Modulation of microbial community dynamics by spatial partitioning. Nat Chem Biol. 2022;18:394–402. 10.1038/s41589-021-00961-w. 35145274 PMC8967799

[bib386] Wu Y, Fu C, Peacock CL et al. Cooperative microbial interactions drive spatial segregation in porous environments. Nat Commun. 2023;14:4226. 10.1038/s41467-023-39991-4. 37454222 PMC10349867

[bib387] Xiong LY, Cooper R, Tsimring LS. Coexistence and pattern formation in bacterial mixtures with contact-dependent killing. Biophys J. 2018;114:1741–50. 10.1016/j.bpj.2018.02.012. 29642042 PMC5954290

[bib388] Yip A, Smith-Roberge J, Khorasani SH et al. Calibrating spatiotemporal models of microbial communities to microscopy data: a review. PLoS Comput Biol. 2022;18:e1010533. 10.1371/journal.pcbi.1010533. 36227846 PMC9560168

[bib389] Young E, Allen RJ. Lineage dynamics in growing biofilms: spatial patterns of standing vs. de novo diversity. Front Microbiol. 2022;13:915095. 10.3389/fmicb.2022.915095. 35966660 PMC9363821

[bib390] Young E, Melaugh G, Allen RJ. Active layer dynamics drives a transition to biofilm fingering. npj Biofilms Microbiomes. 2023;9:1–12. 10.1038/s41522-023-00380-w. 37024470 PMC10079924

[bib391] Zelezniak A, Andrejev S, Ponomarova O et al. Metabolic dependencies drive species co-occurrence in diverse microbial communities. Proc Natl Acad Sci USA. 2015;112:6449–54. 10.1073/pnas.1421834112. 25941371 PMC4443341

[bib392] Zhalnina K, Zengler K, Newman D et al. Need for laboratory ecosystems to unravel the structures and functions of soil microbial communities mediated by chemistry. mBio. 2018;9. 10.1128/mbio.01175-18. PMC6050955 30018110

[bib393] Zhang L, Li S, Liu X et al. Sensing of autoinducer-2 by functionally distinct receptors in prokaryotes. Nat Commun. 2020;11:5371. 10.1038/s41467-020-19243-5. 33097715 PMC7584622

[bib394] Zhang Z, Shitut S, Claushuis B et al. Mutational meltdown of putative microbial altruists in Streptomyces coelicolor colonies. Nat Commun. 2022;13:2266. 10.1038/s41467-022-29924-y. 35477578 PMC9046218

[bib395] Zimmerman AE, Howard-Varona C, Needham DM et al. Metabolic and biogeochemical consequences of viral infection in aquatic ecosystems. Nat Rev Micro. 2020;18:21–34. 10.1038/s41579-019-0270-x. 31690825

[bib396] Zomer A, Ingham CJ, von Meijenfeldt FAB et al. Structural color in the bacterial domain: the ecogenomics of a 2-dimensional optical phenotype. Proc Natl Acad Sci USA. 2024;121:e2309757121. 10.1073/pnas.2309757121. 38990940 PMC11260094

